# Closing the gap: prognostic and predictive biomarker validation for personalized care in a Latin American hormone-dependent breast cancer cohort

**DOI:** 10.1093/oncolo/oyae191

**Published:** 2024-08-08

**Authors:** Daniela Alves da Quinta, Darío Rocha, Javier Retamales, Diego Giunta, Nora Artagaveytia, Carlos Velazquez, Adrian Daneri-Navarro, Bettina Müller, Eliana Abdelhay, Alicia I Bravo, Mónica Castro, Cristina Rosales, Elsa Alcoba, Gabriela Acosta Haab, Fernando Carrizo, Irene Sorin, Alejandro Di Sibio, Márcia Marques-Silveira, Renata Binato, Benedicta Caserta, Gonzalo Greif, Alicia Del Toro-Arreola, Antonio Quintero-Ramos, Jorge Gómez, Osvaldo L Podhajcer, Elmer A Fernández, LACRN Investigators, Andrea S Llera

**Affiliations:** Laboratorio de Terapia Molecular y Celular, Fundación Instituto Leloir-CONICET, Ciudad de Buenos Aires, Argentina; Universidad Argentina de la Empresa (UADE), Instituto de Tecnología (INTEC), Buenos Aires, Argentina; Universidad Nacional de Córdoba, Facultad de Ciencias Exactas, Físicas y Naturales, Córdoba, Argentina; Grupo Oncológico Cooperativo Chileno de Investigación, Santiago de Chile, Chile; Instituto Universitario Hospital Italiano de Buenos Aires-CONICET, Buenos Aires, Argentina; Hospital de Clínicas Manuel Quintela, Universidad de la República, Montevideo, Uruguay; Universidad de Sonora, Hermosillo, Mexico; Universidad de Guadalajara, Guadalajara, Mexico; Instituto Nacional del Cáncer, Santiago de Chile, Chile; Bone Marrow Transplantation Unit, Instituto Nacional de Câncer, Rio de Janeiro, RJ, Brazil; Hospital Regional de Agudos Eva Perón, San Martín, Provincia de Buenos Aires, Argentina; Instituto de Oncología Angel Roffo, Ciudad de Buenos Aires, Argentina; Hospital Municipal de Oncología María Curie, Ciudad de Buenos Aires, Argentina; Hospital Municipal de Oncología María Curie, Ciudad de Buenos Aires, Argentina; Hospital Municipal de Oncología María Curie, Ciudad de Buenos Aires, Argentina; Hospital Regional de Agudos Eva Perón, San Martín, Provincia de Buenos Aires, Argentina; Bone Marrow Transplantation Unit, Instituto Nacional de Câncer, Rio de Janeiro, RJ, Brazil; Hospital General de Agudos “Dr.Cosme Argerich”, Buenos Aires, Argentina; Molecular Oncology Research Center, Hospital do Câncer de Barretos, Barretos, Brazil; Bone Marrow Transplantation Unit, Instituto Nacional de Câncer, Rio de Janeiro, RJ, Brazil; Department of Pathology, Centro Hospitalario Pereira Rossell, Montevideo, Uruguay; Institut Pasteur de Montevideo, Montevideo, Uruguay; Universidad de Guadalajara, Guadalajara, Mexico; Universidad de Guadalajara, Guadalajara, Mexico; Health Sciences Center, Texas A&M University, Bryan, TX 77807, United States; Laboratorio de Terapia Molecular y Celular, Fundación Instituto Leloir-CONICET, Ciudad de Buenos Aires, Argentina; Fundación para el Progreso de la Medicina, Laboratorio de Investigación en Cáncer, Córdoba, Argentina; CONICET, Córdoba, Argentina; FCEFyN, Depto. de Computación, Escuela de Ingeniería Biomédica, Universidad Nacional de Córdoba, Córdoba, Argentina; Laboratorio de Terapia Molecular y Celular, Fundación Instituto Leloir-CONICET, Ciudad de Buenos Aires, Argentina

**Keywords:** Latin America, breast cancer, prognostic, molecular signatures, risk of recurrence, chemotherapy benefit, real-world evidence

## Abstract

**Background:**

Several guidelines recommend the use of different classifiers to determine the risk of recurrence (ROR) and treatment decisions in patients with HR+HER2− breast cancer. However, data are still lacking for their usefulness in Latin American (LA) patients. Our aim was to evaluate the comparative prognostic and predictive performance of different ROR classifiers in a real-world LA cohort.

**Methods:**

The Molecular Profile of Breast Cancer Study (MPBCS) is an LA case-cohort study with 5-year follow-up. Stages I and II, clinically node-negative HR+HER2− patients (*n* = 340) who received adjuvant hormone therapy and/or chemotherapy, were analyzed. Time-dependent receiver-operator characteristic-area under the curve, univariate and multivariate Cox proportional hazards regression (CPHR) models were used to compare the prognostic performance of several risk biomarkers. Multivariate CPHR with interaction models tested the predictive ability of selected risk classifiers.

**Results:**

Within this cohort, transcriptomic-based classifiers such as the recurrence score (RS), EndoPredict (EP risk and EPClin), and PAM50-risk of recurrence scores (ROR-S and ROR-PC) presented better prognostic performances for node-negative patients (univariate C-index 0.61-0.68, adjusted C-index 0.77-0.80, adjusted hazard ratios [HR] between high and low risk: 4.06-9.97) than the traditional classifiers Ki67 and Nottingham Prognostic Index (univariate C-index 0.53-0.59, adjusted C-index 0.72-0.75, and adjusted HR 1.85-2.54). RS (and to some extent, EndoPredict) also showed predictive capacity for chemotherapy benefit in node-negative patients (interaction *P* = .0200 and .0510, respectively).

**Conclusion:**

In summary, we could prove the clinical validity of most transcriptomic-based risk classifiers and their superiority over clinical and immunohistochemical-based methods in the heterogenous, real-world node-negative HR+HER2− MPBCS cohort.

## Teaser

Guidelines recommend the use of different classifiers to determine the risk of recurrence and treatment decisions in patients with HR+HER2– breast cancer. This article evaluates the comparative prognostic and predictive performance of different classifiers in a real-world cohort of Latin American patients.

**Implications for practice**

The evidence provided in this work showed the superiority of several transcriptomic-based molecular signatures over traditional clinical or Ki67-based classifiers in predicting the risk of recurrence of a real-world Latin American node-negative, hormone-dependent breast cancer cohort. This is the first report that support the prognostic validity of 21-gene recurrence score, the EndoPredict score, and the PAM50-based risk-of-recurrence score as an aid for therapeutic decision in node-negative, hormone-dependent Latin American patients.

## Introduction

Breast cancer is a significant global health concern, and advancements in personalized medicine have led to the development of prognostic and predictive biomarkers that aid in treatment decision-making. In hormone-dependent (HR+HER2−) breast cancer, an appropriate determination of risk of progression is crucial to avoid subjecting low-risk patients to unnecessary chemotherapy,^1^ and conversely, to ensure that appropriate treatment is not skipped in those patients who are likely to benefit from chemotherapy. Genomic tests, which consider gene expression patterns derived from transcriptomic data and use specific algorithms that assign a risk score based on those patterns, have shown varying prognostic (ie, risk of recurrence [ROR]) and/or predictive (ie, chemotherapy benefit) performance levels. Consequently, guidelines have been published to provide evidence-based recommendations for transcriptomic-based methods for breast cancer clinicians.^1-4^

The clinical *utility* of a test refers to a measure of its usefulness in the clinic. Prognostic clinical utility for several commercial biomarkers, such as Prosigna,^5-7^ Oncotype DX,^8-11^ MammaPrint,^12,13^ and EndoPredict Clinical,^14,15^ has been thoroughly demonstrated with various levels of evidence, including randomized trials. As a result, patients with low ROR according to these tests can safely spare adjuvant chemotherapy. In addition, Oncotype DX and EndoPredict Clinical have been approved as predictors of absolute chemotherapy benefit, that is, to determine those who might have a significantly better prognosis if treated.^15-17^

Clinical *utility* is highly dependent on reassuring the clinical *validity* of the test, that is, its ability to actually predict a given clinical outcome. In the case of the mentioned commercial tests, clinical validity is reassured when patients labeled as “low risk” actually have an acceptably low probability of events within 5 or 10 years from surgery. It is well known that factors that are tightly controlled in randomized trials may have an additional impact on prognosis in real-world cohorts and might modify the clinical utility of these tests. For this reason, performance testing of these signatures in retrospective and/or real-world settings has also been used to prove their prognostic utility and superiority to other methods.^18-23^ However, the validation of these prognostic and predictive classifiers with a global perspective, and particularly in Latin American (LA) patients with breast cancer is still lacking. This is particularly worrying as LA healthcare systems have limitations in the quality of care that can provide to their patients with breast cancer.^24^

Demonstrating clinical utility of prognostic and predictive biomarkers in LA is hampered by the lack of local randomized trials. In addition, there is a general absence of high quality, comprehensive data of real-world cohorts, including long-term follow-up for survival analysis. As a consequence of this, clinical decision-making in LA hormone-dependent breast cancer still relies primarily on IHC assays^3,25^ and relevant clinical data (such as age, stage, tumor size, nodal status, grade, comorbidities, and patient preferences). Transcriptomic-based tests are not adequately covered by LA health systems, based on the assumption that they do not have evidence of appropriateness for LA patients with breast cancer. Thus, a comparison of the clinical validity of prognostic biomarkers and their predictive usefulness for determining chemotherapy benefit in the HR+HER2− breast cancer subtype in a real-world, diverse cohort of LA patients with breast cancer is the first step toward the confirmation of the clinical utility of these tests.

In 2011, the Latin American Cancer Research Network (LACRN) launched the Molecular Profile of Breast Cancer Study (MPBCS), a prospective cohort of patients with breast cancer from Argentina, Brazil, Chile, Mexico, and Uruguay.^26^ In this study, a comprehensive dataset including clinical, epidemiological, and molecular data were collected. Follow-up data were collected for 5 years. Its primary objective was to determine the distribution of the molecular profiles of invasive breast cancers. The correlation of immunohistochemically (IHC)-defined and PAM50 subtypes with survival has already been published for this cohort.^27^ The LACRN-MPBCS cohort is a unique resource as it comprises a thorough, longitudinal collection of data from LA countries that are seldom represented in other studies, and that are representative of the public health systems of the region.

The aims of the current work were (1) to retrospectively compare the relative performance and clinical validity of relevant transcriptomic-based prognostic algorithms (in their research-based form) and standard markers within the subset of hormone-dependent, node-negative patients with breast cancer of the MPBCS cohort and (2) to corroborate their validity as predictors of chemotherapy benefit in an LA real-world setting. This was done by comparing the fitness of the differential risk scores to survival models described by real survival data.

## Methods

### Patients

The MPBCS is a case-only, prospective observational study launched in 2011 by LACRN in which 1449 LA clinical stages I-III patients with breast cancer were recruited. Patients with bilateral or inflammatory breast cancer or metastatic disease were excluded. The clinical, pathological, and molecular characteristics of the MPBCS cohort have been described in detail elsewhere.^26-28^ The study protocol, approved by the NCI Ethics Committee and local institutional review boards and ethics committees with active federal-wide assurance for the protection of human subjects in each country, is included in Supplementary Material. This study was registered at ClinicalTrials.gov (identifier: NCT02326857) and conducted in accordance with the Declaration of Helsinki and local regulations. All participants signed an informed consent form before the study procedures. STROBE standards have been used throughout this manuscript.

All clinical operations were done as per standard of care by the surgeon and/or oncologist in charge in the different institutions and registered in clinical report forms. Tumors deemed inoperable were subjected to neoadjuvant chemotherapy (Supplementary Material). After surgery, patients received a standard-of-care adjuvant treatment, including hormonal therapy for a minimum of 5 years if ER+, 1 year of trastuzumab if HER2+, and radiation therapy if indicated. The use of additional chemotherapy was decided by the intervening oncologist using mainly clinical, pathological, and immunohistochemical parameters. Patients were followed for 5 years from the surgery date to evaluate recurrence and survival.

**Figure 1.**
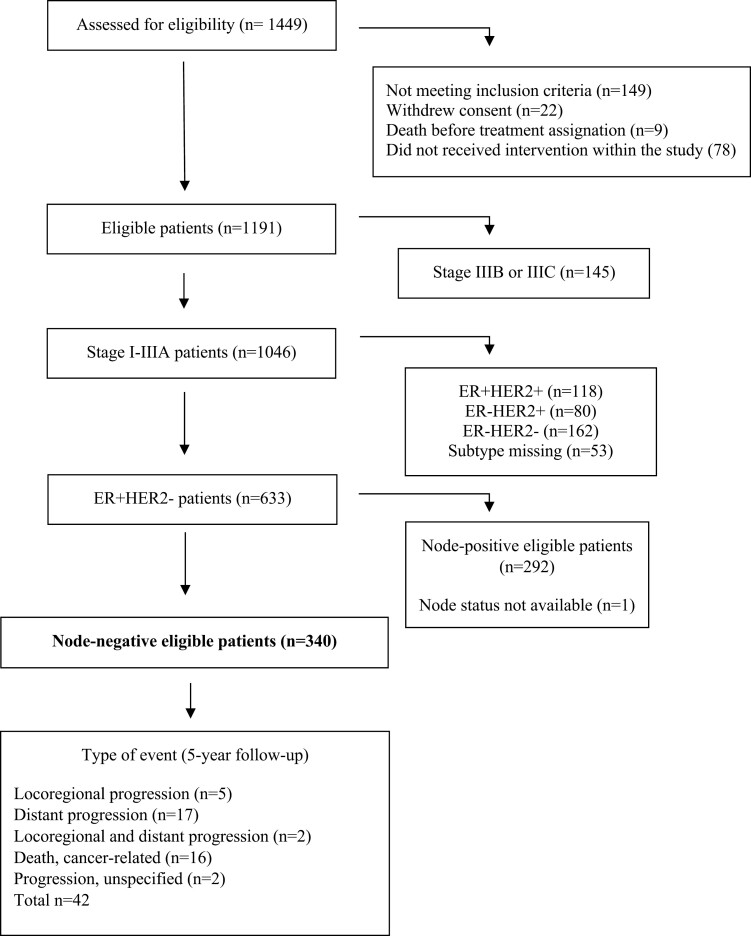
CONSORT flow diagram for the subset of patients with MPBCS analyzed in this work. Events that developed from the date of surgery to the end of the 5-year follow-up are included in the study. Locoregional progression was defined as the compromise of internal, axillary, supra- and intraclavicular nodes, ipsilateral breast, chest wall and/or local-regional skin, and subcutaneous tissue. Distant progression was defined as the compromise of the contralateral breast, distant lymph nodes, distant skin and subcutaneous tissue, pleura, lung, liver, pelvic nodes, bone, bone marrow, and/or brain/CNS.

To perform the retrospective analyses included in this study, we selected those MPBCS patients with clinical node-negative breast cancer according to AJCC 7th Ed with ER and/or PR-positive status (see details about clinical procedures in Supplementary Material). A Consort-style diagram with eligible patients is shown in Figure 1. The final groups for analyses comprised 340 N0 patients who received adjuvant hormonal (H) treatments (aromatase inhibitors, LH-RH agonist, tamoxifen, oophorectomy, or a combination of these) and/or standard-of-care adjuvant chemotherapy based on taxanes, anthracyclines, and cyclophosphamide (TAC). Associations among different clinicopathological variables were tested for significance using the chi-square test.

### Gene signature scores and classification groups

For gene expression analysis, harmonized protocols for 2-color microarray assays were followed^11^ using RNA from snap-frozen biospecimens (either biopsies, from patients of the neoadjuvant arm or surgical specimens, from patients in the primary surgery arm), taken before any treatment. Microarrays were processed in the Agilent gene expression platform (Series C Scanner, Agilent, Santa Clara, CA, USA) installed in central molecular biology laboratories in LACRN countries (Supplementary Material). PCA analysis conducted using the top 30% most variable genes (*n* = 6257) showed no significant bias by study arm (ie, primary surgery or neoadjuvancy), type of sample (ie, core biopsy or surgical specimen), country (ie, Argentina, Brazil, Chile, Mexico, and Uruguay), or year of microarray analysis, on the first and second component.^27^

Gene signature scores and risk assignation for the Genomic Grade Index (GGI),^29^ RS,^16^ GENE70,^30^ and the EP risk score^31^ were calculated using genefu R package functions.^32^ More details about the algorithms and risk thresholds can be found in Supplementary Material. The Nottingham Prognostic Index (NPI)^33^ was uploaded to the MPBCS database by the study clinicians at each center. The PAM50 subtypes were calculated as previously described^27^ (see details in Supplementary Material). ROR-S^5^ and ROR-PC (also called ROR-PT)^34^ were calculated from the PAM50 assignations.

### Classifier performance analysis

The concordance index (C-index) and the cumulative sensitivity and dynamic specificity time-dependent receiver–operator characteristic-area under the curve (C/D tdROC-AUC)^35^ were used to evaluate the time-dependent analytical validity of the prognostic biomarkers (Supplementary Material).

Cohen’s kappa values (*irr* R package) were used to test degree of agreement between each pair of classifiers (Supplementary Material).

### Survival analyses

Invasive disease-free survival (IDFS) analyses were performed using the *survival* R package.^36^ To overcome the problem of immortal bias in observational studies, IDFS was defined as the interval from the date of surgery to the date of the first local or distant recurrence or cancer-related death. The number and type of events registered are described in Figure 1. Patients who remained event free were censored on the date of the last follow-up. The cumulative event probability was evaluated at 5 years.

Univariate and multivariate Cox proportional hazard regression (CPHR) models were used to estimate hazard ratios according to risk class, with the mentioned events as the outcome variable. Details about the selection of variables used for adjustment (ie, menopausal status, clinical tumor stage, use of chemotherapy in neoadjuvancy, and radiotherapy and hormonal adjuvant treatment) are included in Supplementary Material.

To test the predictive value of a continuous score for chemotherapy benefit, a test for interaction was performed using adjusted CPHR models with the chemotherapy (TAC) addition to the adjuvant hormone (H) therapy as treatment (ie, H-TAC vs H as reference) and the prognostic RS, EP risk, EPClin, ROR-S, and ROR-PC scores as continuous variables.

All statistical analyses were 2-sided, and a *P*-value of <.05 was regarded as significant.

## Results

### Description of the MPBCS sub-cohort analyzed in this study

Table 1 summarizes the clinical and pathological information of the selected subgroup of patients of MPBCS included in this work (described in Figure 1). As one of the original secondary outcomes of the MPBC study was to test the response to neoadjuvant therapy, there is a recruitment bias toward locally advanced cases (ie, stages II-III) in this cohort. This is clearly shown by the distribution of tumor histological grades (ie, 78.5% of intermediate-high grades), size (92.9% of tumors larger than 20 mm), and Ki67 expression (ie, 37.4% of tumors with more than 30% of Ki67-positive cells, Table 1). From the total of HR+HER2− tumors that could be classified by the PAM50 algorithm, 65.7% were Luminal A and 24.0% were Luminal B, while 2.9% were classified as HER2-enriched, 1.9% as Basal-like, and 5.4% as Normal-like group. Most HR+HER2− node-negative patients (86.8%) were assigned to primary surgery. In those patients that received neoadjuvant chemotherapy (13.2%) therapeutic response measured as residual cancer burden (RCB) was, in general, poor: 13.0% of HR+HER2− patients in this arm of the study had a complete or almost complete pathological response (RCB 0 to 1) (Table 1).

**Table 1.** Clinical and pathological characteristics of the node-negative HR+HER2− MPBCS patients included in this study.

Clinical characteristics	Node-negative HR+HER2−
	(*N* = 340)
Age range	
<40	7.6% (26)
40-50	24.1% (82)
51-70	50% (170)
>70	18.2% (62)
Menopausal status	
Premenopausal	25.3% (86)
Perimenopausal	0.9% (3)
Postmenopausal	62.9% (214)
Missing	10.9% (37)
Histologic type	
Invasive ductal carcinoma	80.0% (272)
Invasive lobular carcinoma	10.0% (34)
Invasive carcinoma (NOS)	2.1% (7)
Invasive mixed ductal and lobular carcinoma	2.6% (9)
Other	4.7% (16)
Missing	0.6% (2)
Histological grade	
Low	20.3% (69)
Intermediate	49.1% (167)
High	29.4% (100)
Missing	1.2% (4)
cT Stage	
T1 (≤20 mm)	7.1% (24)
T2 (>20-50 mm)	79.4% (270)
T3 (>50 mm)	13.5% (46)
Clinical stage	
Stage I	7.1% (24)
Stage IIA	79.4% (270)
Stage IIB	13.5% (46)
KI67 3-category classification	
<5%	19.1% (65)
5%-30%	43.5% (148)
>30%	37.4% (127)
PAM50 subtype	
LumA	60.3% (205)
LumB	22.1% (75)
HER2E	2.6% (9)
Basal	1.8% (6)
Normal	5% (17)
Missing	8.2% (28)
Arm of study	
Primary surgery	86.8% (295)
Neoadjuvant chemotherapy	13.2% (45)
RCB 0-I	13% (6)
RCB II-III	68.9% (31)
Missing RCB	17.7% (8)
Primary adjuvant treatment	
Hormonotherapy	37.1% (126)
Chemotherapy	5.9% (20)
Hormonotherapy + chemotherapy	55% (187)
Missing	2.1% (7)
Concomitant radiotherapy	
Yes	57.6% (196)
No	40.3% (137)
Missing	2.1% (7)
Type of hormonotherapy^*^	
Tamoxifen	45% (153)
Aromatase inhibitor	20.3% (69)
Aromatase inhibitor + Tamoxifen	14.7% (50)
Other (LH-RH, oophorectomy)^#^	8.5% (29)
Missing	11.5% (39)

^*^Patients were grouped according to their main treatment; some patients in the tamoxifen or aromatase inhibitor categories also received an LH-RH agonist (*n* = 18) or ooforectomy (*n* = 1).

^#^Did not received tamoxifen or aromatase inhibitors; received either LH-RH agonist (*n* = 28) or ooforectomy (*n* = 1).

Abbreviation: RCB, residual cancer burden.

The majority (88.5%) of HR+HER2− patients, regardless of whether they went to primary surgery or neoadjuvancy at the first instance, received some sort of adjuvant hormonotherapy after surgery, from which tamoxifen (alone or in combination with other therapies) was the therapy of choice in 59.7% of the cases (Table 1).

### Assignation of patients to risk classes according to each risk classifier

We retrospectively tested the performance of several risk-of-recurrence classifiers currently recommended in clinical guidelines for stages I-IIIA, HR+HER2− patients in the LACRN-MPBCS cohort. We also included the GGI as a transcriptomic measure of histological grade with prognostic value. We have named the classifiers by their generic name, that is, the one assigned when first described in the literature. A comprehensive description of the variables used for each classifier and their current indication is included in Table 2. Each patient was assigned to an ROR class (low, intermediate, or high), according to the recommendation of each prognostic assay (Table 2).

**Table 2.** Characteristics of the risk assessment methods applied to the LACRN-MPBCS HR+HER2− patients.

Data type	Signature name	Commercial name (if applicable)	Variables involved and risk categories	Application in HR+HER2− patients	Ref.
Clinical data	NPI (Nottingham Prognostic Index)		Tumor size + histological grade + nodal status Low risk: 3-5; intermediate risk: 6-7; high risk: 8-9	Prognosis worsens as the NPI numerical value increases and by using cutoff points patients may be stratified into good, moderate and poor prognostic groups	^33^
Immunohisto-chemistry data	Ki67-A		Percentage of Ki67-positive cells Low risk: ≤20%; high risk: >20%	Ki67 combined with other parameters (ER, PGR and HER2 status) may be used in postmenopausal patients without access to genomic tests to guide adjuvant therapy decisions	^3^
	Ki67-B		Percentage of Ki67-positive cells Low risk: ≤5%; Intermediate risk: 5%-30%; High risk: >30%		^25^
Transcriptomic data	RS (Recurrence score)	Oncotype Dx	21 genes Low risk: ≤25; high risk: ≥26	Use to offer adjuvant chemoendocrine therapy in postmenopausal patients with node-negative (N0) or node-positive (N+) with 1-3 positive nodes and with RS ≥ 26. Node-negative patients ≤ 50 years with RS 16 to 25 may be offered chemoendocrine therapy.	^37^
	EP (EndoPredict) risk		11 genes; low risk: <5; high risk: ≥5	Use to guide adjuvant endocrine and chemotherapy in patients who are postmenopausal or age > 50 years with early-stage ER + and HER2– breast cancer that is node-negative or with 1-3 positive nodes	^31^
	GENE70	MammaPrint	70 genes; low risk: <−0.3; high risk: ≥−0.3	Use to guide adjuvant endocrine and chemotherapy in patients who are postmenopausal or age > 50 years with early-stage ER + and HER2– breast cancer that is node-negative or with 1-3 positive nodes	^30^
	GGI (Genomic Grade Index)		117 genes; low risk: <0; high risk: ≥0	GGI divides classic histologic grade into low and high risk. The ability of GGI to predict response to chemotherapy and separate hormone receptor positive breast cancer subtypes has also been demonstrated	^29^
	ROR-S		PAM50 -50 genes For N0: low: 0-40; intermediate: 41-60; high: 61-100	Use to guide adjuvant endocrine and chemotherapy in postmenopausal patients with node-negative ER + and HER2– breast cancer	^5^
			For N+: low risk: 0-15; intermediate risk: 16-40; high risk: 41-100		
Transcriptomic and clinical data	EPClin	EndoPredict	11 genes + nodal status + tumor size Low risk: <3.3; high risk: ≥3.3	Use to guide adjuvant endocrine and chemotherapy in patients who are postmenopausal or age > 50 years with early-stage ER + and HER2– breast cancer that is node-negative or with 1-3 positive nodes	^31^
	ROR-PC	Prosigna	PAM50 genes + tumor size + proliferation score N0: low risk: 0-40; intermediate risk: 41-60; high risk: 61-100	Use to guide adjuvant endocrine and chemotherapy in postmenopausal patients with node-negative ER + and HER2– breast cancer	^34^
			N+: low risk: 0-15; intermediate risk: 16-40; high risk: 41-100		

Information is provided for each output according to the ASCO Guideline Update 2023^11^ or the literature.

Abbreviations: N0, node-negative tumors; N+, node-positive tumors.

**Figure 2.**
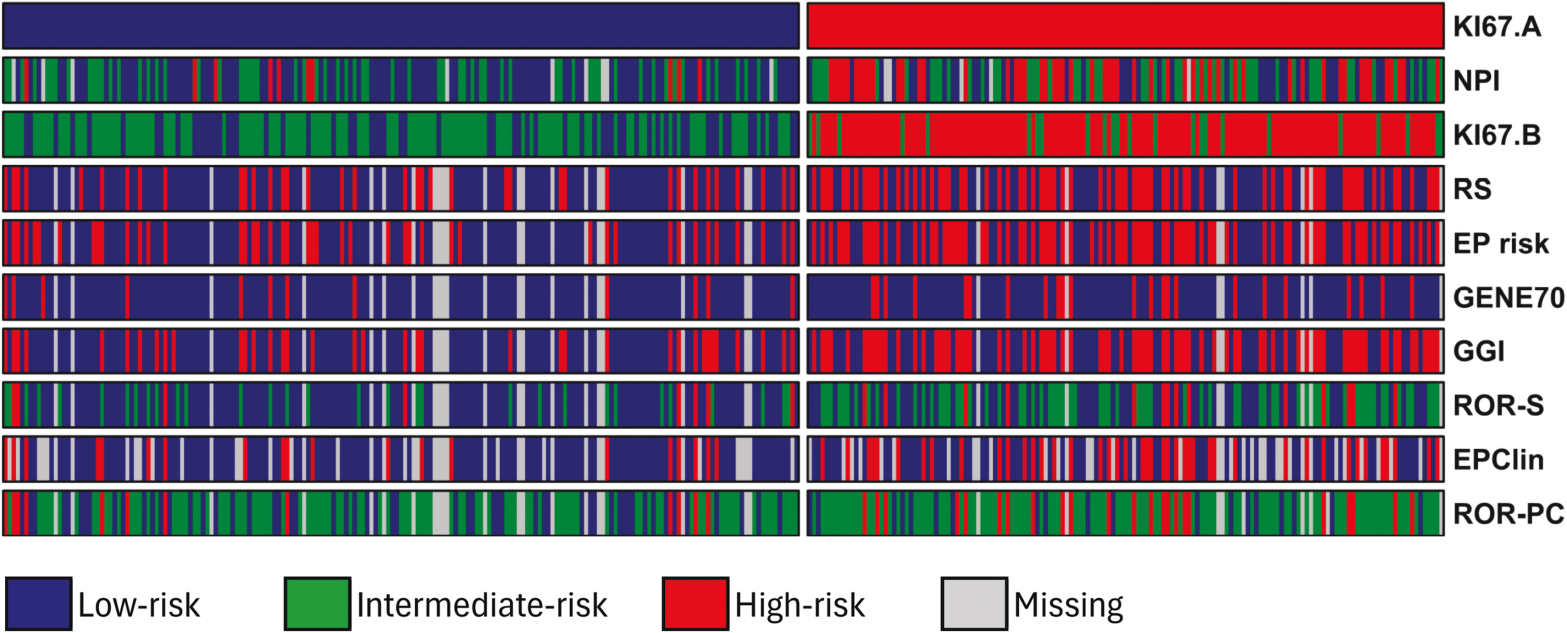
Comparison of risk of recurrence (ROR) according to different methods in the node-negative, HR+HER2− MPBCS cohort. Each patient is represented in columns and the risk classification methods are in rows. Patients were split by Ki67-based stratification (20% cutoff, represented as Ki67-A, top bar) into low- and high-risk groups. Each subsequent row represents the ROR class assigned by each of the different prognostic methods analyzed in this study. See Table 2 for details on the prognostic classifiers.

In the category plot shown in Figure 2, the ROR class according to each classifier is depicted at the individual level. Patients (in the horizontal axis) were split according to the risk category defined by the immunohistochemical-based Ki67 20% threshold (Figure 2, top). This reference biomarker was chosen as it was the most used molecular risk threshold in this set of patients in LA at the time of diagnosis. Some discordance in risk assignation between methods becomes obvious. Most high-risk patients were aggregated in the Ki67-high group. When consistency of risk assignation between classifications was statistically tested, agreement was substantial (Cohen’s kappa > 0.60) for most pairs of transcriptomic-based classifiers; nevertheless, many patients remained in different categories depending on the classifier (Supplementary Figure S1). The agreement of GENE70, the 2 versions of Ki67 classifier, and NPI with the rest of the classifiers was generally poor.

### Evaluation of the prognostic performance of each classifier

Given that the same individual might be classified in different risk classes according to the algorithm used, we then tested which of the classifiers could discriminate best the actual ROR in the HR+HER2− MPBCS cohort using CPHR models. We first quantified how well the CPHR models for each classifier discriminated the 5-year IDFS of MPBCS patients. Higher C-indexes and tdROC-AUC values indicate better discriminating performance. C-indexes of univariate CPHR models of most transcriptomic-based classifiers (except for GENE70 and GGI) were higher than those of Ki67 and NPI (Table 3). As expected, prognostic covariables other than the classifier increased C-indexes of all Cox models, demonstrating an improvement of the model with the addition of independent prognostic factors. RS, EP risk, and EPClin scores also had the highest tdROC-AUC value (0.68) (Table 3).

**Table 3.** Prognostic performance and invasive disease-free survival discrimination by the different classifiers analyzed in node-negative HR+HER2− MPBCS.

Classifier	*N*	C-index	Time-dependent ROC-AUC	Risk group	HR	IC95%	*P*value	Cumulative recurrence probability at 5 years
NPI	321	0.57	0.53	Low			Reference	**12%**
				Intermediate	0.91	(0.42-1.98)	.808	9%
				High	1.54	(0.70-3.38)	.282	21%
NPI (adjusted)	262	0.72		Low			Reference	
				Intermediate	1.19	(0.44-3.23)	.728	
				High	2.54	(1.00-6.45)	*.050*	
KI67-A	335	0.58	0.59	Low			Reference	**10%**
				High	1.78	(0.95-3.34)	.072	17%
KI67-A (adjusted)	271	0.74		Low			Reference	
				High	1.85	(0.85-4.00)	.120	
KI67-B	335	0.60	0.59	Low			Reference	**13%**
				Intermediate	0.84	(0.32-2.20)	.715	9%
				High	1.73	(0.70-4.28)	.238	19%
KI67-B (adjusted)	271	0.75		Low			Reference	
				Intermediate	1.25	(0.34-4.67)	.735	
				High	2.33	(0.67-8.17)	0.185	
RS	307	0.67	0.68	Low			Reference	**7%**
				High	3.23	(1.65-6.33)	*.001*	24%
RS (adjusted)	249	0.78		Low			Reference	
				High	4.06	(1.79-9.19)	*<.001*	
EP risk	307	0.68	0.68	Low			Reference	**6%**
				High	3.64	(1.79-7.40)	*<.001*	23%
EP risk (adjusted)	249	0.80		Low			Reference	
				High	5.48	(2.19-13.7)	*<.001*	
GENE70	307	0.52	0.66	Low			Reference	**12%**
				High	1.34	(0.55-3.27)	.526	13%
GENE70 (adjusted)	249	0.71		Low			Reference	
				High	1.93	(0.68-5.54)	.218	
GGI	307	0.59	0.59	Low			Reference	**8%**
				High	2.71	(1.38-5.31)	*.004*	21%
GGI (adjusted)	249	0.77		Low			Reference	
				High	3.53	(1.55-8.03)	*.002*	
ROR-S	307	0.63	0.63	Low			Reference	**7%**
				Intermediate	3.02	(1.48-6.18)	*.002*	21%
				High	2.88	(0.99-8.39)	.052	20%
ROR-S (adjusted)	249	0.80		Low			Reference	
				Intermediate	5.84	(2.35-14.5)	*<.001*	
				High	9.97	(2.84-35.0)	*<.001*	
EPClin	255	0.68	0.68	Low			Reference	**6%**
				High	2.79	(1.29-6.03)	*.009*	24%
EPClin (adjusted)	205	0.80		Low			Reference	
				High	4.64	(1.75-12.3)	*.002*	
ROR-PC	305	0.61	0.61	Low			Reference	**6%**
				Intermediate	1.66	(0.63-4.37)	.307	13%
				High	3.71	(1.20-11.5)	*.023*	28%
ROR-PC (adjusted)	248	0.77		Low			Reference	
				Intermediate	1.25	(0.39-4.03)	.708	
				High	7.60	(1.99-28.9)	*.003*	

Cox proportional hazard ratios were adjusted by menopausal status, clinical tumor stage (T1, T2, T3, and T4), neoadjuvant therapy (Yes/No), adjuvant radiotherapy (Yes/No), and adjuvant hormone therapy (Yes/No). Statistically significant differences are denoted in italics. The 5-year cumulative recurrence probability for the low-risk group in each classifier is denoted in bold.

Abbreviations: HR: hazard ratio; C-index: concordance index; ROC-AUC: receiver–operator characteristic-area under the curve.

**Figure 3.**
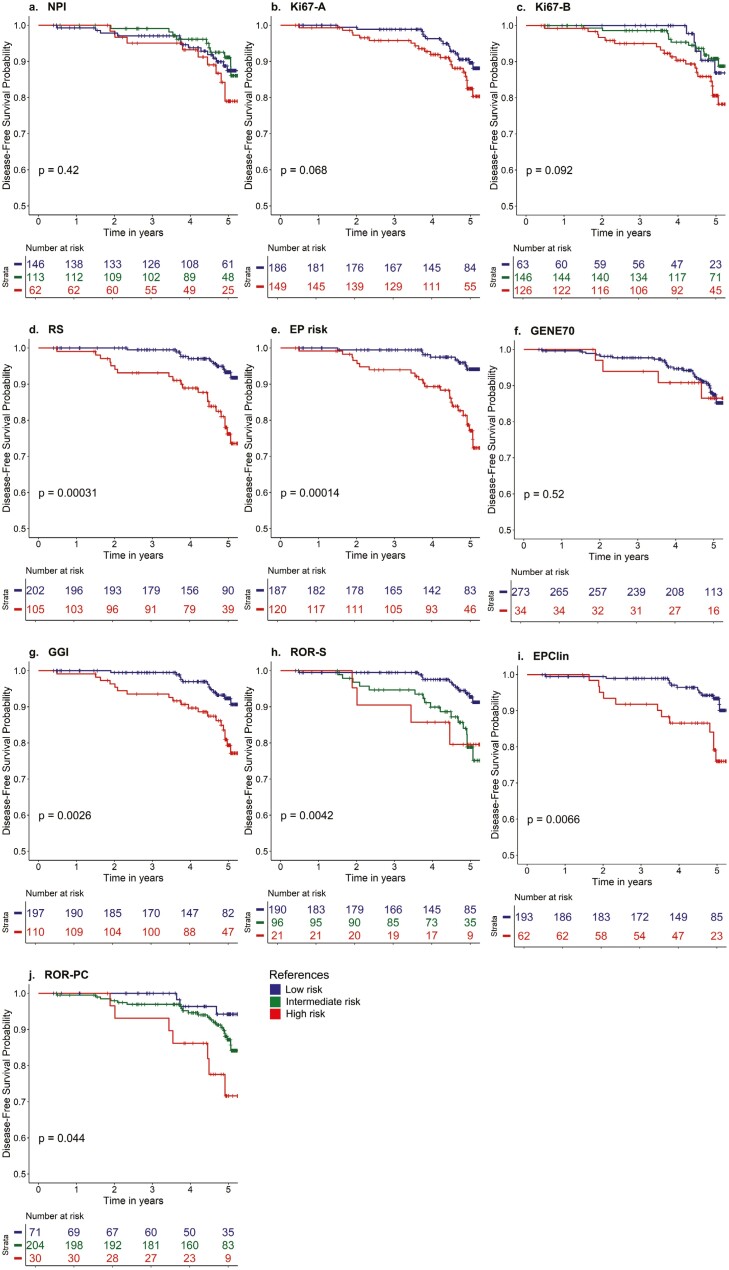
Kaplan-Meier curves of invasive disease-free survival according to risk categories for each of the methods analyzed in node-negative HR+HER2− patients with MPBCS. *Y*-axis has been shortened to allow better discrimination of survival. See Table 2 for details on the prognostic classifiers.

We then analyzed the ability of each risk model to distinguish low risk from intermediate or high risk, using the score thresholds recommended by the literature and summarized in Table 2. Kaplan-Meier curves of 5-year IDFS according to the risk class for each classifier are shown in Figure 3. Only risk classes based on transcriptomic signatures (with the exception of GENE70) showed significant unadjusted differences between low- and high-risk 5-year IDFS (ie, log-rank *P*-value < .05, Figure 3; Table 3). Interestingly, when adjusted hazard ratios were determined using CPHR models, NPI, RS, EP risk, EPClin, GGI, and ROR-S significantly discriminated 5-year IDFS according to risk class (Table 3). The 3-category PAM50-derived ROR-PC significantly discerned low from high risk when adjusted for covariables, failing in significance for the intermediate risk.

When analyzing the cumulative probability of recurrence (ie, how many events arose in 5 years), all transcriptomic-based signatures except for GENE70 showed similar percentages of at-risk individuals in the low-risk category (between 6% and 8%, Table 3). On the other hand, the number of individuals at risk was higher in the low-risk category of NPI and both Ki67 classifiers (10%-13%).

### Analysis of the predictive power for chemotherapy benefit of transcriptomic-based classifiers

It is well known that chemotherapy can be advantageous for disease-free survival in patients with higher risk scores. For testing the predictive power for chemotherapy benefit of the different classifiers in our cohort, we used the scores obtained by each algorithm as a continuous variable (ie, risk score) instead of categorical (ie, risk category) and applied an adjusted CPHR model with interaction. For this analysis we focused on RS, EP, EPClin, GGI, ROR-S, and ROR-PC scores as they are the ones that had the best prognostic performance in our analysis. Only the interaction between RS (as a continuous variable) and treatment (H vs H + TAC) reached statistical significance (*P* = .020, Table 4). In the case of EP risk, the interaction term was very close to significance (*P* = .051, Table 4). From these results we can extrapolate that, at least for RS, some patients with higher scores were showing benefit from adjuvant chemotherapy.

**Table 4.** Interaction between risk scores and adjuvant treatment—hormonotherapy (H) or hormone-chemotherapy (H-TAC) for the RS, EP risk, GGI, ROR-S, EPClin, and ROR-PC classifiers in MPBCS node-negative HR+HER2.

Classifier	Risk group	*P* value	HR	IC95%	*N*
RS	RS	<.001	1.05	(1.03-1.08)	283
	Adj. H-TAC	.041	4.93	(1.07-22.7)	
	RS*Adjuvant (ref. H)	*.020*	0.96	(0.93-0.99)	
EP risk	EP risk	<.001	1.45	(1.18-1.77)	283
	Adj. H-TAC	.055	6.54	(0.96-44.4)	
	EP*Adjuvant (ref. H)	**.051**	0.78	(0.62-1.00)	
GGI	EP risk	.002	1.02	(1.02-1.11)	283
	Adj. H-TAC	.393	1.63	(0.53-5.01)	
	EP*Adjuvant (ref. H)	.155	0.96	(0.91-1.01)	
ROR-S	ROR-S	<.001	1.06	(1.03-1.10)	283
	Adj. H-TAC	.101	4.11	(0.76-22.2)	
	ROR-S*Adjuvant (ref. H)	.112	1.00	(0.93-1.01)	
EPClin	EPClin	.002	2.95	(1.51-5.75)	235
	Adj. H-TAC	.107	14.60	(0.56-381)	
	EPClin*Adjuvant (ref. H)	.094	0.47	(0.19-1.37)	
ROR-PC	ROR-PC	.002	0.96	(0.93-0.99)	282
	Adj. H-TAC	.110	5.37	(0.68-42.2)	
	ROR-PC*Adjuvant (ref. H)	.147	0.97	(0.94-1.01)	

CHPR models were adjusted by age (continuous), menopausal status (pre/post/perimenopausal), country (Argentina, Brazil, Chile, Mexico, and Uruguay), clinical tumor status (T1, T2, T3, T4), use of chemotherapy in neoadjuvancy (Yes/No), use of radiotherapy in adjuvancy (Yes/No), and type of adjuvant endocrine treatment (treatment as described in Table 1). The number in italic corresponds to a statistically significant value for the interaction; the number in bold denotes a marginal statistical significance for the interaction.

## Discussion

Since seminal works described the first gene expression-based signatures with prognostic value in breast cancer,^30,31,37,38^ many molecular signatures have been developed to classify patients with breast cancer according to their ROR. Although not all available signatures currently have the highest level of evidence as a predictive biomarker, the risk assignation offered by these tests is usually considered for chemotherapy guidance in this subtype of breast cancer. However, to the best of our knowledge, no indication of their usefulness in LA patients with breast cancer has been provided before. Although practice changes using Oncotype DX for management of early-stage LA patients with breast cancer were recently proved,^39^ its impact on survival could not be studied due to the lack of follow-up data.

In this study, we explored the prognostic performance of 7 different transcriptomic-based classifiers in a real-world, node-negative HR+HER2− LA cohort, and compared it to the clinical or immunohistochemical-based prognostic biomarkers available in LA, such as NPI and Ki67. In general, the transcriptomic-based signatures showed good performance at calling the ROR for the subset of node-negative HR+HER2− patients of the MPBCS cohort. The analysis of C-indices and tdROC-AUC, which measures the level of adjustment of low and high risk scores to real survival data, showed values close to those published in the literature^19^ for RS, EP risk, EPClin, ROR-S, and ROR-PC and comparatively better than the ones achieved by NPI and Ki67 in this same cohort. GENE70 has good discriminative performance according to the C-index and tdROC-AUC but its overall poor performance made them not suitable for prognosis in its research form. The research-based algorithm used for calculation of risk by GENE70 used 56 of the 70 genes in the signature, which are the ones available in the Agilent platform used in this work,^32^ and the *genefu* cutoff values used to define risk are different from those used in the commercial version of this algorithm, being this a possible explanation for the poor performance of this classifier. In that sense, it has been argued that research-based signatures may not exactly mimic commercial final versions validated with level I evidence^40^; however, the performance of several of the research-based classifiers tested in this work has been close to that of their commercial versions and they have been widely used in the literature.^41-43^

RS, both EP signatures, GGI, ROR-PC, and ROR-S left 8% or lower probability of 5-year recurrence in low-risk node-negative patients, which is acceptable (ie, <10%) although slightly higher than those published in other cohorts.^19,44^ This is reasonable to expect due to (1) a recruitment bias toward more advanced cases and (2) the fact that the MPBCS is an LA public health-based cohort. Public health systems in Latin America are still struggling to keep up with oncologic quality care, including access to standard-of-care treatment.^24,45^ In any case, residual risks obtained for transcriptomic-based risk models were lower than those attainable with NPI or Ki67 risk scores. Accurate determination of residual risk in patients treated with hormonal therapy might be used to safely spare the toxicity of chemotherapy in low-risk patients.^8,14,15^ Our evidence suggests that the addition of a transcriptomic-based signature to other prognostic factors might have allowed a more precise risk evaluation and would have helped minimize over-treatment in this group of patients, given that more than 60% of the patients of this node-negative, early breast cancer cohort received adjuvant chemotherapy.

For RS (and to some extent for EP risk), we were able to prove their predictive capacity for reporting chemotherapy benefit in node-negative patients. However, endorsing the exact residual risk or the absolute benefit of chemotherapy claimed by commercial tests would require a stronger level of evidence that we cannot attain with the relatively low number of patients of our retrospective, real-world analysis. From a conservative point of view, we consider that caution must be used when interpreting the percentage of absolute benefit of chemotherapy that is informed by commercial test providers, such as EndoPredict or OncotypeDX, as they may not accurately reflect the real risk in LA populations.

Our study has limitations. The most important one is the heterogenity of this real-world cohort that does not adjust precisely to the recommendations for the use of the prognostic tests. Due to the cohort’s relatively small size and its inevitable bias toward advanced cases, we decided to include, in a global analysis, all node-negative patients even if they did not adjust to recommendations (ie, premenopausal or neoadjuvant-treated patients). In order to statistically control this heterogeneity, we took into account those variables as confounders in survival models. The fact that transcriptomic-based, adjusted risk models resulted in consistent, significantly better prognostic performance than clinical or IHC-based prognostic methods, even among this diverse collection of cases, indicates the prognostic superiority of most transcriptomic-based classifiers. Nevertheless, these comparisons are valid under the assumption that all significant confounders, either tested or not, affect all models equally.

Regarding the predictive power of the tested classifiers, both RS and EPClin have demonstrated to be predictors of chemotherapy benefit. Although we were able to validate the significant interaction between RS and the addition of chemotherapy, we did not reach significance for EPclin. We may hypothesize that the already described heterogeneity of the cohort may be responsible for the marginal significance obtained with this classifier.

Another limitation of the study is that the validation of risk classifiers was performed over a 5-year period, which is the period in which most recurrences occur. Ongoing efforts are collecting 10-year follow-up data to extend these observations. Finally, we left aside the analysis of node-positive patients as the combination of the small number of patients and their clinical heterogeneity played against consistency in the prognostic power of the studied signatures.

## Conclusion

In summary, according to the adjustment of risk models to survival data, the proportion of patients that fall in the low-risk category, the statistical significance of the hazard ratios between low- and high-risk groups and the acceptable percentage of recurrence seen in the low-risk category, we have proved the clinical validity of several transcriptomic-based signatures and their superiority to classical risk stratifiers in a LA real-world breast cancer cohort. This study was neither designed to validate the absolute performance (ie, clinical utility) of commercial tests in LA nor to give a recommendation for any commercial test. However, we believe that this retrospective analysis might be used as positive evidence for considering the use of several gene expression signatures in the clinical decision-making process for node-negative LA patients with breast cancer.

## Supplementary material

Supplementary material is available at *The Oncologist* online.

oyae191_suppl_Supplementary_Material

## Data Availability

The data that support the findings of this study are available upon reasonable request and with permission of the LACRN Steering Committee.
