# Supplementary material for: Closing the gap: prognostic and predictive biomarker validation for personalized care in a Latin American hormone-dependent breast cancer cohort
Source: Oncologist. 2024 Aug 8;29(12):e1701–13. doi: 10.1093/oncolo/oyae191 (PMC11630759; doi:10.1093/oncolo/oyae191)
Supplement: oyae191_suppl_Supplementary_Material [file oyae191_suppl_supplementary_material.pdf]

Closing the Gap: Prognostic and Predictive Biomarker Validation for Personalized Care in a Latin American Hormone-Dependent Breast Cancer Cohort.

Daniela Alves da Quinta, BSc[1,2], Darío Rocha, BSc[3], Javier Retamales, MD[4], Diego Giunta, MD, PhD[5], Nora Artagaveytia, PhD[6], Carlos Velazquez, PhD[7], Adrian Daneri MD, PhD[8], Bettina Müller, MD[9], Eliana Abdelhay, PhD[10], Alicia I. Bravo, MD[11], Mónica Castro, MD[12], Cristina Rosales, MD[13], Elsa Alcoba, MD[13], Gabriela Acosta Haab, MD[13], Fernando Carrizo, MD[11], Irene Sorin, MD[10], Alejandro Di Sibio, MD[14], Márcia Marques Silveira, PhD[15], Renata Binato, PhD[10], Benedicta Caserta, MD[16], Gonzalo Greif, PhD[17], Alicia Del Toro-Arreola, PhD[8], Antonio Quintero Ramos, PhD[8], Jorge Gómez[18], MD, PhD, Osvaldo L. Podhajcer, PhD[1], Elmer A. Fernández, PhD[19,20,21], LACRN investigators and Andrea S. Llera, PhD[1]

SUPPLEMENTARY MATERIALS

Table of contents

- Supplementary Methods p.1
- Supplementary Figure 1 p.5
- MPBC Study Protocol p.6

SUPPLEMENTARY METHODS

Clinical procedures

Women with clinical stage II or III (AJCC 7) breast cancer were eligible for this study and were assigned to one of two primary treatments according to breast surgeon criteria: the Primary Surgery arm for women with an indication for primary surgery, and the Neoadjuvant arm for women eligible for neoadjuvant chemotherapy (NAC). Patients that were reclassified as stage I after recruitment were kept on study. Patients with bilateral or inflammatory breast cancer or metastatic disease were excluded. The participants underwent routine clinical evaluation and staging, including abdominal and thoracic computed tomography (CT), and bone scans. Snap-frozen and formalin-fixed paraffin-embedded (FFPE) tissue samples were collected during surgery in the Primary Surgery arm, during diagnostic core-biopsy procedure (before NAC) and at the time of surgery for the Neoadjuvant arm. Tissues were processed according to TCGA-based harmonized standardized operative procedures (SOPs)[9]. Snap-frozen tissue samples were stored at -150°C and FFPE samples were stored at controlled room temperature. The tumor location was marked before NAC following standardized procedures.

The recommended regimens for NAC included doxorubicin 60 mg/m2 IV or epirubicin 75-100 mg/m2 IV + cyclophosphamide 600 mg/m2 IV every 3 weeks for 4 cycles, followed by weekly paclitaxel 80 mg/m2 IV for 12 weeks or docetaxel 70-90 mg/m2 IV every 3 weeks for 4 cycles. Addition of trastuzumab to the taxane regimen for patients with HER2+ tumors was strongly recommended.

The participants underwent surgery, either total mastectomy or quadrantectomy (BCS), within 42 days of the completion of chemotherapy. Standard postoperative treatments were recommended. The participants were followed-up for 5-years after the date of surgery for survival and recurrence. Clinical data were captured using electronic case report forms in OpenClinica®, and accuracy was ensured by local data managers and data reviewers.

Immunohistochemistry and microarray analyses

Assessment of expression of tumor ER (ER pharmDx), PR (PR pharmDx), HER2 (HercepTest), and Ki67 (FLEX Monoclonal Mouse Anti-Human Ki-67 Antigen, Clone MIB-1) was done according to established procedures and complemented by HER2 fluorescent in situ hybridization - FISH (HER2 FISH pharmDx) or chromogenic in situ hybridization - CISH (Her2 DuoCISH pharmDx) as appropriate (for HER2 IHC 2+ cases). ER and PR were considered positive when 1% or more of the tumoral cells showed positive nuclear staining, irrespectively of intensity. Reference centers in all countries went through an external evaluation by the College of American Pathologists for IHC (ER, PR), and FISH and CISH HER2 testing. In addition, a specific analysis of the performance of the Ki67 determination was done to evaluate variation between centers.

Total RNA isolation, quantification, labeling, hybridization, and scanning were done following SOPs to ensure proper harmonization and minimize bias. Batch correction of a small country bias in the third PCA component was done as described [1]. To perform PAM50-subtyping, the expression log2 ratios of the PAM50 genes were centered according to their balanced medians. A total of 248 ER-negative and 248 ER-positive samples were selected to determine an ER-balanced median for each of the 50 genes used in the PAM50 subtyping scheme. This included pairs from each country (Argentina 59, Brazil 46, Chile 32, Mexico Guadalajara 73, Mexico Sonora 22, and Uruguay 16 pairs). The balanced medians were then used to center the gene expression data of the whole cohort and the PAM50 algorithm was run as suggested [2] (generic code - https://genome.unc.edu/pubsup/breastGEO/PAM50.zip).

Score calculation and stratification of risk groups

We calculated risk scores for PAM50-derived risk of recurrence (ROR), which forms the basis of the Prosigna assay [2]; the recurrence score (RS) used in the commercial test Oncotype DX [3]; the GENE70 algorithm used by MammaPrint [4]; and EP risk, which constitutes the basis for Endopredict [5]. Some of these algorithms have been combined with clinical information for commercial versions. Prosigna (ROR-PC) and EndoPredict Clinical (EPClin) consider, in addition to transcriptomic data, tumor size [6] or, tumor size and nodal status [5], respectively. The EPclin score was calculated using the formula $0.35 \cdot t + 0.64 \cdot n + 0.28 \cdot s$, where t is the tumor size, n is the nodal size, and s is the EP risk score[5]. The KI67 risk was calculated in two different ways according to the evolution of the recommendations for this marker. For Ki67, in the 2-category risk (Ki67-A), patients with > 20% of Ki67-

positive cells were classified as high risk and ≤ 20% of Ki67-positive cells as low risk[1], and for the 3-category (ki67-B), < 5%, 5%-30% and > 30% of Ki67-positive cells were assigned as low, intermediate and high risk, respectively [7].When stratifying risk according to RS scores, node-negative patients with RS > 25 were classified as high risk, and RS ≤ 25 as low risk). Patients with an EPclin score < 3.3 were classified as low risk and and the rest (≥ 3.3) were categorized as high risk.

Cox proportional hazards regression models

For Cox proportional hazards regression model, the proportional hazards assumption was checked using the statistical test based on the scaled Schoenfeld residuals. All HRs are displayed with their 95% confidence intervals (CI).

When multivariate Cox proportional hazards regression models were performed, prognostic covariables used for model adjustment were menopausal status, clinical tumor stage (cT), use of chemotherapy in neoadjuvancy (Yes/No), use of adjuvant radiotherapy (Yes/No), and use of adjuvant hormone therapy (Yes/No). The choice of covariables was driven both by previous knowledge of the prognostic impact of each variable, and the kind of variables available in the MPBCS cohort. PAM50 subtyping was not used as a covariable as all signatures are highly correlated with subtypes. For the analysis of the interaction between risk scores and the addition of chemotherapy, we included age (as continuous) and country as additional adjustment variables.

Performance statistics

The agreement between tests (i.e. the percentage of patients that are classified in the same risk class by two different prognostic tests) was assessed by Kappa statistics interpreted as follows: values ≤ 0 as no agreement, 0.01–0.20 as none to slight, 0.21–0.40 as fair, 0.41– 0.60 as moderate, 0.61–0.80 as substantial, and 0.81–1.00 as almost perfect agreement [8].

Prognostic and predictive biomarkers are commonly measured at a single time point (i.e., at diagnosis) and then are used to guide decisions for subsequent time points. However, their performance may vary over time because of the patient's clinical conditions and treatment. To take this issue into account, we calculate the C/D tdROC-AUC (*smoothROCtime* R package) [9] and the C-index (*survcomp* R package)[10], which are used to test how well a prognostic marker discriminate risks [11]. Both statistics were calculated using the risk continuous values. The output of the analysis render scores between 0.5 and 1, where 1 corresponds to a complete model prediction, and 0.5 represents a random prediction.

Data management

Plots were created using the *ggplot2* [12] and *ComplexHeatmap* [13, 14] R packages. All analyses were performed using R version 4.1.3. For all statistical comparisons we considered significant p-values as ≤0.05.

References

1. Llera AS, Abdelhay ESFW, Artagaveytia N, et al: The Transcriptomic Portrait of Locally Advanced Breast Cancer and Its Prognostic Value in a Multi-Country Cohort of Latin American Patients [Internet]. Front Oncol 12, 2022Available from: https://www.frontiersin.org/article/10.3389/fonc.2022.835626

2. Parker JS, Mullins M, Cheang M, et al: Supervised Risk Predictor of Breast Cancer Based on Intrinsic Subtypes. J Clin Oncol 27:1160–1167, 2009

3. Paik S, Tang G, Shak S, et al: Gene Expression and Benefit of Chemotherapy in Women With Node-Negative, Estrogen Receptor–Positive Breast Cancer. J Clin Oncol 24:3726–3734, 2006

4. Veer LJ van 't, Dai H, Vijver MJ van de, et al: Gene expression profiling predicts clinical outcome of breast cancer. Nature 415:530–536, 2002

5. Filipits M, Rudas M, Jakesz R, et al: A New Molecular Predictor of Distant Recurrence in ER-Positive, HER2-Negative Breast Cancer Adds Independent Information to Conventional Clinical Risk Factors. Clin Cancer Res 17:6012–6020, 2011

6. Nielsen TO, Parker JS, Leung S, et al: A Comparison of PAM50 Intrinsic Subtyping with Immunohistochemistry and Clinical Prognostic Factors in Tamoxifen-Treated Estrogen Receptor–Positive Breast Cancer. Clin Cancer Res 16:5222–5232, 2010

7. Nielsen TO, Leung SCY, Rimm DL, et al: Assessment of Ki67 in Breast Cancer: Updated Recommendations From the International Ki67 in Breast Cancer Working Group. J Natl Cancer Inst 113:808–819, 2021

8. Gamer M, Lemon J, Fellows I, et al: Various Coefficients of Interrater Reliability and Agreement [Internet], 2019Available from: https://cran.r-project.org/web/packages/irr/index.html

9. Díaz-Coto S, Martínez-Camblor P, Pérez-Fernández S: smoothROCtime: an R package for time-dependent ROC curve estimation. Comput Stat 35:1231–1251, 2020

10. Schröder MS, Culhane AC, Quackenbush J, et al: survcomp: an R/Bioconductor package for performance assessment and comparison of survival models. Bioinformatics 27:3206–3208, 2011

11. Haibe-Kains B, Desmedt C, Sotiriou C, et al: A comparative study of survival models for breast cancer prognostication based on microarray data: does a single gene beat them all? Bioinformatics 24:2200–2208, 2008

12. Wickham H: Programming with ggplot2, in Wickham H (ed): ggplot2: Elegant Graphics for Data Analysis. Cham, Springer International Publishing, 2016, pp 241–253

13. Gu Z, Eils R, Schlesner M: Complex heatmaps reveal patterns and correlations in multidimensional genomic data. Bioinformatics 32:2847–2849, 2016

14. Gu Z: Complex heatmap visualization [Internet]. iMeta , 2022Available from: https://onlinelibrary.wiley.com/doi/10.1002/imt2.43

	NPI*	Ki67-B*	Ki67-A	RS	EP risk	GENE70	GGI	ROR-S*	EPClin	ROR-PC*
**NPI***	1									
**Ki67-B***	0.41	1								
**Ki67-A**	0.49	1	1							
**RS**	0.39	0.29	0.29	1						
**EP risk**	0.45	0.36	0.30	0.71	1					
**GENE70**	0.19	0.08	0.09	0.33	0.26	1				
**GGI**	0.33	0.32	0.32	0.74	0.63	0.33	1			
**ROR-S***	0.27	0.15	0.15	0.64	0.48	0.63	0.60	1		
**EPClin**	0.48	0.25	0.25	0.57	0.65	0.31	0.55	0.62	1	
**ROR-PC***	0.63	0.51	0.38	0.90	0.84	0.58	0.92	1	0.80	1

Cohen's Kappa values

- ≤ 0 : no agreement
- 0.01–0.20 : none to slight
- 0.21–0.40 : fair
- 0.41– 0.60 : moderate
- 0.61–0.80 : substantial
- 0.81–1.00 : almost perfect

Supplementary Figure 1. Agreement between the prognostic classifiers in the risk category calling for node-negative HR+HER2- LACRN-MPBCS patients. The number inside the boxes are the specific Cohen's kappa value for each comparison. The light kappa for all classifiers altogether was 0.81.

* Correspond to 3-category classifiers. For those, only low- and high-risk categories were compared.

United States-Latin America Cancer Research Network (US-LA CRN)

Molecular Profiling of Stage II and III Breast Cancer in Latin American Women Receiving Standard-of-Care Treatment

National Coordinators:

ARGENTINA
Osvaldo Podhajcer, PhD
Instituto Leloir
Av. Patricias Argentinas 435
Capital Federal, Buenos Aires, Argentina

BRAZIL
Luiz Antonio Santini Rodrigues da Silva, MD
Instituto Nacional de Cáncer
Pc. da Cruz Vermelha, 23
Río de Janeiro, Brazil

CHILE
Bettina Müller, MD
Instituto Nacional del Cáncer
Profesor Zañartu 1010, Independencia
Santiago, Chile

MEXICO
Adrián Daneri Navarro, PhD
Universidad de Guadalajara
Avenida Juárez 976
Guadalajara, Jalisco, México

Carlos Arturo Velázquez Contreras, PhD
Universidad de Sonora
Boulevard Luis Encinas y Rosales S.N. Centro
Hermosillo, Sonora, México

URUGUAY
Graciela Sabini, MD
Programa Nacional de Control del Cáncer (PRONACCAN)
Ministerio de Salud Pública
Av. 18 de Julio 2175
Montevideo, Uruguay

UNITED STATES
Thomas Gross, MD, PhD
National Cancer Institute
9609 Medical Center Drive
Rockville, Maryland 20850, USA

TABLE OF CONTENTS

LIST OF ABBREVIATIONS 3
SCHEMA FIGURE A 5
SCHEMA FIGURE B 6
6
1 OBJECTIVES 7
1.1 Primary Objective 7
1.2 Secondary Objectives 7
2 BACKGROUND AND RATIONALE 7
2.1 Breast Cancer 7
2.2 Breast Cancer Burden in Latin America 8
2.3 Molecular Profiling and Genetic Analysis of Breast Cancer 9
2.4 Neoadjuvant Chemotherapy and Evaluation of Response 10
3 SUMMARY OF STUDY PLAN 11
4 INCLUSION AND EXCLUSION CRITERIA 13
4.1 Overview 13
4.2 Inclusion and Exclusion Criteria for Part A of the Study: 13
4.3 Inclusion and Exclusion Criteria for Part B of the Study: 14
4.4 Inclusion of Women and Minorities 15
4.5 Recruitment and Retention Plan 15
5 BIOSPECIMENS FOR BIOMARKER, GENETIC, AND GENOMIC ANALYSES 15
5.1 Part A of the Study: Gene Expression Profiling by PAM-50 Assay 15
5.2 Part B of the Study: Neoadjuvant Chemotherapy 17
5.3 Biospecimens Collection, Handling, Processing, Labeling, and Storage 17
5.4 BSI-II^{TM} 18
6 CRITERIA FOR EVALUATION AND ENDPOINT DEFINITION 18
6.1 Primary Endpoint 18
6.2 Secondary Endpoints 18
6.3 Target Lesion 18
6.4 Clinical Assessments 19
6.5 Off-Study Criteria 19
6.6 Study Termination 19
7 CHEMOTHERAPY ADMINISTRATION 19
7.1 Neoadjuvant Chemotherapy Administration 19
7.2 Adjuvant Chemotherapy Administration 20
8 CLINICAL AND RESEARCH EVALUATIONS AND PROCEDURES 21
8.1 Schedule of Events Table* 21
8.2 Enrollment/Baseline 23
8.3 Baseline Surgical Evaluation 23
8.4 Neoadjuvant Chemotherapy 24
8.5 Pre-surgery Evaluation after Neoadjuvant Chemotherapy 24
8.6 Surgery 24
8.7 Post-treatment Surgical Specimen Submission 25
8.8 Follow-up Period 25
9 STATISTICAL CONSIDERATIONS 25
9.1 Study Design/Objectives 25
9.2 Sample Size/Accrual Rate 26

9.3 Randomization and Stratification ... 27
9.4 Primary Endpoint(s) ... 27
9.5 Secondary Endpoint(s) ... 27
9.6 Reporting and Exclusions ... 27
9.7 Evaluation of Toxicity ... 27
9.8 Evaluation of Response to Standard Neoadjuvant Chemotherapy ... 27
9.9 Missing Data ... 27
9.10 Interim Analysis ... 27
9.11 Ancillary Studies ... 27
10 STUDY MONITORING ... 28
10.1 Data Management ... 28
10.2 Case Report Forms ... 28
10.3 Source Documents ... 28
10.4 US–LA CRN Monitoring ... 28
10.5 Record Retention ... 29
10.6 Clinical Trials Agreement ... 29
11 ETHICAL AND REGULATORY CONSIDERATIONS ... 29
11.1 Institutional Review Board/Ethics Committee Approval ... 29
11.2 Informed Consent ... 29
11.3 Data Confidentiality Safeguards ... 30
11.4 Submission of Regulatory Documents ... 30
11.5 The Role of the US NIH/NCI ... 30
REFERENCES ... 32
APPENDIX A: Performance Status Criteria ... 39
APPENDIX B: List of Changes for Protocol Version 3.4.2 (September 4, 2014) ... 40
APPENDIX C: MPBC Study Epidemiology Questionnaire ... 46

LIST OF ABBREVIATIONS

AC	Doxorubicin, cyclophosphamide
AJCC	American Joint Committee on Cancer
ALP	Alkaline phosphatase
ALT	Alanine aminotransferase
AST	Aspartate aminotransferase
BRCA1	Breast cancer susceptibility gene 1
BRCA2	Breast cancer susceptibility gene 2
CBC	Complete blood count
CDMS	Clinical Data Management System
CDE	Common data element
CGH	Center for Global Health
CISH	Chromogenic *in situ* hybridization
CMF	Cyclophosphamide, methotrexate, fluorouracil
CRF	Case report form
CT	Computed tomography
DCIS	Ductal carcinoma *in situ*
DFS	Disease-free survival
DMC	Data Monitoring Committee
EC	Epirubicin, cyclophosphamide
ECHO	Echocardiogram
ECOG	Eastern Cooperative Oncology Group
eCRF	Electronic case report form
EDC	Electronic data capture
EORTC	European Organization of Research and Treatment of Cancer
ER	Estrogen receptor
FAC	Fluorouracil, doxorubicin, cyclophosphamide
FEC	Fluorouracil, epirubicin, cyclophosphamide
FFPE	Formalin-fixed/paraffin-embedded
FISH	Fluorescent *in situ* hybridization
GCP	Good Clinical Practice
H&E	Hematoxylin and eosin
HCG	Human chorionic gonadotropin
HER2	Human epidermal growth factor receptor 2
HIPAA	Health Insurance Portability and Accountability Act
HR	Hormone receptor
ICH	International Conference on Harmonization
IHC	Immunohistochemistry
IRB	Institutional Review Board
LABC	Locally advanced breast cancer
LAC	Latin America and the Caribbean
LVEF	Left ventricular ejection fraction
MDACC	MD Anderson Cancer Center
MOP	Manual of Operations
MUGA	Multigate acquisition scanning
NCI	National Cancer Institute

NSABP	National Surgical Adjuvant Breast and Bowel Project
OHRP	Office of Human Research Protections
OS	Overall survival
pCR	Pathologic complete response
PgR	Progesterone receptor
PT	Preferred term
RCB	Residual cancer burden
RFS	Relapse-free survival
SGOT	Serum glutamic-oxaloacetic transaminase
SGPT	Serum glutamic-pyruvic transaminase
SOP	Standard operating procedure
SNP	Single nucleotide polymorphism
TFF	Time to first failure
ULN	Upper limits of normal
US–LA CRN	United States–Latin America Cancer Research Network

SCHEMA FIGURE A

Figure A: Breast Cancer Study—Part A: Characteristics by Immunohistochemistry: Microarray PAM50 Assay

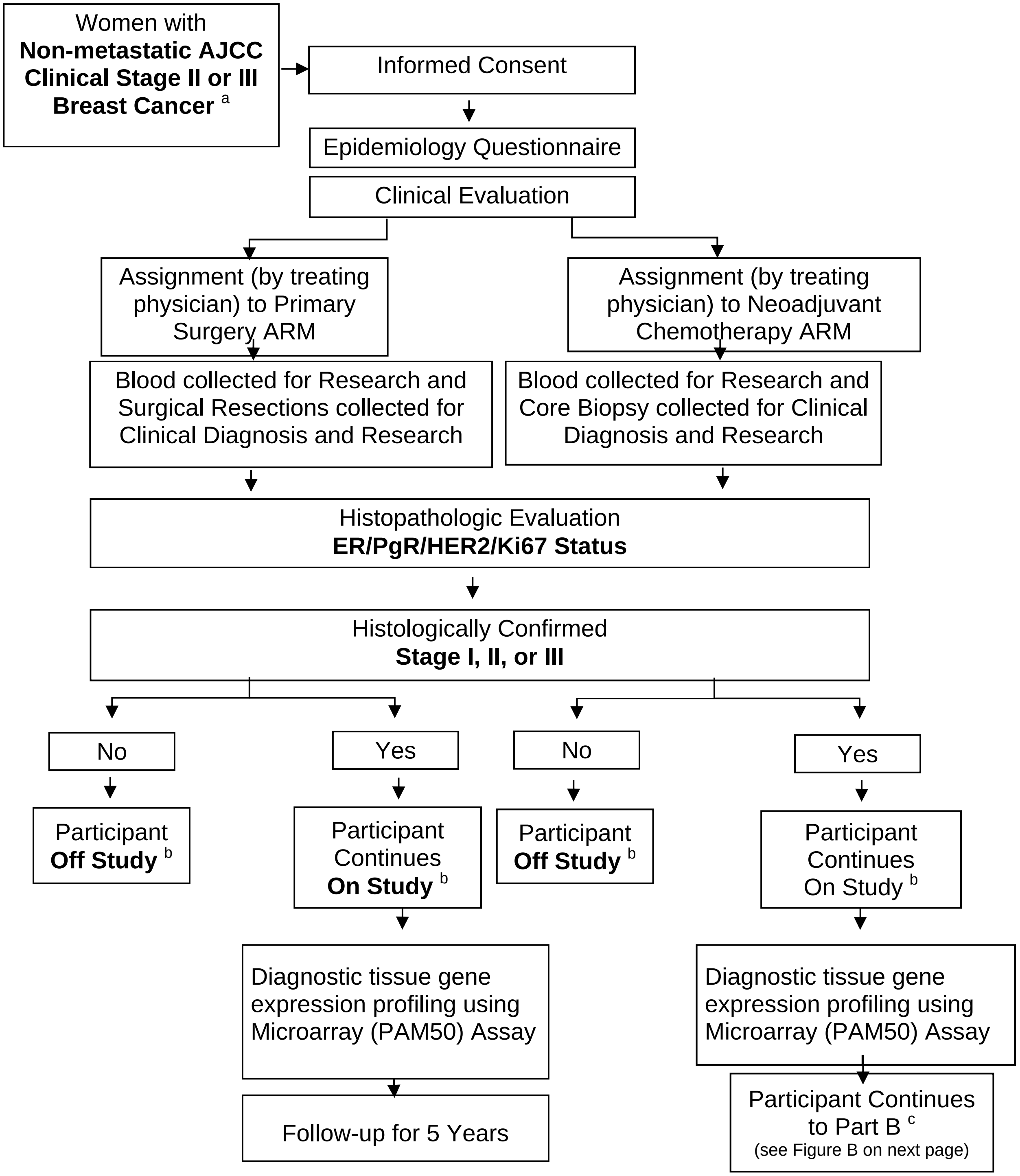

[a] Participant must also meet other inclusion and exclusion criteria listed in section 4.2.
[b] Both participants on and off study continue receiving local institutional standard-of-care treatment.
[c] Participants must also meet other inclusion and exclusion criteria listed in section 4.3.

SCHEMA FIGURE B

Figure B: Breast Cancer Study—Part B: Neoadjuvant Chemotherapy

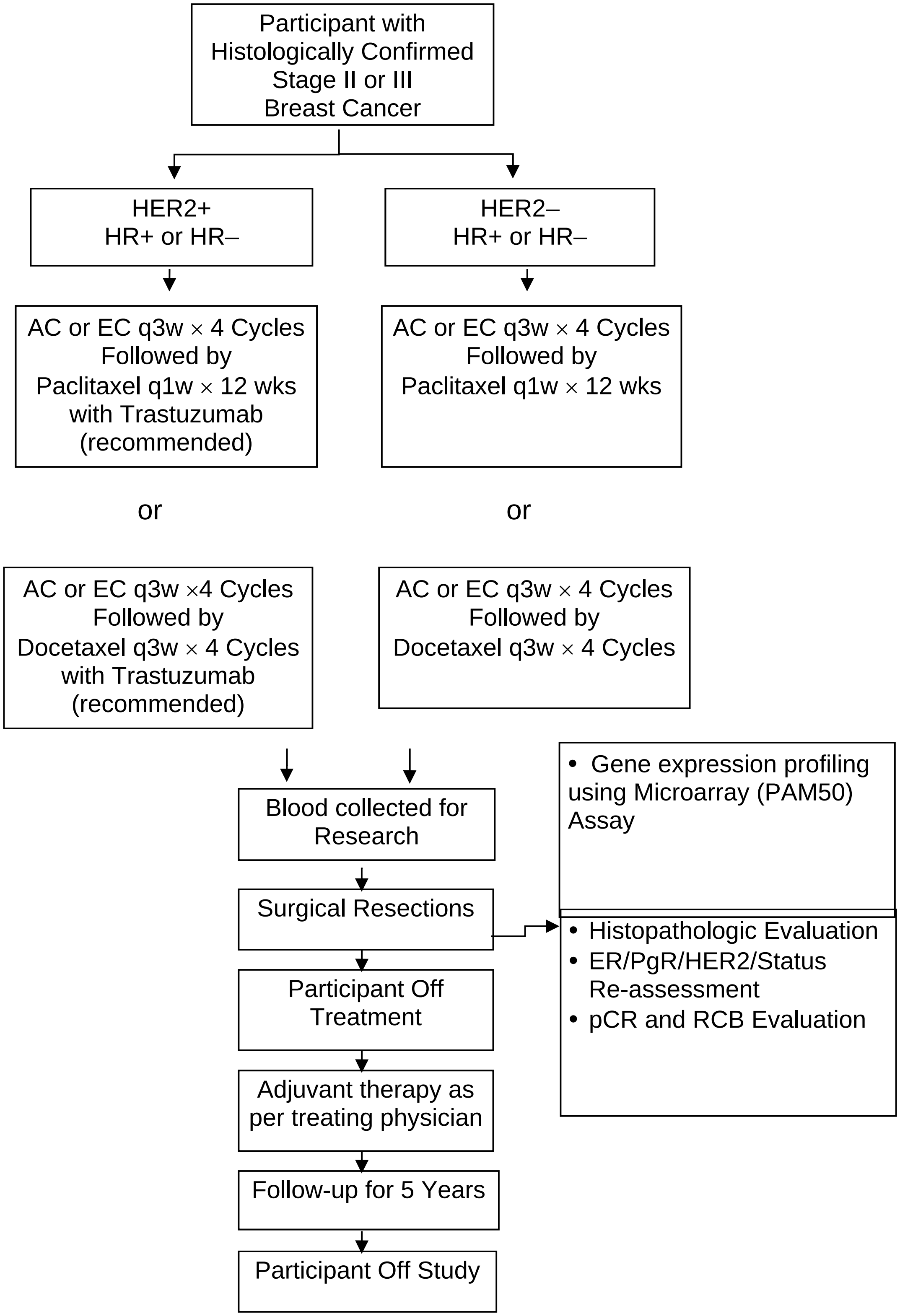

1 OBJECTIVES

1.1 Primary Objective

The primary objective of the study is to characterize the distribution of invasive breast cancer stage II or III by immunohistochemistry and gene expression profiles (luminal type A, luminal type B, human epidermal growth factor receptor 2 (HER2)-like, basal as determined by the 50-gene Prediction Analysis of Microarray (PAM 50) Assay) in Latin American women.

1.2 Secondary Objectives

- To find an association between gene expression profiles and histopathologic characteristics of the tumor prior to treatment, including histological type, size, lymph node involvement, and surrogate markers.
- To estimate the rate of pathologic complete response (pCR) to standard neoadjuvant chemotherapy in each of the breast cancer molecular subtypes and to evaluate any differences in success rates among the breast cancer intrinsic subtype cohorts. In addition, a detailed pathologic evaluation called residual cancer burden (RCB) will be used to evaluate partial pathologic response to therapy.
- To discover and develop predictive and prognostic gene expression signatures.
- To determine 3- and 5-year overall survival (OS), time to first failure (TFF) and disease-free survival (DFS) for each breast cancer intrinsic subtype and to evaluate any differences in those parameters among the subtype cohorts.
- To document the demographic and epidemiologic characteristics of each breast cancer intrinsic subtype.

2 BACKGROUND AND RATIONALE

Breast cancer is a genetically and clinically heterogeneous disease in which reliable prognostic and predictive markers are still lacking. Gene expression profiling, used to define molecular subtypes of breast cancer, showed some correlation with long-term survival and response to therapy. However, gene expression is believed to be modified by multiple variables that are not always controlled when trying to correlate molecular markers with clinical outcome. Among other factors, genetic ancestry and socio-economical and cultural factors are believed to contribute to this heterogeneity that prevents a correct classification of patients. Latin American women have a lower incidence of breast cancer than that observed in women from developed countries; this observation should be reflected at the molecular level and variations in gene expression should reflect differences at genetic and epigenetic levels in the Latin American population.

2.1 Breast Cancer

Breast cancer is a global health problem. It is the most frequently diagnosed cancer in women in Western countries, accounting for approximately 30% of all cancers diagnosed and about 16% of all cancer deaths [1]. Breast cancer is a heterogeneous disease with a wide range of clinical presentations, including but not limited to early and advanced primary tumors or extensive regional nodal involvement without systemic metastasis. It comprises a spectrum of related but different cancer subtypes, which have different causal genetic changes, follow different clinical outcomes, and require different

treatments tailored to the phenotype. The elucidation of the mechanisms underlying these biological differences is the focus of considerable ongoing investigation and the purpose of this study.

Ten to 20% of newly diagnosed cases present as locally advanced breast cancer (LABC), with a significantly higher risk of recurrence and death [2]. LABC refers to most advance-stage non-metastatic breast tumors and includes a wide variety of clinical scenarios [3]. LABC remains a clinical challenge; the majority of patients with this diagnosis eventually develop distant metastases despite appropriate therapy. Around 26% of LABC is seen in women younger than 40, prior to the age when screening is recommended [4]. In women who are being screened, 80% of LABC presents as interval cancer, a palpable mass that develops within 1 to 2 years of a normal screening mammogram [4].

The prognoses of women with LABC are also heterogeneous and depend on tumor size, extent of lymph node involvement, and the presence or absence of inflammatory carcinoma. According to the MD Anderson Cancer Center (MDACC) guidelines, any tumor greater than 5 cm or involving the skin or the chest wall is defined as locally advanced [3]. Locally advanced disease also includes fixed axillary lymph node or ipsilateral supraclavicular, infraclavicular, or internal mammary nodal involvement. According to the American Joint Committee on Cancer (AJCC) classification [5], stage II invasive breast cancer is characterized by a primary tumor either 2 cm or less in diameter (or not found) with spread to the ipsilateral axillary, supraclavicular, infraclavicular, or internal mammary lymph nodes; or a primary tumor between 2 cm and 5 cm in diameter without lymph node spread. Stage III invasive breast cancer is characterized by a primary tumor up to 5 cm in diameter or larger with lymph node spread but with no skin or chest wall involvement or spread to distal sites; or a primary tumor that has grown into the chest wall or skin, with or without lymph node involvement, and with no spread to distant sites. Thus, all stage III and a subset of stage IIB disease is considered locally advanced, whereas stage IIA and stage I tumors are considered early breast cancer. Women with stage II or III breast cancers with non-metastatic biopsy-accessible breast tumors will be included in this study.

2.2 Breast Cancer Burden in Latin America

Latin America is a rich source of genetic variations. The genomic and epidemiological perspective is applicable to breast cancer presentation and treatment because genetic subtypes of the disease have different clinical outcomes. The ability to identify different molecular profiles and their response to therapy in Latin American women would change the paradigm for the treatment of breast cancer in this population.

Latin America and the Caribbean represent approximately 10% of the population of developing countries and comprise 10% of the world's new cancer cases each year [6, 7]. However, the incidence of breast cancer is lower in Latin American countries than in more developed countries. The greatest incidence is observed in Uruguay and Argentina, with 110.9 and 88.1 cases per 100,000 inhabitants, respectively, compared with 143.8 cases per 100,000 inhabitants in the United States [6, 7]. According to projections for the year 2050, incidence and mortality rates in developing countries are expected to increase at a significantly greater rate than in developed countries. Although breast cancer incidence is higher in developed countries, the mortality rate is lower than in developing countries. These differences are likely related to differences in screening strategies and access to treatment.

Incidence and mortality statistics from registries, databases, and published literature have been reviewed and organized; basic estimators and mortality ratios for each country were calculated and trends were analyzed in order to describe the magnitude and spatial-temporal distribution of breast

cancer in Latin America and the Caribbean (LAC) from 1979–2005 [8]. Despite data gaps, increased incidence was observed. Mortality rates from breast cancer have been increasing for at least 40 years in most Latin American countries, thought to be at least partially attributable to socioeconomic development and consequent changes in reproductive behaviors [8, 9]. The relatively high mortality/incidence ratios indicate that breast cancer cases are not being adequately managed in many Latin American countries, and illustrate problems with access to detection and treatment [6–9].

A meta-analysis showed that socioeconomic deprivation may be responsible for the increased risk of breast cancer mortality in African-American and Hispanic patients, as they are more likely than white American patients to have advanced breast cancer at time of diagnosis [10]. Social deprivation is related to poor prognosis, with increased prevalence rates of high-grade estrogen receptor (ER)-negative (ER–) tumors among white women, similar to that of triple-negative breast cancers observed in African-American and Hispanic women.

Breast cancer incidence and mortality rates vary substantially among different racial and ethnic groups [11]. Latin American women are known to be an admixed population with genetic ancestry from Europeans, indigenous Americans, and Africans. A patient's ancestry rather than its race is able to provide reliable information about genetic material and breast cancer risk. A series of ancestry informative markers can be used to estimate the genetic ancestry of Latin American women participating in this study. A study conducted among Latinas in the San Francisco Bay area of United States showed that some breast cancer risk factors are associated with genetic ancestry [11]. Therefore, the authors concluded that case-control genetic association studies for breast cancer should directly measure genetic ancestry to avoid potential confounding.

2.3 Molecular Profiling and Genetic Analysis of Breast Cancer

Breast cancer is not a single disease with different morphologic features, but a group of molecularly distinct neoplastic disorders [12]. Clinicopathologic criteria guide treatment decisions, but this approach does not accurately describe tumor biology [13]. Patients with the same clinicopathologic parameters can have markedly different clinical courses and responses to treatment. Breast tumors have been traditionally classified as hormone receptor (HR)-positive (HR+) or HR-negative (HR–). Molecular profiling with the use of DNA microarrays has recently revealed four molecular classes of breast cancers, which correlate well with the traditional subgroups [13–15]. The four classes are called basal-like (HER2-negative (HER2–), HR–, or “triple negative”), luminal-A (HER2–, HR+, low Ki67), luminal-B (HER2–, HR+, high Ki67), and HER2-like (HER2+, HR+ or HR–) breast tumors [12, 16]. It is important to note that, because of the technical limitations and complexity of microarray analysis, biomarkers defined by immunohistochemistry (IHC), such as ER, progesterone receptor (PgR), and HER2, have been used as “surrogate markers” for the molecular profiles defined by microarray analysis. These molecular profiles are only associated with these protein markers, but they are not synonymous. The major limitation of this simplification is that the prognostic power of the molecular subtypes is based on complex gene expression signatures. In fact, the molecular profiles have been found to have a more robust predictive value compared with the surrogate markers. However, the correlation between the molecular subtypes and the HR/HER2 marker combinations is reasonably strong, and has enabled analysis of large datasets and discovery of important biological aspects of these tumor subtypes [16].

A large population-based study using these surrogate markers for molecular subtype examined the association of molecular subtype with clinical characteristics and found that the subtypes differed significantly by age, race, menopausal status, lymph node involvement, histology group, tumor grade,

and mitotic index [16]. Each subtype differs in molecular, prognostic, and predictive features and response to therapy. Factors that predict response to neoadjuvant chemotherapy are complex, multifactorial, and depend on multiple genes and proteins. Therefore, multiple rather than single gene markers need to be used to predict likely responses to therapy [17]. Gene microarray profiling shows potential in predicting the response to neoadjuvant chemotherapy. Thus, predictive gene expression signatures can be developed to provide information about response to chemotherapy. Similarly, prognostic gene expression signatures give information on clinical course and outcome of the disease. Gene-expression *prognostic* signatures are built by correlating gene-expression patterns, generated from tumor surgical specimens, with clinical outcomes. Gene-expression *predictive* signatures of response to treatment are generated by correlating gene-expression data, derived from biopsies taken before neoadjuvant chemotherapy, with clinical and/or pathological response to the given treatment.

Our ability to identify the optimal therapy for each individual patient is inadequate [1]. The identification of predictive markers of response to a particular drug therapy is a challenge for the biomedical field. Chemotherapy provides variable benefit to patients with breast cancer, with usually modest to severe side effects. It would be extremely advantageous if patients who are most likely to benefit from chemotherapy could be identified before or shortly after starting their treatment. Only a limited number of studies have applied a genome-wide approach using breast cancer clinical samples to investigate gene expression signatures that could predict drug sensitivity [18–24]. Although the results of these studies support the concept that predictors of response can be developed, many issues still need to be addressed (sample size, endpoints, treatment regimens) [1]. Rouzier and collaborators utilized gene microarray analysis to investigate whether the different molecular subtypes of breast cancer also respond differently to neoadjuvant chemotherapy [25]. The authors concluded that the basal-like and HER2-like subtypes of breast cancer are more sensitive to paclitaxel- and doxorubicin-containing preoperative chemotherapy than the luminal cancers.

Survival analysis performed using data from patients with LABC determined that HER2-enriched subtypes showed the poorest prognosis, with both shorter time to progression and OS [16]. Patients belonging to the luminal A subtype had a considerably better prognosis compared with all groups, and the luminal B subtype had an intermediate outcome.

2.4 Neoadjuvant Chemotherapy and Evaluation of Response

The neoadjuvant approach to chemotherapy has become more commonly used in operable breast cancer over the past 2 decades. Randomized clinical trials have shown no difference in relapse-free survival (RFS) or OS whether chemotherapy is administered before or after surgery [30–32]. However, unlike postoperative chemotherapy, neoadjuvant chemotherapy has the appeal of *in vivo* chemosensitivity testing and provides the prognostic relevance of the extent of residual disease after neoadjuvant chemotherapy, which serves as an early marker of outcome. Tumor size diminishes in most patients; this effect is observed in both the primary tumor and regional lymph nodes. This change increases the opportunity for breast-conserving surgery for patients with large or inoperable primary tumors. In addition, the neoadjuvant approach provides a vastly improved framework for correlative science studies, since measurable tumor remains in place during therapy, providing the opportunity for quick and efficient evaluation of new regimens.

While many standard chemotherapy regimens exist for neoadjuvant breast cancer treatment, the Early Breast Cancer Trialist's Group established the superiority of anthracycline-based chemotherapy regimens by performing a meta-analysis of data collected from 11 clinical trials where women were

randomized to receive regimens containing anthracyclines such as fluorouracil, doxorubicin, cyclophosphamide (FAC) or fluorouracil, epirubicin, cyclophosphamide (FEC) *versus* cyclophosphamide, methotrexate, and fluorouracil (CMF) alone [33]. The data revealed that women younger than 50 years significantly reduced their annual risk of disease relapse and death from breast cancer with polychemotherapy that included anthracyclines. In the National Surgical Adjuvant Breast and Bowel Project (NSABP)-B18 randomized clinical trial, preoperative therapy with four cycles of doxorubicin and cyclophosphamide reduced tumor size in at least 50% of patients, while 36% of patients had a complete clinical response, and more patients treated with preoperative chemotherapy were able to have breast-conservation procedures compared with patients in the postoperative chemotherapy group (68% *versus* 60%) [31]. Further, the European Organization of Research and Treatment of Cancer (EORTC)-10902 randomized trial demonstrated no improvement in DFS or OS, but showed an increased frequency of conservative surgery with the use of preoperative *versus* postoperative FEC chemotherapy [32].

Response to neoadjuvant therapy has proven to be a good surrogate for subsequent DFS [31, 34, 35]. Since it can be assessed within months rather than years, pathologic response is a particularly attractive endpoint in the rapidly evolving area of breast cancer therapeutics, allowing agility in trial development and adaptation. The best validated measure of outcome is pCR in the breast [31], which has been used as an endpoint of randomized phase 3 neoadjuvant trials such as NSABP B-27 [36]. Although significant improvements in pathologic response do not always translate into significant improvement in outcome, the value of pathologic response as an intermediate endpoint has been amply demonstrated; it is considered a surrogate marker for long-term OS. Recent studies provide us with greater ability to interpret pCR improvements. In NSABP B-27, why was the improvement in pCR so significant while the improvement in relapse-free survival was not? In fact, the modest improvement in outcome suggested by the NSABP trial is consistent with the improvement in pCR if the benefit is primarily limited to those patients who became pathologic complete responders. In the MDACC randomized trastuzumab neoadjuvant study, the addition of neoadjuvant trastuzumab to chemotherapy improved pCR by over 40%, which is also approximately the relapse benefit seen in the adjuvant trials [37, 38]. One argument against the use of pCR as an endpoint relates to the emerging understanding that different breast cancer subtypes have significantly different pCR rates to the same chemotherapy [25, 39]. The association of pCR with survival has been demonstrated within different subtypes of breast cancer [40].

RCB was proposed as a determinant of the extent of residual disease in the post-treatment surgical resection specimen of patients with breast cancer who received preoperative chemotherapy [41]. In addition to assessing response to neoadjuvant therapy by evaluating pCR (defined as absence of invasive tumor in breast or lymph nodes at the completion of all neoadjuvant chemotherapy), RCB, a more complex and detailed pathologic evaluation, will be used to evaluate surgical specimens. RCB is estimated from routine pathologic sections of the primary breast tumor site and the regional lymph nodes after completion of neoadjuvant therapy. Six variables are included in the MDACC algorithm for RCB including tumor bed size, cellularity, and extent of the disease in the breast and nodes; the algorithm is available online at **www.mdanderson.org/breastcancer_RCB**. RCB is potentially a better predictor of 5-year RFS.

3 SUMMARY OF STUDY PLAN

The primary objective of this study is to characterize the distribution of molecular profiles in Latin American women with AJCC 7 clinical stage II or III breast cancer. The breast cancer intrinsic subtypes as determined by the PAM 50 Assay and immunohistochemistry will be correlated with epidemiological,

histological, and clinical characteristics, including pathologic response to standard neoadjuvant chemotherapy. Moreover, this study intends to define a molecular signature that will predict response to neoadjuvant therapy in breast cancer. This is a prospective cohort study in which no investigational drugs will be administered to participants. The study will be conducted at participating institutions in Argentina, Brazil, Chile, Mexico, and Uruguay. The study has two parts:
Part A is a descriptive, observational part to characterize the molecular profile of breast cancer in Latin American women; Part B, the standard neoadjuvant chemotherapy treatment part of the study, seeks to identify any associations between response to neoadjuvant therapy and the molecular profiles. Participants will be followed for a period of 5 years to determine any associations between the molecular profiles and disease evolution following standard treatment. In addition to providing valuable information on the effectiveness of standard chemotherapy in Latin American women, Part B of the study (neoadjuvant chemotherapy arm) will build infrastructure in the US Latin America Cancer Research Network (US–LA CRN) for conducting additional clinical trials incorporating complex genomic and pathologic biomarkers, including trials to evaluate investigational therapies in the Latin American population.

Women with non-metastatic, AJCC 7 clinical stage II or III breast cancer are eligible for the first part of the study (see Schema, Figure A). After participants sign the informed consent form, they are enrolled in the study and an epidemiologic questionnaire is completed. Participants then undergo a routine standard-of-care clinical, radiographic, and surgical evaluation. The treating physician will recommend the appropriate standard-of-care treatment for the participant—either primary surgery or neoadjuvant chemotherapy followed by surgery—following local institutional guidelines. Baseline blood samples for future correlative studies will be collected prior to treatment; correlative studies using blood or tissue samples collected during this study will require detailed amendments to the current protocol and informed consent or a separate protocol and additional informed consent. These studies will undergo both scientific and IRB review, and participants will be re-consented. Tissue samples for research in this study (*e.g.*, gene expression profiling by PAM 50 assay) will be collected either during the routine core biopsy procedure (for participants on the neoadjuvant arm) or during surgery (for participants on the primary surgery arm). In other words, participants who are candidates for neoadjuvant chemotherapy will undergo a pre-treatment core biopsy procedure for histopathologic evaluation and tumor ER, PgR, HER2, and Ki67 status assessment in addition to the routine diagnostic work-up. Participants on the neoadjuvant arm will also have core biopsy specimens taken for research purposes; this may be done at the same time as the diagnostic biopsy or following diagnostic biopsy and prior to beginning neoadjuvant chemotherapy. Participants who are candidates for primary surgery without neoadjuvant chemotherapy are not required to undergo a core biopsy procedure; this decision should be based on local institutional standard-of-care guidelines. In this case, the histopathologic evaluation, biomarker assessment, and collection of tissue for gene expression profiling by PAM 50 assay is done on surgical resection specimens collected during surgery.

After histological and clinical confirmation of stage II or III breast cancer, participants eligible for neoadjuvant chemotherapy will continue to be enrolled in Part B of the study. Participants who do not meet the inclusion and exclusion criteria for Part B of the study will be considered off study at this point. All participants regardless of whether they are on or off study will continue receiving standard-of-care treatment. During Part B of the study (see Schema, Figure B), participants will be categorized according to their hormone receptor and HER2 status and will then receive standard neoadjuvant chemotherapy as recommended in protocol. A second blood sample will be collected and stored after completion of chemotherapy and before surgery for future correlative studies which will require a protocol amendment or new protocol and re-consent of patients. Tissue samples will be collected from surgical

resections for histopathologic evaluation and ER, PgR, HER2 status re-assessment, to evaluate pathologic response to neoadjuvant treatment, and for gene expression by PAM50 assay. Pathologic complete response is defined as absence of invasive tumor in breast or lymph nodes at the completion of all neoadjuvant chemotherapy. A more detailed pathologic evaluation, RCB, will be used to evaluate surgical specimens. Predictive and prognostic gene expression signatures in the Latin American women will be developed. Participants on study (from both primary surgery and neoadjuvant chemotherapy arm) will be followed up to 5 years to capture survival data and clinical outcome. Decision for additional adjuvant therapy is at the discretion of treating physician. Participants will be considered off study after the 5-year follow-up period.

4 INCLUSION AND EXCLUSION CRITERIA

4.1 Overview

Women who meet the initial inclusion and exclusion criteria (see section 4.2) will be offered participation in the study. Women who decide to sign the informed consent will be subsequently enrolled in the study. The treating physician will recommend the appropriate standard-of-care treatment according to local institutional guidelines. Only participants with histologically-confirmed breast cancer who meet staging criteria indicated below (sections 4.2 and 4.3) will continue on study; participants who do not meet additional eligibility criteria for the study must be taken off study. Irrespective of whether on study or off study, all women will receive standard-of-care treatment according to local institutional guidelines.

4.2 Inclusion and Exclusion Criteria for Part A of the Study:

Inclusion Criteria:

- Women age ≥ 18 years.
- AJCC 7 clinical stage II or III breast cancer. Clarification: Participants with clinical stage II breast cancer who are later classified as histologically-confirmed stage I will remain on study; participants who are later classified as histologically-confirmed stage IV breast cancer will be taken off study.
- Biopsy-accessible breast tumor or participant candidates for primary surgery.
- Eastern Cooperative Oncology Group (ECOG) performance status 0−1 (see Appendix A).

Exclusion Criteria:

- Prior history of non-breast malignancy (excluding in situ cancers treated only by local excision and basal cell and squamous cell carcinomas of the skin) within 5 years prior to enrollment in this study.
- Bilateral invasive or in-situ breast cancer.
- Inflammatory breast cancer.
- Clinical or radiological evidence of distant metastases by computed tomography (CT), chest X-ray, abdominal/thoracic ultrasound, bone scan, and/or liver function tests including total bilirubin, alanine aminotransferase (ALT), aspartate aminotransferase (AST) and alkaline phosphatase (ALP), within ranges defined in section 4.3.
- Prior hormone therapy, chemotherapy, biologic, targeted therapies, or radiation therapy for this malignancy. Prior bisphosphonate therapy is allowed.
- Pregnant and lactating women: Effects on a developing human fetus of chemotherapeutic agents at the recommended therapeutic dose remain incompletely defined. For this reason and because these agents may be teratogenic, women of child-bearing potential must agree to use adequate contraception (double barrier methods of birth control or abstinence) prior to study entry and for the duration of study treatment phase. Should a woman become pregnant or suspect she is pregnant while participating in this study, she should inform her study physician immediately.
- Because there is an unknown but potential risk for adverse events in nursing infants secondary to treatment of the mother with chemotherapeutic agents, women who are breastfeeding will be excluded. If a participant is of child-bearing potential (women are not considered of childbearing potential if they are at least 2 years postmenopausal and/or surgically sterile), she must have documented negative serum or negative urine pregnancy tests within 14 days of entry to the study (i.e., within 14 days of signing the informed consent document).
- Subjects with psychiatric illness and/or other specific situations that would limit compliance with study requirements and compromise participant follow-up.
- Lack of ability to understand and willingness to sign a written informed consent document.

Note: Subjects who were enrolled prior to this amendment will be considered eligible even if the HIV/Hep C and pregnancy test were not preformed due to each country standards.

4.3 Inclusion and Exclusion Criteria for Part B of the Study:

Participants, who successfully enrolled into the first part of the study and who, according to local institutional guidelines, are candidates for neoadjuvant chemotherapy will participate in Part B of the study. Participants must also meet the inclusion and exclusion criteria described below.

Inclusion Criteria:

- Histologically confirmed new primary adenocarcinoma of the breast AJCC 7 clinical stage II or III. All histological types are included Hormone status: Any tumor ER/PgR status, any HER2/neu status as measured by local hospital pathology laboratory following US–LA CRN standard operating procedures (SOPs).

- Normal organ and marrow function as defined below:
  - Absolute neutrophil count ≥1500/μL
  - Platelets ≥100,000/μL
  - Total bilirubin within normal institutional limits, unless participant has Gilbert's disease, for which bilirubin must be ≤ 2.0 × upper limits of normal (ULN)
  - AST serum glutamic-oxaloacetic transaminase (SGOT)/ALT serum glutamic-pyruvic transaminase (SGPT) ≤1.5 × institutional ULN
  - ALP ≤ 2.5 x institutional ULN
  - Creatinine ≤ 1.5 × institutional ULN
  - Negative serum or urine beta-human chorionic gonadotropin (HCG), unless participant is post-hysterectomy or menopausal.

Exclusion Criteria:

- Uncontrolled or severe cardiac disease. Baseline left ventricular ejection fraction (LVEF) by nuclear imaging or echocardiography must be within normal institutional limits.
- Use of any investigational agents within 30 days of starting standard chemotherapy treatment.
- History of allergic reactions attributed to compounds of similar chemical or biologic composition to chemotherapeutic agents or accompanying supportive medications.
- Uncontrolled intercurrent illness including, but not limited to, ongoing or active infection, symptomatic congestive heart failure, unstable angina pectoris, cardiac arrhythmia, or psychiatric illness and/or other specific situations that would limit compliance with study requirements and compromise participant follow-up.

4.4 Inclusion of Women and Minorities

Latin American women with breast cancer of all ethnic backgrounds will be included in this study as participants. There are no expected racial/ethnic differences in the recruitment effort.

4.5 Recruitment and Retention Plan

Participant eligibility will be systematically assessed at each of the participating US–LA CRN study sites. A screening log will be kept documenting the review of potentially eligible participants as well as reasons for non-enrollment. Sites will provide detailed information to all relevant treating physicians on the conduct of the trial to optimize physician participation. Regular conference calls every other month will review recruitment at each site so that sites not meeting recruitment goals can be identified early and interventions to improve recruitment can be instituted.

5 BIOSPECIMENS FOR BIOMARKER, GENETIC, AND GENOMIC ANALYSES

5.1 Part A of the Study: Gene Expression Profiling by PAM-50 Assay

During the first part of the study, tissue will be acquired from core needle biopsies or from surgical resections (Schema, Figure A). Participants who are candidates for neoadjuvant chemotherapy prior to surgery will undergo a routine, standard-of-care diagnostic core needle biopsy procedure for histological

confirmation of breast cancer diagnosis; tissue samples for research purposes will be collected at that time or after diagnostic procedures. Participants who are candidates for primary surgery are not required to undergo a core biopsy procedure prior to surgery. (This decision should be made following local institutional guidelines.) In this case, tissue samples for this study will be collected at the time of surgery. Thus, core needle biopsy samples will be obtained from participants presenting with biopsy-accessible non-metastatic AJCC 7 clinical stage II or III breast cancer. A minimum of four and a maximum of seven 14-gauge core biopsies will be collected from each participant during the first part of the study: two to three cores (depending on institutional guidelines) will be fixed in formalin for diagnostic purposes and two to four core biopsies will be snap-frozen in liquid nitrogen for research purposes.

Formalin-fixed/paraffin-embedded (FFPE) samples will be utilized for routine diagnostic work-up including histopathologic evaluation of the tumor by hematoxylin and eosin (H&E) analysis and assessment of expression of tumor ER, PgR, HER2, and Ki67 status by IHC and complemented by HER2 fluorescent *in situ* hybridization (FISH) or chromogenic *in situ* hybridization (CISH) as appropriate (for HER2 IHC 2+ cases). Samples will be processed at the local pathology laboratory at each institution. The pathology laboratories performing IHC, FISH, and CISH procedures will utilize commercial kits produced by the same manufacturer (and from the same lot, if possible) and will follow the manufacturer's protocol and instructions to reduce variability of results across the sites. Some laboratories will perform the FISH assay for confirmation of HER2 status and others will perform the CISH assay depending on the expertise of the personnel and the instrumentation available. The interpretation of IHC, FISH, and CISH assays, as well as reporting of results, has also been standardized for all sites; those SOPs are included in the Manual of Operations (MOP) for this study.

Snap-frozen tissue samples collected during Part A of the study will be utilized to extract RNA and subsequently perform DNA microarrays to characterize the distribution of breast cancer intrinsic subtypes by PAM 50 assay in the Latin American women population, which is the primary objective of this research study. In the event that both biopsy and surgical resection samples pre-chemotherapy are available from a participant, RNA will be extracted from the core biopsy sample for DNA microarrays. These gene expression profiles will be correlated with pathologic measures after neoadjuvant chemotherapy (Part B of the study, Schema, Figure B). As part of the research the gene expression data will also be used to develop predictive gene expression signatures and/or to validate previously described signatures.

DNA microarrays will be performed using a common Agilent gene expression platform; arrays will be processed in central molecular biology laboratories located in Argentina (Instituto Leloir), Brazil (Instituto do e Cancer do Estado de Sao Paulo and Hospital do Cancer do Barretos), Mexico (University of Guadalajara and University of Sonora), Chile (Instituto de Salud Publica de Chile) and Uruguay (Instituto Pasteur). All laboratories will utilize a custom-designed Agilent human 4x44k Gene Expression Array. All laboratories will follow the same Microarray SOPs which are included in the MOP of the Study; total RNA isolation, quantification, labeling, hybridization, scanning, data processing, and analysis have been harmonize for this study across all the participating laboratories. All laboratory technicians performing the assay have or will receive training on the Study SOPs for DNA microarrays and have conducted a pilot study to evaluate the proficiency of the lab technician performing the assay. The Agilent Feature Extraction software is used by all laboratories to generate raw data which is uploaded into OpenClinica. All raw data will be subsequently automatically normalized and analyzed using a single microarray pipeline based on the 50-gene Prediction Analysis of Microarray (PAM 50) assay to determine the breast cancer intrinsic subtype of each patient. The genetic information derived from the PAM 50 assay will not

have clinical applicability as it will not affect the clinical care of participants enrolled in this study, therefore, this information will not be shared with the participants.

Blood samples for research purposes will be collected during the first part of the study, at baseline, from all participants before core biopsy procedure or primary surgery (Schema, Figure A). Future correlative studies using blood or tissue samples collected during this study will require detailed amendments to the current protocol and informed consent or separate protocols. Future amendments or protocols will undergo both scientific and IRB review, and participants will be re-consented.

5.2 Part B of the Study: Neoadjuvant Chemotherapy

During Part B of the study (Schema, Figure B), at the time of surgery after neoadjuvant chemotherapy, tumor and normal breast tissue as well as lymph nodes will be collected. The surgical assessment will be used to determine pCR. The surgical specimens will also be assessed using standard histopathologic parameters and the RCB technique to evaluate the extent of residual disease by the site pathologist; information will be recorded in the Surgical Evaluation Case Report Form (CRF). RCB is estimated from routine pathologic sections of the primary breast tumor site and the regional lymph nodes after completion of neoadjuvant therapy. RCB components will be collected by the study local pathologist, who will be trained in the technique. According to SOPs for surgical resections, if no gross cancer is identified at the time of surgery, the entire area including the fibrosis must be fixed in formalin and submitted for microscopic examination to determine whether the participant achieved pCR and to assess RCB. If gross cancer is identified, then tumor, normal, and involved lymph nodes (only if excess gross metastatic cancer is present in the lymph node) should be formalin-fixed and snap-frozen. Uninvolved lymph nodes will be formalin-fixed only. SOPs for evaluation of pathologic response to therapy are part of the MOP of the study.

FFPE samples will be utilized for histopathologic evaluation of the tumor by H&E analysis and for re-assessment of expression of tumor ER, PgR, and HER2 status.

Snap-frozen tissue samples collected from surgical resections during Part B of the study will be used for gene expression analysis by PAM-50 assay, and for developing gene expression prognostic signatures, built by correlating gene expression data from surgical resections after neoadjuvant chemotherapy and survival data.

Blood samples for research purposes will be collected during Part B of the study from all participants after neoadjuvant chemotherapy/before surgery (Schema, Figure B). Serum, plasma, whole blood and buffy coat cells will be stored at biobanks for future correlative studies for which detailed amendments to the protocol and informed consent or separate protocols would be developed. These amendments or new protocols would undergo both scientific and IRB review and patients re-consented..

5.3 Biospecimens Collection, Handling, Processing, Labeling, and Storage

All biospecimens including biopsies, surgical resections, and whole blood will be collected, handled, processed and stored according to US–LA CRN SOPs and the labeling guidelines which are included in the MOP for this study. Biopsies will be analyzed by a local pathologist following local institutional standards, US–LA CRN SOPs, and College of American Pathologists Breast Cancer Protocol [42].

5.4 BSI-II^{TM}

All the above-mentioned procedures will be conducted at local institutional pathology laboratories. All specimens and specimen transformation will be recorded and tracked in BSI-II^{TM}, a tissue bank repository tool for biospecimen inventory, tracking, and basic annotation provided by the National Cancer Institute (NCI) (See Section 10.1 below). BSI-II^{TM} permits users to track the collection, storage, quality assurance, and distribution of specimens, as well as the derivation and aliquoting of new specimens from an existing one (*e.g.*, for DNA analysis, biomarker, or clinical chemistry assays). It is also possible to track the storage location as well as follow shipments, if necessary, of all samples in this study utilizing BSI-II^{TM}. The BSI-II^{TM} user manual is available online at **http://www.bsi-ii.com/support/**. Training has already been provided to study staff, and additional training (*e.g.*, training on data management and in proper placement of samples from the clinical [collection] site in each country's specimen biobank inventoried for later use in cancer research) will be provided to the technical staff, pathologist, and researchers, as necessary.

6 CRITERIA FOR EVALUATION AND ENDPOINT DEFINITION

6.1 Primary Endpoint

The primary endpoint of the study is the distribution of molecular profiles of breast cancer (AJCC 7 clinical stage II or III) in Latin American women.

6.2 Secondary Endpoints

- Association of each breast cancer intrinsic subtype with tumor histological type, size, lymph node status and surrogate markers.
- Proportion of participants in each breast cancer intrinsic subtype who achieve pCR to neoadjuvant chemotherapy.
- RCB following neoadjuvant chemotherapy.
- Predictive and prognostic gene expression signatures.
- Survival: OS, DFS, and TFF. OS and TFF will be measured for all participants from enrollment in the study. DFS will be measured from definitive surgery for those participants who undergo definitive surgery. OS and DFS will be evaluated up to 5 years post-surgery.
- Demographic and epidemiologic characteristics of the population by breast cancer intrinsic subtype.

6.3 Target Lesion

6.3.1 Pathologic Assessment

In the event of multifocal or multicentric disease in the breast, the investigator must determine which will represent the target lesion. This should remain consistent throughout the study. The target lesion should be selected on the basis of its size (lesion with the longest diameter) and suitability for accurate repetitive clinical measurements.

6.3.2 Pathologic Complete Response

pCR is defined as the absence of residual invasive carcinoma in the breast (at the time of definitive surgical resection) and in the lymph nodes (no invasive tumor by H&E); ductal carcinoma *in situ* (DCIS) is

allowed. In other words, participants could achieve a pCR if they have no microscopically viable invasive tumor in the breast and axillary nodes at surgery; *i.e.*, participants with residual DCIS could also achieve a pCR.

6.3.3 RCB

RCB was proposed as a determinant of the extent of residual disease in the post-treatment surgical resection specimen of participants with breast cancer who received preoperative chemotherapy. Six variables are included in the MD Anderson algorithm for RCB which can be found online at **www.mdanderson.org/breastcancer_RCB.** In brief, the variables include cross-sectional dimensions of the residual tumor bed (d1 and d2), estimate of the proportion of that residual tumor bed area that is involved by cancer (%CA), estimate the proportion of the cancer that is *in situ* component (%CIS), number of positive lymph nodes (LN), and measure the diameter of the largest nodal metastasis (dmet). The RCB index value can also be calculated and involves the categorization into one of four RCB classes (RCB 0 or pCR, RCB I or near pCR, RCB II, RCB III). RCB components will be collected by the study local pathologist, who will be trained in the technique. The US–LA CRN Pathology Committee will make the final determination on any indeterminate and contested results.

6.4 Clinical Assessments

Both target lesions, and in the event of multifocal or multicentric invasive breast cancer, non-target lesions, should be followed clinically and their clinical size measured with a caliper and recorded at baseline. Measurements thereafter are required every three weeks during neoadjuvant chemotherapy and before surgery. If there is evidence of progression, the investigator (participant's physician) should be notified in order to determine whether the participant should come off protocol treatment. *In situ* carcinoma does not represent a non-target lesion and should not be recorded or followed.

6.5 Off-Study Criteria

Participants may go off study for the following reasons: the protocol intervention and any protocol-required follow-up period is completed; unacceptable toxicity to chemotherapy; lost to follow-up; non-compliance; concomitant medication; medical contraindication; withdraw consent; or death.

6.6 Study Termination

The NCI Center for Global Health (CGH) and the participating Latin American institutions in Argentina, Brazil, Chile, Mexico, and Uruguay—as the study co-sponsors—have the right to discontinue the study at any time.

7 CHEMOTHERAPY ADMINISTRATION

During Part B of the study (Schema, Figure B), participants will receive standard-of-care neoadjuvant chemotherapy for a total of 16 to 24 weeks. Surgery must be performed within 42 days following completion of chemotherapy. No new and/or investigational agents, doses, or duration of exposure will be tested in this study.

7.1 Neoadjuvant Chemotherapy Administration

Participants who are HER2+, HR+, or HR– receive one of the following standard-of-care neoadjuvant chemotherapy regimens:

- Regimen #1: Doxorubicin (60 mg/m^2) or epirubicin (75 to 100 mg/m^2) + cyclophosphamide (600 mg/m^2) every 3 weeks for 4 cycles followed by paclitaxel (80 mg/m^2) weekly for 12 weeks with or without trastuzumab (4 mg/kg loading dose followed by 2 mg/kg) weekly for 12 weeks.

OR

- Regimen #2: Doxorubicin (60 mg/m^2) or epirubicin (75 to 100 mg/m^2) + cyclophosphamide (600 mg/m^2) every 3 weeks for 4 cycles followed by docetaxel (70–90 mg/m^2) every 3 weeks for 4 cycles with or without trastuzumab (8 mg/kg loading dose followed by 6 mg/kg) every 3 weeks for 4 cycles.

Participants who are HER2–, HR+ or HR– receive one of the following standard-of-care neoadjuvant chemotherapy regimens:

- Regimen #1: Doxorubicin (60 mg/m^2) or epirubicin (75 to 100 mg/m^2) + cyclophosphamide (600 mg/m^2) every 3 weeks for 4 cycles followed by paclitaxel (80 mg/m^2) weekly for 12 weeks.

OR

- Regimen #2: Doxorubicin (60 mg/m^2) or epirubicin (75 to 100 mg/m^2) + cyclophosphamide (600 mg/m^2) every 3 weeks for 4 cycles followed by docetaxel (70–90 mg/m^2) every 3 weeks for 4 cycles.

Chemotherapeutic drugs will be administered intravenously. Doses should be based upon actual body weight and not ideal body weight. If a participant's body weight increases or decreases by ≥ 10% from baseline during the course of the treatment phase, the body surface area and drug dose should be recalculated. It is recommended that the treating physician follows local institutional guidelines for pre-medication for chemotherapy treatment.

Approved and marketed drugs will be used in this study. Refer to the drug box for lot number information and to the package insert for additional pharmaceutical information.

Since there are no investigational drugs planned for this study, each country/site will be following reporting of standard-of-care drug toxicity per their established requirements.

7.2 Adjuvant Chemotherapy Administration

There are no adjuvant treatment requirements for this trial. Adjuvant therapy is at the discretion of the treating physician and participant; adjuvant therapy administered to the participants is captured in the CRF. However, it is recommended that participants receive the standard of care following neoadjuvant chemotherapy and surgery, including hormonal therapy for a minimum of 5 years if ER+, 1 year of trastuzumab if HER2+, and radiation therapy if indicated.

8 CLINICAL AND RESEARCH EVALUATIONS AND PROCEDURES

8.1 Schedule of Events Table*

Evaluation/Procedure	Enrollment/ Baseline	Every 3 Weeks During Neoadjuvant Therapy	Prior to/at Surgery	Follow-up
Tests and Observations				
Informed Consent*	X			
Epidemiology Questionnaire*	X			
Medical History	X	X		X
Physical Exam	X	X	X	X
Clinical Tumor Measurement	X	X	X	
MUGA/ECHO	X[a]	X[a]	X[a]	
Laboratory Studies				
Laboratory Blood Tests	X	X		
Pregnancy Test: Serum βHCG	X			
IHC	X[c]		X[c]	
Radiographic Evaluation				
Mammogram	X		X	
Chest X-ray	X			
Bone scan	X			
Abdominal Ultrasound	X			
Thoracic/Abdominal CT	X[b]			
Biospecimens Collection				
Core Biopsy Tissue Collection	X			
Surgical Resection Tissue Collection	X		X	
Blood Drawn for Serum, Plasma and Buffy Coat *	X		X	
Microarrays (PAM 50 Assay)	X[c]		X[c]	
Evaluation of response			X	X
Adjuvant Therapy (per treating physician's discretion)				X

[a] MUGA or ECHO performed in HER2+ participants receiving trastuzumab. Repeat measurement of LVEF should be performed using the same method as the baseline evaluation (MUGA or ECHO) every 3 weeks during neoadjuvant treatment and prior to surgery (or following local institutional guidelines).

[b] Abdominal and thoracic CT will be performed for T3/4 N2/3 tumors.

[c] On core biopsy (baseline) and surgical tissue (post neoadjuvant chemotherapy) for patients in Neoadjuvant Chemotherapy Arm. On surgical tissue (baseline) for patients in Primary Surgery Arm.

*Procedures in **bold** and marked with an asterisk indicate research procedures **only**. Refer to section 5.1 for details about number of core biopsies for diagnostic clinical purposes versus research purposes.

NOTE: Refer to the 'CRFs and Quality Control Forms Completion Guidelines' for a comprehensive list of CRFs to be completed for each visit.

8.2 Enrollment/Baseline

The IRB-approved informed consent form will be carefully reviewed with each potential participant. All patients will be required to sign the informed consent form prior to enrollment in the study.

All participants will receive the following clinical routine standard-of-care procedures:

- Medical history and physical exam (including collection of height, weight, ECOG score).
- Clinical tumor measurement with a caliper.
- Multigated acquisition scanning (MUGA) scan or echocardiogram (ECHO) performed on HER2+ participants receiving trastuzumab.
- Blood sample will be collected for complete blood count (CBC) with differential and blood chemistry panel as well as a serum pregnancy test if required.
- Radiographic evaluation including a mammogram, chest x-ray, bone scan, abdominal ultrasound, and an abdominal and thoracic computed tomography (CT) scan for participants with T3/4 N2/3 tumors.

  Participants with non-metastatic biopsy-accessible AJCC 7 clinical stage II or III breast cancer who meet all inclusion/exclusion criteria (see section 4.2) and who are not candidates for primary surgery will undergo a core biopsy (14-gauge needle). Two to three core biopsies will be fixed in formalin for routine diagnostic histopathologic evaluation.

All participants will undergo the following research procedures:

- Two to four additional core biopsies will be collected at the time of the biopsy procedure and snap-frozen for research purposes (e.g., assays to determine the status of clinical biomarkers (ER, PgR, HER2, Ki67) and gene expression profiling by PAM 50 assay) to meet the primary objective of the study. Samples will be collected from all participants following US-LA CRN SOPs.

Note: Total number of core biopsies (clinical + research) will range from four to seven.
A research blood sample will also be obtained for serum, plasma, and buffy coat.

- Blood samples for future correlative studies will be collected and stored in the biobanks.
- Complete an epidemiology questionnaire regarding socio-economic and reproductive characteristics, family history of cancer, and factors associated with access to diagnosis and treatment of breast cancer (see Appendix B).

All participants with histologically confirmed stage II or III breast cancer and who fulfill all inclusion/exclusion criteria for the second part of the study (see section 4.3) will be assigned to the neoadjuvant chemotherapy arm. Both on- and off-study women continue receiving local institutional standard-of-care treatment.

8.3 Baseline Surgical Evaluation

After completing the routine screening tests and procedures, the participant must be seen and examined by the treating surgeon. This will include a clinical breast and lymph node examination and review of imaging studies of the breast and axilla. After examining the participant and reviewing the pertinent radiologic studies, the surgeon will determine whether the participant is a candidate for primary surgical treatment.

Some clinicians may choose to determine the pathologic axillary status prior to beginning treatment. This can be done by whatever means the surgeon feels is appropriate.

The treating physician will then recommend the appropriate standard-of-care treatment according to local institutional guidelines. Participant management will be solely based on institutional standard-of-care treatment and not on research results obtained from gene expression profiling. Thus, some participants will undergo primary surgery while others will receive standard neoadjuvant chemotherapy prior to surgery. It is up to the treating physician to determine which clinical treatment is most appropriate for each participant.

8.4 Neoadjuvant Chemotherapy

Participants will be categorized based on three clinical biomarkers (or surrogate markers): HRs ER and PgR and HER2. Therefore, there are four possible subtypes: HER2+ HR+, HER2+ HR–, HER2– HR+, HER2– HR–. Participants will receive chemotherapy for a total of 24 weeks (see Section 7.1 for drug regimens). Surgery must be performed within 42 days following completion of chemotherapy. Protocol-defined therapy ends at surgery; however, adjuvant therapy at the discretion of the participant's oncologist is recommended, including hormonal and radiation therapy.

During neoadjuvant chemotherapy, participants will return to the clinic every three weeks and undergo the routine examination according to physician's recommendation. That might include but not be limited to a physical exam, clinical tumor measurement, CBC with differential, and blood chemistry panel as well as a serum pregnancy test if required, and MUGA scan or electrocardiogram if receiving trastuzumab.

Participants who are enrolled on the neoadjuvant arm of the study but elect to have primary surgery will remain on the neoadjuvant arm for purposes of study analysis but will not be evaluable for response to therapy. This will be recorded on CRF "Off treatment".

8.5 Pre-surgery Evaluation after Neoadjuvant Chemotherapy

Prior to surgery, following the completion of neoadjuvant chemotherapy, participants will again be examined by the treating surgeon and will be assessed as to whether or not they are candidates for surgery (either conservative or radical mastectomy). All participants should undergo appropriate surgical management unless they are inoperable or surgery is medically contraindicated. If they are not, the reasons will be documented on a Surgical Evaluation Form. If the participant is a breast conservation candidate but opts for a mastectomy, this will also be documented. Prior to surgery, all participants must undergo a physical exam, CBC with differential, blood chemistry panel for clinical purposes, repeat breast and axillary examination with clinical tumor measurements, mammography, and if treated with trastuzumab, a MUGA scan or echocardiogram for LVEF. A research blood sample will be obtained. A pre-surgical (after the last chemotherapy administration) mammogram is required. If the participant clinically progresses, repeat imaging is required. If there is discordance (clinical progression, but radiographic stable disease or response), contact the local study chair.

8.6 Surgery

Surgery (breast conservation or total mastectomy) will be performed with appropriate management of the axilla. The choice of breast conservation therapy or mastectomy will be at the discretion of the surgeon and the participant's preference if applicable. For participants undergoing breast conservation, documentation of negative margins is required. It is strongly recommended that the tumor be excised to

free surgical margins of both invasive carcinoma and DCIS, with the exception noted of posterior margins if this margin is pectoral major fascia or the anterior margin or dermis. Participants who have a tumor-involved partial mastectomy margin (except the posterior margin at the pectoralis fascia or anterior margin at the dermis) require re-excision. This may be accomplished with a re-operative partial mastectomy or with a total mastectomy. This choice will be at the discretion of the treating surgeon.

It is required that all participants with pretreatment T4 or N2 disease have a level II axillary node dissection at the time of the definitive breast cancer operation. The use of sentinel lymph node biopsy is not accepted after neoadjuvant treatment.

It is required that any participant with a pre-treatment sentinel node with N1 (microscopically positive) undergo a post-treatment level II axillary node dissection.

8.7 Post-treatment Surgical Specimen Submission

Submission of the Surgical Evaluation Form by the institutional study pathologist is required. This form captures the pathologic details of residual invasive carcinoma, DCIS, and lymph node status. Frozen as well as formalin-fixed resection samples will be taken at the time of surgery according to US–LA CRN SOPs for collection, handling, processing, and storage of surgical resections.

8.8 Follow-up Period

The participant will be followed for 5 years after the date of surgery for survival and recurrence. Follow-up data will be collected every 6 months for 5 years. Participants will be considered off study after the 5-year follow-up period; at that time the study will be considered completed.

9 STATISTICAL CONSIDERATIONS

9.1 Study Design/Objectives

The study design is partitioned into two parts. Part A is a descriptive, observational research that seeks to characterize the distribution of IHC and gene expression profiles (as determined by microarray assessments using the PAM 50 assay) in Latin American women with stage II or III breast cancer.

- Luminal A
- Luminal B
- Basal
- HER2-like

Part B is a standard-of-care neoadjuvant chemotherapy portion that seeks to identify any associations between response to therapy and the four classes of surrogate biomarker combinations as determined by IHC and/or FISH/CISH and the four classes of molecular profiles mentioned above.

- HER2+, HR+
- HER2+, HR–
- HER2–, HR+
- HER2–, HR–

9.2 Sample Size/Accrual Rate

For Part A, traditional sample size and power calculations are not appropriate; the objective of this portion is to describe the population by their gene expression profile and not to demonstrate that one cohort is statistically different from another. Instead, means and proportions and corresponding 95% confidence intervals will be the descriptors of the characterizations.
For Part B, it is assumed that the distribution of breast cancer subtypes in Latin American women is similar to that observed in the general population, which is HER2+ HR+ ~ 45%, HER2– HR+ ~ 25%, HER2+ HR– ~ 15%, and HER2– HR– ~ 15%. Thus, assuming a total sample size of 2000 subjects, subtotals ranging from 300 to 900 per surrogate biomarker class, and a fixed two-tailed $\alpha=0.05$, sample size and power will depend on the following:

- Which class pairs are being considered
- Their possible respective pCR probabilities

In terms of **sample size**, assuming a clinically meaningful difference in pCR rates of 10 percentage points, statistically discriminating between success rates of 10% *versus* 20% will require fewer participants than doing so for 45% *versus* 55%; all other 10-point difference pairs (*e.g.*, 60% *versus* 70%, 30% *versus* 40%) require sample sizes that are intermediate to those above. Intergroup differences larger than 10%-points will require fewer participants.

Assuming a total sample size of 2000 across all four cohorts, the power to statistically discriminate among surrogate marker classes is dependent upon the cohort sample sizes and their respective response rates. Based on preliminary estimates of cohort frequencies, the estimated samples sizes for the cohorts are listed in the table below. The worst-case scenario comes from comparing rates of 45% *versus* 55% (power=65–94%). Best-case scenario would be HER2+, HR+ (n=900) *versus* HER2–, HR+ (n=500) at rates of 10% *versus* 20% (or 90% *versus* 80%, since the curve is symmetrical on either side of 50%); here power equals 99%. The matrix below provides the power with each class-pair at worst case (upper triangle) and best case (lower triangle):

POWER	HER2+, HR+ n=900	HER2–, HR+ n=500	HER2+, HR– n=300	HER2–, HR– n=300
HER2+, HR+ n=900	-	94%	83%	83%
HER2–, HR+ n=500	99%	-	76%	76%
HER2+, HR– n=300	98%	96%	-	65%
HER2–, HR– n=300	98%	96%	91%	-

Therefore, assuming that the distribution of breast cancer subtypes in Latin American women would be similar to that observed in the general population, a total N=2000 participants among the four cohorts provides adequate power (76%–99%) to detect 10% differences among surrogate marker classes, with the possible exception of the smallest classes when their pCR rates approximate 45%–55%. Assuming an accrual rate of 65 participants per month, we expect Part B of the study to be completed within approximately 30 months.

9.3 Randomization and Stratification

This study is observational and does not utilize randomized assignment of an experimental perturbation.

9.4 Primary Endpoint(s)

See Section 6.1.

9.5 Secondary Endpoint(s)

See Section 6.2.

9.6 Reporting and Exclusions

The disposition of all enrolled participants will be tabulated with respect to their study completion or premature discontinuation status. Participants who withdraw from the study before its completion will also have the primary reason for termination recorded.

Demographic and epidemiological characteristics of Latin American women will be recorded and correlated with the PAM 50 molecular profile. Demographic characteristics of participants excluded from the study will be recorded to ensure that the study protocol does not entail an unintentional systematic bias.

9.7 Evaluation of Toxicity

The specific regimens of standard-of-care neoadjuvant chemotherapy are all comprised of approved entities; their respective safety profiles are well established. Each country/site will be following reporting of standard-of-care drug toxicity per their established requirements.

9.8 Evaluation of Response to Standard Neoadjuvant Chemotherapy

pCR will be assessed according to the definition established in Section 6.3.2 and RCB will be assessed according to the definition established in Section 6.3.3.

9.9 Missing Data

In general, available data will be utilized and no overall imputation strategy is planned. Participants whose tissue sample or sampled RNA has degraded and who therefore cannot be categorized into a molecular profile class will still be treated and followed for the purposes of Part B.

9.10 Interim Analysis

Since this study is not a randomized and blinded design, strict rules surrounding interim analyses do not apply. However, interim analysis will be conducted every 6 to 12 month to monitor the accuracy and quality of data. Upon completion of enrollment and microarray assessments, a data freeze will be implemented and analyses for Part A will be conducted. Additionally, since some participants will have undergone neoadjuvant chemotherapy, surgical resection, and follow-up, preliminary early results (with censored observations) of Part B may be reported on this data freeze.

9.11 Ancillary Studies

9.11.1 Gene Expression Profiles and pCR

In a similar manner to Part B, we will examine the response rates (pCR) for the four classes of breast cancer intrinsic subtypes (Luminal A, Luminal B, Basal, and HER2-like). In addition, an examination of the concordance/discordance of the four breast cancer intrinsic subtype classes and the four surrogate marker classes with respect to pCR may be conducted.

10 STUDY MONITORING

This study is co-sponsored by NCI CGH and the research communities at the participating institutions from Argentina, Brazil, Chile, Mexico, and Uruguay.

10.1 Data Management

This study will use OpenClinica® (**https://www.openclinica.org/**) as the Clinical Data Management System (CDMS). OpenClinica® is a freely available, open source web-based software platform for managing clinical research studies. OpenClinica® is designed to support regulatory guidelines such as 21 CFR Part 11, and is built on a modern architecture using leading standards. This platform will be installed in servers physically located in each country. The study data will be accessible to the investigators according to their role in the study and per the established local guidelines and Data Sharing Plan.

The following informatics tools are proposed for use in this study:

- OpenClinica®, a web-based software platform for managing clinical research studies, has features for protocol configuration, design of case report forms (CRFs), electronic data capture (EDC), retrieval, and clinical data management.
- BSI-II™, a banking tool for biospecimen inventory, tracking, and annotation, enables researchers to track the collection, storage, annotation, quality assurance, and distribution of specimens, as well as find and request specimens with specific characteristics.

All users will be trained to use the systems and will comply with the instructions in the protocol-specific user's manual provided to the investigators. De-identified study data will be available for approved users, as outlined in the Data Sharing Plan.

10.2 Case Report Forms

Participant data will be collected using protocol-specific CRFs using standards and common data elements (CDEs). Study staff will enter data into the web-based electronic CRF (eCRF) in OpenClinica®. Instructions on how to use OpenClinica® are provided in the MOP.

10.3 Source Documents

All source documents will be maintained at the investigational sites as specified in the MOP. Source documents include participants' research charts or electronic medical records containing the source documents, including laboratory records for verification of eligibility and data to confirm molecular classification, as well as other data which will be used to enter data into the eCRFs.

10.4 US–LA CRN Monitoring

US–LA CRN (or their designee) may monitor/audit various aspects of the study per the study manual. These monitors will be given access to facilities, databases, supplies and records to review and verify data pertinent to the study.

10.5 Record Retention

Clinical records for all participants, including CRFs, all source documentation (containing evidence of study eligibility, history and physical findings, laboratory data, genetic data, results of consultations, *etc*.), as well as Institutional Review Board (IRB) records and other regulatory documentation, will be retained by the investigator in a secure storage facility in compliance with the US Health Insurance Portability and Accountability Act (HIPAA), Office of Human Research Protections (OHRP), International Conference on Harmonization (ICH)/Good Clinical Practice (GCP), and National Institutes of Health requirements, unless the standard at the site is more stringent. US–LA CRN will be notified prior to the planned destruction of any materials. The records should be accessible for inspection and copying by authorized persons of the US–LA CRN. The applicable regulatory requirements for the specific country participating in the study also apply and should be followed.

As each subject is consented and then enrolled, she will be allocated a unique study ID number. To ensure participant confidentiality and privacy, these numeric codes will substitute for personal identifiers on all paper documents, computer records, and blood sample vials. The results of the research study may be published, but subjects' names or identities will not be revealed. Records will remain confidential. To maintain confidentiality, the investigator will keep records in locked cabinet/secure area, and the results of tests will be coded to prevent association with subjects' names. It is expected that these data will be reported in scientific journals and scientific meetings. Confidentiality of subjects will be maintained in all forms of reporting. Subjects will be informed in general terms of the clinical results as soon as possible.

10.6 Clinical Trials Agreement

This is a collaborative effort co-sponsored by the US NCI CGH and the research communities at the participating institutions in Argentina, Brazil, Chile, Mexico, and Uruguay.

11 ETHICAL AND REGULATORY CONSIDERATIONS

11.1 Institutional Review Board/Ethics Committee Approval

Each country's specific regulatory and IRB requirements will be followed prior to study activation.

11.2 Informed Consent

All potential study participants will be given a copy of the IRB/Ethics Committee-approved informed consent to review. The investigator will explain all aspects of the study in lay language and answer all questions regarding the study. If the patient decides to enroll in the study, she will be asked to sign and date the informed consent document. Patients who refuse to participate or participants who withdraw from the study will be treated without prejudice.

Participants must be provided the option to allow the use of blood samples, other body fluids, and tissues obtained during testing, operative procedures, or other standard medical practices for further research purposes. All participants will complete an epidemiologic questionnaire regarding socio-economic and reproductive characteristics, family history of cancer, and factors associated with access to diagnosis and treatment of breast cancer (see Appendix B).

Prior to study initiation, the informed consent document must be reviewed and approved by the US–LA CRN and the IRB/Ethics Committee from each Organization which will implement the protocol. Any subsequent changes to the informed consent must also be approved by US–LA CRN and the IRB/Ethics Committee at each Organization.

11.3 Data Confidentiality Safeguards

Informed consent is a process where information is presented to enable persons to voluntarily decide whether or not to participate as a research subject. It is an on-going conversation between the human research subject and the researchers about the essential information about the study, which begins before consent is given and continues until the end of the subject's involvement in the research. Discussions of essential information about the research will include the studies purpose, duration, experimental procedures, alternatives, risks, and benefits, and subjects will have the opportunity to ask questions and have them answered.

The participants will sign the informed consent document prior to any procedures being done specifically for the study. The participants may withdraw consent at any time throughout the course of the study. A copy of the informed consent document will be given to the participants for their records. The researcher will document the signing of the consent form in the subject’s medical record. The rights and welfare of the participants will be protected by emphasizing to them that the quality of their medical care will not be adversely affected if they decline to participate in this study.

The subcontracts /site ICs provided a signed statement that sites were to follow their policies and procedures (PPI Policies) necessary to comply with all applicable PPI Laws either (a) in effect in the country or countries in which such information is used, accessed, accessible, transported, transmitted, stored, safeguarded, destroyed or otherwise interacted with; or (b) applicable to nationals of a country in which subcontract performs services regardless of whether the research or services are performed in that jurisdiction.

Two interoperable information systems are used in this study: OpenClinica (OC), a clinical data management system, and BSI, a specimen tracking system. Electronic data capture of microarray data, source documents, and other study materials are collected via a web-based interface with access to the systems secured utilizing passwords, role-based permissions, and the principle of least privilege to reduce risk and safeguard against malicious activity. Similarly, data protection measures are in place at each site in the collection and storage of microarray data via a de-identification mechanism in which unique system generated ID numbers are assigned to each sample, with no personally identifiable information (PII) or protected health information (PHI) affixed to data output. Access to this data is limited to data managers at each site.

11.4 Submission of Regulatory Documents

This study will be conducted in compliance with the protocol, GCP, and the applicable regulatory requirements for each country.

11.5 The Role of the US NIH/NCI

The NIH supports and oversees the MPBC study through Leidos Biomedical Research, Inc., the prime

contractor for NCI-Frederick. Leidos Biomed supported the logistic and operational aspects of the MPBC Study and performed auditing activities in 2014.

REFERENCES

1. Sotiriou, C., and Piccart, M.J. Taking gene-expression profiling to the clinic: when will molecular signatures become relevant to patient care? *Nat Rev Cancer* 7: 545-553, 2007.
2. Valero, V.V., Buzdar, A.U., and Hortobagyi, G.N. Locally Advanced Breast Cancer. *Oncologist* 1: 8-17, 1996.
3. Giordano, S.H. Update on locally advanced breast cancer. *Oncologist* 8: 521-530, 2003.
4. Lin, C., Moore, D., DeMichele, A., Ollila, D., Montgomery, L., Liu, M., Krontiras, H., Gomez, R. and Esserman, L. Detection of locally advanced breast cancer in the I-SPY TRIAL (CALGB 150007/150012, ACRIN 6657) in the interval between routine screening. *J. Clin. Oncol.* 27: abst. No.1503, 2009.
5. American Joint Committee on Cancer. Breast. *AJCC Cancer Staging Manual* 7th ed., 2009.
6. Ferlay, J., Bray, F., Pisani, P., Parkin, D.M. (2004). "GLOBALCAN 2002: Cancer Incidence, Mortality, and Prevalence Worldwide." IARC Press, Lyon, France.
7. Cazap, E., Buzaid, A.C., Garbino, C., de la Garza, J., Orlandi, F.J., Schwartsmann, G., Vallejos, C., and Guercovich, A. Breast cancer in Latin America: results of the Latin American and Caribbean Society of Medical Oncology/Breast Cancer Research Foundation expert survey. *Cancer* 113: 2359-2365, 2008.
8. Lozano-Ascencio, R., Gomez-Dantes, H., Lewis, S., Torres-Sanchez, L., and Lopez-Carrillo, L. [Breast cancer trends in Latin America and the Caribbean]. *Salud Publica Mex* 51 Suppl 2: s147-156, 2009.
9. Robles, S.C., and Galanis, E. Breast cancer in Latin America and the Caribbean. *Rev Panam Salud Publica* 11: 178-185, 2002.
10. Vona-Davis, L., and Rose, D.P. The influence of socioeconomic disparities on breast cancer tumor biology and prognosis: a review. *J Womens Health (Larchmt)* 18: 883-893, 2009.
11. Ziv, E., John, E.M., Choudhry, S., Kho, J., Lorizio, W., Perez-Stable, E.J., and Burchard, E.G. Genetic ancestry and risk factors for breast cancer among Latinas in the San Francisco Bay Area. *Cancer Epidemiol Biomarkers Prev* 15: 1878-1885, 2006.
12. Sotiriou, C., and Pusztai, L. Gene-expression signatures in breast cancer. *N Engl J Med* 360: 790-800, 2009.
13. Hennessy, B.T., Gonzalez-Angulo, A.M., and Hortobagyi, G.N. Individualization of neoadjuvant therapy for breast cancer according to molecular tumor characteristics. *Nat Clin Pract Oncol* 2: 598-599, 2005.
14. Perou, C.M., Sorlie, T., Eisen, M.B., van de Rijn, M., Jeffrey, S.S., Rees, C.A., Pollack, J.R., Ross, D.T., Johnsen, H., Akslen, L.A., Fluge, O., Pergamenschikov, A., Williams, C., Zhu, S.X., Lonning, P.E., Borresen-Dale, A.L., Brown, P.O., and Botstein, D. Molecular portraits of human breast tumours. *Nature* 406: 747-752, 2000.
15. Tamimi, R.M., Baer, H.J., Marotti, J., Galan, M., Galaburda, L., Fu, Y., Deitz, A.C., Connolly, J.L., Schnitt, S.J., Colditz, G.A., and Collins, L.C. Comparison of molecular phenotypes of ductal carcinoma in situ and invasive breast cancer. *Breast Cancer Res* 10: R67, 2008.
16. Huber, K.E., Carey, L.A., and Wazer, D.E. Breast cancer molecular subtypes in patients with locally advanced disease: impact on prognosis, patterns of recurrence, and response to therapy. *Semin Radiat Oncol* 19: 204-210, 2009.
17. Chuthapisith, S., Eremin, J.M., and Eremin, O. Predicting response to neoadjuvant chemotherapy in breast cancer: molecular imaging, systemic biomarkers and the cancer metabolome (Review). *Oncol Rep* 20: 699-703, 2008.
18. Jansen, M.P., Foekens, J.A., van Staveren, I.L., Dirkzwager-Kiel, M.M., Ritstier, K., Look, M.P., Meijer-van Gelder, M.E., Sieuwerts, A.M., Portengen, H., Dorssers, L.C., Klijn, J.G., and Berns, E.M. Molecular classification of tamoxifen-resistant breast carcinomas by gene expression profiling. *J Clin Oncol* 23: 732-740, 2005.
19. Ma, X.J., Wang, Z., Ryan, P.D., Isakoff, S.J., Barmettler, A., Fuller, A., Muir, B., Mohapatra, G., Salunga, R., Tuggle, J.T., Tran, Y., Tran, D., Tassin, A., Amon, P., Wang, W., Wang, W., Enright, E., Stecker, K., Estepa-Sabal, E., Smith, B., Younger, J., Balis, U., Michaelson, J., Bhan, A., Habin, K., Baer, T.M., Brugge, J., Haber, D.A., Erlander, M.G., and Sgroi, D.C. A two-gene expression ratio predicts clinical outcome in breast cancer patients treated with tamoxifen. *Cancer Cell* 5: 607-616, 2004.
20. Ayers, M., Symmans, W.F., Stec, J., Damokosh, A.I., Clark, E., Hess, K., Lecocke, M., Metivier, J., Booser, D., Ibrahim, N., Valero, V., Royce, M., Arun, B., Whitman, G., Ross, J., Sneige, N., Hortobagyi, G.N., and Pusztai, L. Gene expression profiles predict complete pathologic response to neoadjuvant paclitaxel and

fluorouracil, doxorubicin, and cyclophosphamide chemotherapy in breast cancer. *J Clin Oncol* 22: 2284-2293, 2004.
21. Folgueira, M.A., Carraro, D.M., Brentani, H., Patrao, D.F., Barbosa, E.M., Netto, M.M., Caldeira, J.R., Katayama, M.L., Soares, F.A., Oliveira, C.T., Reis, L.F., Kaiano, J.H., Camargo, L.P., Vencio, R.Z., Snitcovsky, I.M., Makdissi, F.B., e Silva, P.J., Goes, J.C., and Brentani, M.M. Gene expression profile associated with response to doxorubicin-based therapy in breast cancer. *Clin Cancer Res* 11: 7434-7443, 2005.
22. Hannemann, J., Oosterkamp, H.M., Bosch, C.A., Velds, A., Wessels, L.F., Loo, C., Rutgers, E.J., Rodenhuis, S., and van de Vijver, M.J. Changes in gene expression associated with response to neoadjuvant chemotherapy in breast cancer. *J Clin Oncol* 23: 3331-3342, 2005.
23. Chang, J.C., Wooten, E.C., Tsimelzon, A., Hilsenbeck, S.G., Gutierrez, M.C., Elledge, R., Mohsin, S., Osborne, C.K., Chamness, G.C., Allred, D.C., and O'Connell, P. Gene expression profiling for the prediction of therapeutic response to docetaxel in patients with breast cancer. *Lancet* 362: 362-369, 2003.
24. Iwao-Koizumi, K., Matoba, R., Ueno, N., Kim, S.J., Ando, A., Miyoshi, Y., Maeda, E., Noguchi, S., and Kato, K. Prediction of docetaxel response in human breast cancer by gene expression profiling. *J Clin Oncol* 23: 422-431, 2005.
25. Rouzier, R., Perou, C.M., Symmans, W.F., Ibrahim, N., Cristofanilli, M., Anderson, K., Hess, K.R., Stec, J., Ayers, M., Wagner, P., Morandi, P., Fan, C., Rabiul, I., Ross, J.S., Hortobagyi, G.N., and Pusztai, L. Breast cancer molecular subtypes respond differently to preoperative chemotherapy. *Clin Cancer Res* 11: 5678-5685, 2005.
26. DeMichele, A., Gimotty, P., Botbyl, J., Aplenc, R., Colligon, T., Foulkes, A.S., and Rebbeck, T.R. In response to "Drug metabolizing enzyme polymorphisms predict clinical outcome in a node-positive breast cancer cohort". *J Clin Oncol* 25: 5675-5677, 2007.
27. DeMichele, A., Dunn, L., Gimotty, P., Livasy, C., Perou, C., L., C., and Esserman, L. Loss of p27 expression and response to neoadjuvant chemotherapy: Results from the I-SPY Trial (CALGB150007/150012, ACRIN6657). *J Clin Oncol* 27: abst. No. 11091, 2009.
28. John, E.M., Miron, A., Gong, G., Phipps, A.I., Felberg, A., Li, F.P., West, D.W., and Whittemore, A.S. Prevalence of pathogenic BRCA1 mutation carriers in 5 US racial/ethnic groups. *JAMA* 298: 2869-2876, 2007.
29. Voelker, R. Breast cancer probed in Hispanic women. *JAMA* 301: 1325-1326, 2009.
30. Bear, H.D., Anderson, S., Smith, R.E., Geyer, C.E., Jr., Mamounas, E.P., Fisher, B., Brown, A.M., Robidoux, A., Margolese, R., Kahlenberg, M.S., Paik, S., Soran, A., Wickerham, D.L., and Wolmark, N. Sequential preoperative or postoperative docetaxel added to preoperative doxorubicin plus cyclophosphamide for operable breast cancer:National Surgical Adjuvant Breast and Bowel Project Protocol B-27. *J Clin Oncol* 24: 2019-2027, 2006.
31. Fisher, B., Bryant, J., Wolmark, N., Mamounas, E., Brown, A., Fisher, E.R., Wickerham, D.L., Begovic, M., DeCillis, A., Robidoux, A., Margolese, R.G., Cruz, A.B., Jr., Hoehn, J.L., Lees, A.W., Dimitrov, N.V., and Bear, H.D. Effect of preoperative chemotherapy on the outcome of women with operable breast cancer. *J Clin Oncol* 16: 2672-2685, 1998.
32. van der Hage, J.A., van de Velde, C.J., Julien, J.P., Tubiana-Hulin, M., Vandervelden, C., and Duchateau, L. Preoperative chemotherapy in primary operable breast cancer: results from the European Organization for Research and Treatment of Cancer trial 10902. *J Clin Oncol* 19: 4224-4237, 2001.
33. Polychemotherapy for early breast cancer: an overview of the randomised trials. Early Breast Cancer Trialists' Collaborative Group. *Lancet* 352: 930-942, 1998.
34. Carey, L.A., Metzger, R., Dees, E.C., Collichio, F., Sartor, C.I., Ollila, D.W., Klauber-DeMore, N., Halle, J., Sawyer, L., Moore, D.T., and Graham, M.L. American Joint Committee on Cancer tumor-node-metastasis stage after neoadjuvant chemotherapy and breast cancer outcome. *J Natl Cancer Inst* 97: 1137-1142, 2005.
35. Kuerer, H.M., Newman, L.A., Smith, T.L., Ames, F.C., Hunt, K.K., Dhingra, K., Theriault, R.L., Singh, G., Binkley, S.M., Sneige, N., Buchholz, T.A., Ross, M.I., McNeese, M.D., Buzdar, A.U., Hortobagyi, G.N., and Singletary, S.E. Clinical course of breast cancer patients with complete pathologic primary tumor and axillary lymph node response to doxorubicin-based neoadjuvant chemotherapy. *J Clin Oncol* 17: 460-469, 1999.

36. Bear, H.D., Anderson, S., Brown, A., Smith, R., Mamounas, E.P., Fisher, B., Margolese, R., Theoret, H., Soran, A., Wickerham, D.L., and Wolmark, N. The effect on tumor response of adding sequential preoperative docetaxel to preoperative doxorubicin and cyclophosphamide: preliminary results from National Surgical Adjuvant Breast and Bowel Project Protocol B-27. *J Clin Oncol* 21: 4165-4174, 2003.
37. Piccart-Gebhart, M.J., Procter, M., Leyland-Jones, B., Goldhirsch, A., Untch, M., Smith, I., Gianni, L., Baselga, J., Bell, R., Jackisch, C., Cameron, D., Dowsett, M., Barrios, C.H., Steger, G., Huang, C.S., Andersson, M., Inbar, M., Lichinitser, M., Lang, I., Nitz, U., Iwata, H., Thomssen, C., Lohrisch, C., Suter, T.M., Ruschoff, J., Suto, T., Greatorex, V., Ward, C., Straehle, C., McFadden, E., Dolci, M.S., and Gelber, R.D. Trastuzumab after adjuvant chemotherapy in HER2-positive breast cancer. *N Engl J Med* 353: 1659-1672, 2005.
38. Romond, E.H., Perez, E.A., Bryant, J., Suman, V.J., Geyer, C.E., Jr., Davidson, N.E., Tan-Chiu, E., Martino, S., Paik, S., Kaufman, P.A., Swain, S.M., Pisansky, T.M., Fehrenbacher, L., Kutteh, L.A., Vogel, V.G., Visscher, D.W., Yothers, G., Jenkins, R.B., Brown, A.M., Dakhil, S.R., Mamounas, E.P., Lingle, W.L., Klein, P.M., Ingle, J.N., and Wolmark, N. Trastuzumab plus adjuvant chemotherapy for operable HER2-positive breast cancer. *N Engl J Med* 353: 1673-1684, 2005.
39. Carey, L.A., Dees, E.C., Sawyer, L., Gatti, L., Moore, D.T., Collichio, F., Ollila, D.W., Sartor, C.I., Graham, M.L., and Perou, C.M. The triple negative paradox: primary tumor chemosensitivity of breast cancer subtypes. *Clin Cancer Res* 13: 2329-2334, 2007.
40. Guarneri, V., Broglio, K., Kau, S.W., Cristofanilli, M., Buzdar, A.U., Valero, V., Buchholz, T., Meric, F., Middleton, L., Hortobagyi, G.N., and Gonzalez-Angulo, A.M. Prognostic value of pathologic complete response after primary chemotherapy in relation to hormone receptor status and other factors. *J Clin Oncol* 24: 1037-1044, 2006.
41. Symmans, W.F., Peintinger, F., Hatzis, C., Rajan, R., Kuerer, H., Valero, V., Assad, L., Poniecka, A., Hennessy, B., Green, M., Buzdar, A.U., Singletary, S.E., Hortobagyi, G.N., and Pusztai, L. Measurement of residual breast cancer burden to predict survival after neoadjuvant chemotherapy. *J Clin Oncol* 25: 4414-4422, 2007.
42. Lester, S.C., Bose, S., Chen, Y.Y., Connolly, J.L., de Baca, M.E., Fitzgibbons, P.L., Hayes, D.F., Kleer, C., O'Malley, F.P., Page, D.L., Smith, B.L., Tan, L.K., Weaver, D.L., and Winer, E. Protocol for the examination of specimens from patients with invasive carcinoma of the breast. *Arch Pathol Lab Med* 133: 1515-1538, 2009.

REVISION HISTORY

Version	Effective Date	Description of Changes
3.2	12/6/2010	Initial release with MOP v1.0
3.3	2/24/2011	1. Updated Table of Contents and List of Abbreviations<br>2. Updated information on BSI-II, replacing caTISSUE as the specimen tracking system<br>3. Reflected changes in the informatics tools used for the MPBC Study<br>4. Replaced Data and Safety Monitoring Board (DSMB) with Data Monitoring Committee (DMC)<br>5. Revised Appendix B, the 'Epidemiology Questionnaire'<br>6. Released with MOP v2.0
3.4	12/28/2011	1. Formatted and edited the document, updated the Table of Contents, and added a Revision History<br>2. Replaced the word 'patient' with 'participant' throughout the protocol. Once a patient is enrolled in a clinical trial she/he becomes a participant.<br>3. Cover Page: Updated the names of the National Coordinators for Chile and Sonora, Mexico<br>4. Replaced 'Office of Latin American Cancer Program Development (OLACPD)' with 'Center for Global Health (CGH)' throughout the document<br>5. Study Schema: Modified Figures A and B to clarify the two arms in the study (i.e., primary surgery and neoadjuvant chemotherapy)<br>6. Added American Joint Committee on Cancer (AJCC) 7th edition to breast cancer clinical stage throughout the protocol<br>7. For clarification purposes, removed the term 'screening' from headings describing Part A of the study throughout the protocol<br>8. Section 3, Summary of Study Plan: Re-phrased select sentences for clarification<br>9. Removed the term 'pilot' used to describe the Molecular Profiling of Breast Cancer Study; this study is not a pilot<br>10. Section 4, Inclusion and Exclusion Criteria:<br>a. Inclusion Criteria of Part A (Section 4.2.1.):<br>i. Added the following sentence: 'Clarification: Participants with clinical stage II breast cancer that are later classified as histologically-confirmed stage I will remain on study; participants who are later classified as histologically-confirmed stage IV breast cancer will be taken off study.'<br>b. Exclusion Criteria of Part A (Section 4.2.2.):<br>i. Re-phrased the sentence 'Prior history of invasive cancer' to 'Prior history of non-breast malignancy (excluding in situ cancers treated only by local

Version	Effective Date	Description of Changes
		excision and basal cell and squamous cell carcinomas of the skin) within 5 years prior to enrollment in this study.'<br>ii. Added 'bilateral invasive or in-situ breast cancer'<br>iii. Moved 'inflammatory breast cancer' from exclusion criteria of Part B to this section<br>iv. Re-phrased the following sentence: 'Prior hormone therapy, chemotherapy, biologic, targeted therapies, or radiation therapy for this malignancy. Prior bisphosphonate is allowed.'<br>v. Deleted 'Use of immunomodulators and corticosteroids during the past six months'<br>c. Inclusion Criteria of Part B (Section 4.3.1):<br>i. Added 'All histological types are included.'<br>ii. Deleted 'leukocytes > 3000/ul'<br>iii. Added 'ALP ≤ 2.5 x institutional ULN'<br>iv. Modified 'Creatinine < 1.5 × institutional ULN' to 'Creatinine ≤ 1.5 × institutional ULN'<br>v. Added 'Negative serum or urine beta-HCG, unless participant is post-hysterectomy or menopausal'.<br>d. Exclusion Criteria of Part B (Section 4.3.2.):<br>i. Deleted 'metaplastic carcinoma'<br>ii. Moved inflammatory breast cancer to section 4.2.2<br>11. Section 5, Biospecimens for Biomarker, Genetic, and Genomic Analyses:<br>a. Modified sentence: 'Biopsy for research purposes will be collected at that time *or after diagnostic procedures.*'<br>b. Modified sentence: 'The pathology laboratories performing IHC, FISH, and CISH procedures will utilize commercial kits produced by the same manufacturer (*and from the same lot, if possible)*…'<br>c. Added sentence: 'In the event that both biopsy and surgical resection samples pre-chemotherapy are available from a participant, RNA will be extracted from the core biopsy sample.'<br>d. Deleted sentence: 'Serum, plasma, whole blood, and buffy coat cells will be stored at –80˚C.'<br>e. Modified sentence: 'The results of studies *with clinical applicability* will be shared with the participant *(e.g., results of BRCA1/2 testing).'*<br>f. Changed title of Section 5.2 from 'Standard of Care Treatment' to 'Neoadjuvant Chemotherapy'<br>12. Section 6, Criteria for Evaluation and Endpoint Definition:<br>a. Added 'RCB following neoadjuvant chemotherapy' to the

Version	Effective Date	Description of Changes
		'Secondary Endpoints' section (6.2)<br>b. Changed 'viable' to 'microscopically viable'<br>c. Changed 'Study Chair' to 'Investigator (participant's physician)'<br>13. Section 7, Standard Neoadjuvant Chemotherapy Administration:<br>a. Changed title of the section to 'Chemotherapy Administration'.<br>b. Changed title of section 7.1 to 'Neoadjuvant Chemotherapy Administration'.<br>c. Added Epirubicin (75 to 100 mg/m2) to the standard chemotherapeutic drugs used in this study.<br>d. Added section 7.2 'Adjuvant Chemotherapy Administration' and moved paragraph under that section.<br>14. Section 8: Clinical Evaluation and Procedures:<br>a. Schedule of Events Table has been updated to reflect the time points for response evaluation, as well as adjuvant therapy data collection. A note was added to clarify that a comprehensive list of case report forms (CRFs) to be completed for each visit is found in the *CRFs and Quality Control Forms Completion Guidelines*.<br>b. Deleted the requirement for serum beta-HCG testing every 3 weeks during neoadjuvant chemotherapy (see Schedule of Events Table).<br>c. Deleted the sentence 'Once the screening informed consent is obtained from provisionally eligible participants, the participant is enrolled in the study'.<br>d. Changed title of section 8.4. to 'Neoadjuvant Chemotherapy'.<br>e. Added a new paragraph to section 8.4: 'Participants who are enrolled on the neoadjuvant arm of the study but elect to have primary surgery will remain on the neoadjuvant arm for purposes of study analysis but will not be evaluable for response to therapy. This will be recorded on CRF Off treatment.'<br>f. Deleted 'primary' in section 8.6<br>15. Section 10: Study Monitoring<br>a. In section 10.1, removed redundant information about OpenClinica, the Clinical Data Management System proposed for the study<br>16. Released this version of the protocol with MOP v3.0
3.4.1	05/24/2013	1. Cover Page:<br>a. The name and address of the National Coordinator for Uruguay was updated.<br>b. The address of the National Coordinator for the United States was updated.

Version	Effective Date	Description of Changes
3.4.2	06/20/2014	See Appendix B for a list of changes to 3.4.2. Also note there were no changes made to the **Epidemiology Questionnaire.**

NOTE: Versions prior to v3.2 were circulated as drafts.

APPENDIX A: Performance Status Criteria

Performance Status Criteria

**ECOG Performance Status Scale**		**Karnofsky Performance Scale**	
**Grade**	**Descriptions**	**Percent**	**Description**
0	Normal activity. Fully active, able to carry on all pre-disease performance without restriction.	100	Normal, no complaints, no evidence of disease.
		90	Able to carry on normal activity; minor signs or symptoms of disease.
1	Symptoms, but ambulatory. Restricted in physically strenuous activity, but ambulatory and able to carry out work of a light or sedentary nature (*e.g.,* light housework, office work).	80	Normal activity with effort; some signs or symptoms of disease.
		70	Cares for self, unable to carry on normal activity or to do active work.
2	In bed <50% of the time. Ambulatory and capable of all self-care, but unable to carry out any work activities. Up and about more than 50% of waking hours.	60	Requires occasional assistance, but is able to care for most of his/her needs.
		50	Requires considerable assistance and frequent medical care.
3	In bed >50% of the time. Capable of only limited self-care, confined to bed or chair more than 50% of waking hours.	40	Disabled, requires special care and assistance.
		30	Severely disabled, hospitalization indicated. Death not imminent.
4	100% bedridden. Completely disabled. Cannot carry on any self-care. Totally confined to bed or chair.	20	Very sick, hospitalization indicated. Death not imminent.
		10	Moribund, fatal processes progressing rapidly.
5	Dead.	0	Dead.

APPENDIX B: List of Changes for Protocol Version 3.4.2 (September 4, 2014)

Administrative Change to the Study Protocol
The title page of the protocol; i.e., the US representative Dr. Thomas Gross was updated.

SCHEMA FIGURE A

- Figure A: Breast Cancer Study-Part A: Gene expression profiling by PAM50 assay, ***was replaced with*** *Characteristics by Immunohistochemistry: Microarray PAM50 Assay*. ***Removed*** Molecular Profiling
- Assignment to Primary Surgery ARM, ***Replaced with*** *Assignment by treating physician to Primary Surgery ARM*
- Assignment to Neoadjuvant Chemotherapy ARM, ***Replaced with*** A*ssignment by treating physician to Neoadjuvant Chemotherapy ARM*
- Histologically confirmed Stage I, II, or III
- Diagnostic tissue Gene expression profiling using the 50 gene analysis Prediction Analysis of Microarray (PAM50) Assay – ***Replaced with*** *Diagnostic tissue gene expression profiling using Microarray (PAM50) Assay*
- Diagnostic tissue Gene expression profiling using the 50 gene analysis Prediction Analysis of Microarray (PAM50) Assay – ***Replaced with*** *Diagnostic tissue gene expression profiling using Microarray (PAM50) Assay*
- Participant off Treatment Adjuvant therapy as per treating physician – **Replaced with** *Adjuvant therapy as per treating physician*

SCHEMA FIGURE B

- AC or EC q3w × 4 Cycles Followed by Paclitaxel q1w × 12 wks with Trastuzumab, ***added*** *recommended*
- AC or EC q3w ×4 Cycles Followed by Docetaxel q3w × 4 Cycles with Trastuzumab ***added*** *Recommended*
- Gene expression Molecular Profiling using the 50-gene Prediction Analysis of Microarray (PAM50) Assay, ***Replaced with*** *Gene expression profiling using the Microy (PAM50) Assay*
- Participant Off Treatment Adjuvant therapy as per treating physician, ***Replaced with*** *Adjuvant therapy as per treating physician*

1 OBJECTIVES

1.1 Primary Objective

Was revised to include: stage II or III ***by immunohistochemistry and gene expression*** profiles (luminal type A, luminal type B, human epidermal growth factor receptor 2 (HER2)-like, basal ***as determined by the 50-gene Prediction Analysis of Microarray (PAM 50) Assay***) in Latin American women.

1.2 Secondary Objectives

Bullet one: added ***gene expression***, deleted ***molecular***

Bullet two: deleted "***To estimate the rate of pathologic complete response (pCR) to standard neoadjuvant chemotherapy in each of the breast cancer molecular subtypes and to evaluate any differences in success rates among the*** ", added ***breast cancer intrinsic subtype cohort,*** deleted, ***molecular subtype cohorts***. Deleted ***"more complex and"***

Bullet four: added ***breast cancer intrinsic subtype***, deleted ***molecular subtype***

Bullet five: added ***breast cancer intrinsic subtype,*** deleted ***molecular subtype***

2.3 Molecular Profiling and Genetic Analysis of Breast Cancer

Deleted the following paragraph, #5 and #6

Paragraph #5: Starting with **"*Whole genome profiling technologies…*** and ending with **"*ER+ patients has been described [27]."***

Paragraph 6: Starting with ***"It is known that germline …*** ending with ***"*genes associated with increased risk for breast cancer"**

3 SUMMARY OF STUDY PLAN

Paragraph 1: 2nd sentence; deleted ***molecular profiles***, added ***breast cancer***, added ***immunohistochemistry***

Paragraph 2: 5th sentence; deleted ***research (e.g., genetic and genomic studies)***, added ***future correlative studies***, Inserted, starting with ***correlative studies using blood***, ending with ***and informed consent,*** added ***or a separate protocol and additional informed consent.***

6th sentence; corrected ***These***, deleted ***amendment***, inserted text starting with, ***will undergo both***, ending with ***will be re-consented***

8th sentence; deleted ***as a part of,*** added ***in addition to,***

11th sentence***;*** added ***gene expression,*** deleted ***molecular***

Paragraph 3: 1st sentence; added ***and clinical***,
3rd sentence; deleted ***per protocol***, added ***recommended in protocol***
4th sentence; deleted ***for***, inserted ***will require a protocol …*** ending with ***of patients***
5th sentence; deleted ***amendments to the protocol***… ending with ***would be developed***
6th sentence; deleted ***These amendments will undergo***… ending with ***IRB review***
7th sentence; deleted ***studies***, added ***by PAM50 assay***
12th sentence; inserted ***Decision for additional*** … ending with ***of treating physician***

4.2 Inclusion and Exclusion Criteria for Part A of the Study:
Deleted in the section title ***Molecular Profiling***

Exclusion Criteria:
Deleted ***Bullet Four: HIV-1-positive or hepatitis C-positive subjects.***

Added: **Note:** Subjects who were enrolled prior to this amendment will be considered eligible even if the HIV/Hep C and pregnancy test were not preformed due to each country standards.

4.3 Inclusion and Exclusion Criteria for Part B of the Study:
Deleted in the section title **“*Neoadjuvant Chemotherapy*”**

5.1 Part A of the Study: added ***Gene Expression,*** deleted ***Molecular,*** added ***by PAM-50 Assay***

Paragraph 3: 1st sentence; deleted, ***molecular profiles,*** added ***breast cancer***, added ***intrinsic subtypes by PAM 50 assay***
2nd sentence; added ***for***, deleted ***DNA,*** added ***RNA***, added ***microarrays***.
3rd sentence; added ***as part of the research***, change upper case ***“T”*** to lower case ***“t”***

Paragraph 4: New paragraph starting with ***RNA*** (was DNA-changed to RNA) and ending with ***the participants***
3rd sentence; added ***MOP***, deleted ***Manual of Operations***
4th sentence; ***DNA*** was changed to ***RNA***
7th sentence; deleted ***is not standard of care and***

Paragraph 5: 1st sentence; added ***at baseline***
2nd sentence; added ***or separate protocol***
3rd sentence; deleted ***these amendments***, added ***Future amendments or***, deleted ***will***, added ***undergo***,
Sentences 4, 5, 6 & 7; were deleted, starting with ***DNA***… ending with ***BRCA1/2 testing***

5.2 Part B of the Study: Neoadjuvant Chemotherapy

Paragraph 4: 1st sentence; added ***by PAM-50 assay***, and for, deleted ***including***
Paragraph 5: 2nd sentence; added ***biobanks for future*** … ending with ***would be developed***
3rd sentence; added ***These amendments*** … ending with ***patients re-consented***, deleted ***80°C for DNA extraction***… ending with ***analyses***

6.2 Secondary Endpoints
Bullet one: added ***breast cancer intrinsic subtype***, deleted ***molecular profile cohort***
Bullet two: added ***breast cancer intrinsic subtype***, deleted ***molecular profile cohort***
Bullet six: added ***breast cancer intrinsic subtype***, deleted ***molecular profile***.

8 CLINICAL AND RESEARCH EVALUATIONS AND PROCEDURES; added *AND RESEARCH*

8.1 Schedule of Events Table*

FIRST SECTION: Header: Enrollment**,** added **/Baseline**

Bolded Informed Consent and bolded Epidemiology Questionnaire

SECOND SECTION: Header, added ***IHC*** to first column, added ***X***^{c} to second column, added ***X***^{c} to the fourth column

THIRD SECTION: Header, added ***Microarrays (PAM 50 Assay,*** added ***X***^{c} to second column, added ***X***^{c} to the fourth column, added, ***per treating physician's discretion*** to first column

FOOTNOTE TO TABLE: added [2] ***On diagnostic tissue and surgical tissue for patients in Arm B,***
added ****Procedures in bold and marked with an asterisk indicate research procedures only. Refer to section 5.1 for details about number of core biopsies for diagnostic clinical purposes versus research purposes.***

8.2 Enrollment: added */Baseline*

Paragraph 1: 2ND sentence; deleted ***All participants will*** … ending with ***(see Appendix B).***

Paragraph 2: added, ***clinical***
Bullet six: deleted ***and assays to*** … ending with ***(ER, PgR, HER2, Ki67),*** deleted, ***starting with In addition***, ending with, ***US–LA CRN SOPs***.
deleted, ***a minimum of four***……ending with ***taken.***
Bullet seven: *deleted*

Paragraph 3: *added*; ***All participants…***
Bullet one: *added;* ***inserted***, ***assays to determine***
Added: ***Note: Total number of core biopsies (clinical + research) will range from four to seven.***

Bullet two: ***new,*** moved from other location
Bullet three: bullet 3 ***became*** bullet 2
Bullet four: ***added***

Paragraph 4: added, ***All;*** added, ***breast cancer and;*** deleted, starting with ***consecutively*** …through, ***study;*** added, ***assigned to the neoadjuvant chemotherapy arm***

8.3 Baseline Surgical Evaluation

Paragraph one: added, ***research***; added, ***gene expression***; deleted, ***molecular;*** added, ***clinical***

8.4 Neoadjuvant Chemotherapy

Paragraph 2: 1st Sentence; deleted, ***clinical***
Paragraph 3: deleted

8.5 Pre-surgery Evaluation after Neoadjuvant Chemotherapy

Paragraph 1: added at the end of the 7th line, ***for clinical purposes***

9.1 Study Design/Objectives

Paragraph 1: added, **The study design …parts**; added**, research**; deleted, **portion**; added, **IHC and gene expression**; deleted, **molecular**; added, **using the PAM 50 assay**

9.2 Sample Size/Accrual Rate
Paragraph 1: added, gene expression; deleted, molecular,
Font color: changed to red, ***65-94%; worst case*** *(upper triangle)*
Font color: changed to green, ***99%; best case*** *(lower triangle)*

9.6 Reporting and Exclusions

Paragraph 2: added, ***recorded and correlated …molecular profile***; deleted, ***presented …molecular profile***

9.10 Interim Analysis
Paragraph 1: 2nd sentence - added, interim analysis will … quality of data;
3rd sentence – Started new sentence, ***Upon***

9.11 Ancillary Studies
9.11.1 – added, ***Gene Expression***; deleted ***Molecular***

Paragraph 1: 1st Sentence - added, ***breast cancer intrinsic subtypes***; deleted, ***molecular profiles***;
2nd Sentence - added, ***breast cancer intrinsic subtypes***; deleted, ***molecular profiles***;

10.4 US–LA CRN Monitoring
Paragraph 1: 1st Sentence – added, ***per the study manual***

10.5 Record Retention
Paragraph 2: added, ***As each subject …results as soon as possible***; deleted, ***he or***

Added: 11.3 Data Confidentiality Safeguards – two paragraphs added

Renamed 11.3 Submission of Regulatory Documents to the above text and moved the old 11.3 to 11.4

Edited: 1st Sentence: added, ***study***; deleted, ***trial***

Added: 11.5 The Role of the US NIH/NCI

The NIH supports and oversees the MPBC study through Leidos Biomedical Research, Inc., the prime contractor for NCI-Frederick. Leidos Biomed supported the logistic and operational aspects of the MPBC Study and performed auditing activities in 2014.

List of Changes for Protocol Version 3.4.2 edits made on October 29, 2014 to address SSIRB stipulations to version 3.4.2.
Added a word to: Section 10.5: Record Retention: 'genetic data"

Clinical records for all participants, including CRFs, all source documentation (containing evidence of study eligibility, history and physical findings, laboratory data, genetic data, results of consultations, *etc*.), as well as Institutional Review Board (IRB) records and other regulatory documentation, will be retained by the investigator in a secure storage facility in compliance with the US Health Insurance Portability and Accountability Act (HIPAA), Office of Human Research Protections (OHRP), International Conference on Harmonization (ICH)/Good Clinical Practice (GCP), and National Institutes of Health requirements, unless the standard at the site is more stringent. US–LA CRN will be notified prior to the planned destruction of any materials.

Added a part to section 11.3: Data Confidentiality Safeguards:

The subcontracts /site ICs provided a signed statement that sites were to follow their policies and procedures (PPI Policies) necessary to comply with all applicable PPI Laws either (a) in effect in the country or countries in which such information is used, accessed, accessible, transported, transmitted, stored, safeguarded, destroyed or otherwise interacted with; or (b) applicable to nationals of a country in which subcontract performs services regardless of whether the research or services are performed in that jurisdiction.

APPENDIX C: MPBC Study Epidemiology Questionnaire

<u>SURVEY IDENTIFICATION</u>
Survey number: |__|__|__|__|__|

Center Name: ____|__|__|__|
Interviewer: ____|__|__|__|
Date interview started: |__|__| / |__|__| / |__|__|__|__|
Hour interview started: |__|__|:|__|__|
Date interview ended: |__|__| / |__|__| / |__|__|__|__|
Hour interview ended: |__|__|:|__|__|
Date of Data Entry: |__|__| / |__|__| / |__|__|__|__|

EP01: SOCIOECONOMIC AND DEMOGRAPHIC MODULE

1. CRF Header Information:

   <u>IDENTIFICATION OF PARTICIPANT</u>
   Participant Identification Code
   |__|__| / |__|__|__|__| / |__|__|__|__|
   Country Code/Center Code/Participant Code
   Country Code: Please refer to the lookup table.

2. How old are you?
   |__|__|__| Years

3. In what country were you born?
   ____|__|__|

4. How long have you been living in ....*?
   ****Here, each participating country will ask about the country where the study is being conducted.***
   |__|__|__| Years |__|__| Months ***(Does not know: years = 999; months = 99)***

5. In what ....* were you born?
   ****Here, each country will ask this question in terms of the provinces or States by which it is politically and administratively divided. For example, 'In what province were you born?' will be asked in Argentina while 'In what State were you born?' will be asked in Brazil.***
   ____|__|__|

6. In what city were you born?
   ____|__|__|

7. Which of the following alternatives best describes your ‘geographic ancestry’?
   Mark all answers that apply to reflect mixed ancestry (if applicable).
   1) |__| African
   2) |__| European
   3) |__| Indigenous or Native American *(e.g., Native North and Central America, Native South American, Canadian First Peoples)*
   4) |__| Southwest Asian and Middle Eastern *(e.g., Turkey, Arabia, Iran, Syria, Israel)*
   5) |__| South Asian *(e.g., Pakistan and India)*
   6) |__| East and South East Asian *(e.g., China, Japan, Korea, Vietnam, Malaysia)*
   7) |__| Pacific Islander *(e.g., Native Hawaiian, Samoan, Maori, Aboriginal Australian)*
   9) |__| Unknown/ Don’t know

7a. Are you of Jewish ancestry? (Please also select ‘Yes’ even if only one of your parents is Jewish.)
   1) |__| Yes
   2) |__| No
   9) |__| Does not know

8. What is your marital status?
   1) |__| Married or living as married
   2) |__| Separated/ divorced
   3) |__| Single
   4) |__| Widowed

9. What is your current occupation?
   Code using the table form ISCO-08, International Classification of Occupations.
   |__|__|__|__|

10. What was your occupation for most of your life?
   Code using the table form ISCO-08, International Classification of Occupations.
   |__|__|__|__|

11. How many rooms does your house have, including bathrooms and kitchen (that is, enclosed in walls and with a roof)?
   |__|__| Rooms ***(Does not know = 99)***

12. How many of these rooms in your house are permanently used for sleeping?
   |__|__| Rooms ***(Does not know = 99)***

13. How many persons usually live in your house?
   |__|__| Persons ***(Does not know = 99)***

14. In relation to public health services, to which of the following do you have access?
    a. WATER
        1) |__| Flowing/Potable Water
        2) |__| Manual Pump
        3) |__| Electric Motor
        4) |__| River
        5) |__| Pond
        6) |__| Well
        7) |__| Stream

    b. SANITATION
        1) |__| Sewer
        2) |__| Well ***(Ignore this response in English)***
        3) |__| Septic tank
        4) |__| Latrine

15. What is the total income of your family per month?
    Interviewer: Use the format that is used in your country; round to nearest whole number.
    $ |__||__||__|.|__|__|__|,|__||__| ***(Does not know = 99)***
    or
    $ |__||__||__|,|__|__|__|.|__||__| ***(Does not know = 99) (For Mexico only)***

16. A total of how many persons are financially dependent on this monthly income?
    |__|__| Persons ***(Does not know = 99)***

17a. How well do you read?
    1) |__| Very well
    2) |__| Fairly well
    3) |__| Not very well
    4) |__| Not at all (unable to read)

17b. How well do you write?
    1) |__| Very well
    2) |__| Fairly well
    3) |__| Not very well
    4) |__| Not at all (unable to write)

18. Are you currently going to school?
    1) |__| Yes
    2) |__| No

19. In total, how many years of schooling have you completed in elementary school or *EGB (General Basic Education in some Latin American school systems)*?
    |__|__|__| Years ***(Does not know = 999)***

20. In total, how many years of schooling have you completed in secondary school?
    |__|__|__| Years ***(Does not know = 999)***

21. In total, how many years of schooling have you completed in third level non-university?
    |__|__|__| Years ***(Does not know = 999)***

22. In total, how many years of schooling have you completed in the university?
    |__|__|__| Years ***(Does not know = 999)***

23. Evaluation of Socioeconomic and Demographic Module:
    1) |__| Unsatisfactory
    2) |__| Questionable
    3) |__| Reliable

EP02: MODULE FOR ACCESS TO HEALTH SERVICES

1. Do you have access to, or are you eligible for, any public health insurance, private health insurance, or work-related health insurance?
   1) |__| Yes
   2) |__| No ***(Go to 5)***

2. Regarding the coverage of office medical visits by this health insurance,
   1) |__| Health insurance pays all of the costs
   2) |__| Health insurance pays some of the costs
   3) |__| Health insurance pays none of the cost
   9) |__| Does not know

3. Regarding the coverage of complementary workup tests on additional studies by this health Insurance,
   1) |__| Health insurance pays all of the costs
   2) |__| Health insurance pays some of the costs
   3) |__| Health insurance pays none of the cost
   9) |__| Does not know

4. Regarding the coverage of cancer treatment by this health insurance,
   1) |__| Health insurance pays all of the costs
   2) |__| Health insurance pays some of the costs
   3) |__| Health insurance pays none of the cost
   9) |__| Does not know

A Papanicolaou test, also called a Pap test or Pap smear, is a procedure to test for cervical cancer in women. A Pap smear involves collecting cells from your cervix—the lower, narrow end of your uterus that is at the top of your vagina.

Note: Some women may know this test by other names, e.g., only "Papanicolaou' or 'Cytology'

5. Have you ever had a test to detect cervical cancer, that is, have you ever had a PAP test?
   1) |__| Yes
   2) |__| No ***(Go to 9)***
   9) |__| Does not know ***(Go to 9)***

6. When was the first time you had it?
   |__|__|__|__| Year ***(Does not know = 9999)***

7. How old were you when you had it done for the first time?
   |__|__|__| Years ***(Does not know = 999)***

8. In general, how often did you have a PAP test done?
   1) |__| 1 or more times per year
   2) |__| 1 time every 2 years
   3) |__| 1 time every 3 years
   4) |__| Not regularly
   9) |__| Does not know

A clinical breast examination is one in which the doctor or nurse palpates (touches) your breasts to determine any possible problem, such as cysts, nodules or other possible diseases.

Questions 9 through 25 below refer to mammograms, biopsies, and surgeries and procedures PRIOR TO OR BEFORE current diagnosis.

9. ***Before current diagnosis***, when was the first time that a doctor or nurse gave you a clinical breast examination?
   |__|__|__|__| Year
   (Does not know = 9999, and continue to Question 10; never = 8888, and go to 12)

10. ***Before current diagnosis***, how old were you the first time a doctor or nurse gave you a clinical breast examination?
   |__|__|__| Years ***(Does not know = 999)***

11. ***Before current diagnosis***, how often did you have a clinical breast examination done by a doctor or nurse?
   1) |__| 1 or more times per year
   2) |__| 1 time every 2 years
   3) |__| 1 time every 3 years
   4) |__| Not regularly
   9) |__| Does not know

A mammogram is an examination that women have done at a clinic. It is a specific radiological examination of the breasts. This examination is used to detect cysts, nodules, cancer or other breast diseases.

The information sought in the following questions is on prior screening mammograms, not mammograms used in the diagnosis of your current breast problem.

12. ***Before current diagnosis***, when was the first time in your life that you had a mammogram?
   |__|__|__|__| Year ***(Does not know = 9999, and continue to Question 13; never = 8888, and go to 19)***

13. ***Before current diagnosis***, how old were you when you had your first mammogram?
   |__|__|__| Years ***(Does not know = 999)***

14. ***Before current diagnosis***, how often did you usually have a mammogram?
1) |__| Every 6 months
2) |__| Once per year
3) |__| Once every two years
4) |__| Not at regular intervals
9) |__| Does not know

15. ***Before current diagnosis***, when did you have your last mammogram?
|__|__|__|__| Year ***(Does not know = 9999)***

16. ***Before current diagnosis***, how old were you when you had your last mammogram?
|__|__|__| Years ***(Does not know = 999)***

17. ***Before current diagnosis***, how many of your mammograms were reported as abnormal?
|__|__| Mammograms ***(Does not know = 99)***

18. ***Before current diagnosis***, how many mammograms have you had in total?
|__|__| Mammograms ***(Does not know = 99)***

Note: Even though the information on prior surgeries may or may not be available in medical records, ask Questions 19-25 of ALL participants.

Note: Do not record information on prior biopsies here (i.e., questions 19-22); please record biopsy information below in questions 23-25.

19. ***Before current diagnosis***, have you had any breast surgery, for any reason, including removal of any nodule or cyst or breast augmentation/reduction?
1) |__| Yes
2) |__| No ***(Go to 23)***
9) |__| Does not know ***(Go to 23)***

20. ***Before current diagnosis***, how many breast surgeries have you had?
|__|__| Surgeries

21. ***Before current diagnosis,*** how old were you when you had the ....?		22. ***Before current diagnosis***, what was done in this surgery? ***Interviewer: More than one choice can be marked as applicable.***	
**1st Surgery**	|___|___|___| Years ***(Does not know = 999)*** or Year: |___|___|___|___| ***(Does not know = 9999)***	1. Remove cysts/nodules or other benign lesions<br>2. Breast augmentation<br>3. Breast reduction<br>4. Other (Specify:_________________________)<br>9. Does not know	☐<br>☐<br>☐<br>☐<br>☐
**2nd Surgery**	|___|___|___| Years ***(Does not know = 999)*** or Year: |___|___|___|___| ***(Does not know = 9999)***	1. Remove cysts/nodules or other benign lesions<br>2. Breast augmentation<br>3. Breast reduction<br>4. Other (Specify:_________________________)<br>9. Does not know	☐<br>☐<br>☐<br>☐<br>☐
**3rd Surgery**	|___|___|___| Years ***(Does not know = 999)*** or Year: |___|___|___|___| ***(Does not know = 9999)***	1. Remove cysts/nodules or other benign lesions<br>2. Breast augmentation<br>3. Breast reduction<br>4. Other (Specify:_________________________)<br>9. Does not know	☐<br>☐<br>☐<br>☐<br>☐

A breast biopsy is the removal of a sample of breast tissue to determine if the suspicious lesion is malignant (cancer) or benign (non-cancerous lesion).

23. Before the biopsy that confirmed your recent diagnosis, had you ever had a breast biopsy?
    1) |__| Yes
    2) |__| No ***(Go to 26)***
    9) |__| Does not know

24. ***Before current diagnosis***, how many breast biopsies have you had before presenting with the breast disease that now brings you in for medical care?
    |__|__| Biopsies ***(Does not know = 99)***

25. ***Before current diagnosis***, what was discovered in these biopsies?
Interviewer: Mark all that apply. If unknown, mark 'Does not know' and leave the rest blank.

**a) 1st Biopsy**	1) |__| Cyst<br>2) |__| Benign tumor<br>3) |__| Normal tissue (no problem)<br>4) |__| Malignant tumor<br>5) |__| Other (Specify):____________________________<br>9) |__| Does not know
**b) 2nd Biopsy**	1) |__| Cyst<br>2) |__| Benign tumor<br>3) |__| Normal tissue (no problem)<br>4) |__| Malignant tumor<br>5) |__| Other (Specify):____________________________<br>9) |__| Does not know
**c) 3rd Biopsy**	1) |__| Cyst<br>2) |__| Benign tumor<br>3) |__| Normal tissue (no problem)<br>4) |__| Malignant tumor<br>5) |__| Other (Specify):____________________________<br>9) |__| Does not know
**d) 4th Biopsy**	1) |__| Cyst<br>2) |__| Benign tumor<br>3) |__| Normal tissue (no problem)<br>4) |__| Malignant tumor<br>5) |__| Other (Specify):____________________________<br>9) |__| Does not know

Now, we will discuss your current disease for which you came to this center for treatment.

26. How did you first discover this ***current*** problem in your breast?
   1) |__| You, yourself found this change in your breast. ***(Go to 27)***
   2) |__| Your partner or other person discovered the change in your breast. ***(Go to 27)***
   3) |__| A doctor or other health professional discovered a change in your breast during a clinical examination. ***(Go to 27)***
   4) |__| Mammogram or another imaging study of the breast ***(Go to 27)***
   5) |__| Other ***(Go to 29)***
   9) |__| Does not remember ***(Go to 29)***

27. What change(s) did you (or your partner/ someone else) discover in your breast?	28. When was the first time you (or your partner/ someone else) felt/discovered this change? ***Interviewer: Does not know: 99/9999, go to 29.***
1) |__| Nodule/cyst	|__|__| Month |__|__|__|__| Year
2) |__| Bloody or watery nipple secretion	|__|__| Month |__|__|__|__| Year
3) |__| Breast pain	|__|__| Month |__|__|__|__| Year
4) |__| Other(s):	
4.1) What ___________________________	|__|__| Month |__|__|__|__| Year
4.2) What ___________________________	|__|__| Month |__|__|__|__| Year
4.3) What___________________________	|__|__| Month |__|__|__|__| Year

29. When was the first time you went to see a doctor about this change or after this mammogram (in the case of a positive screening mammogram, but no symptoms)?
|__|__| Month |__|__|__|__| Year **<DATE OF FIRST CONSULTATION>**
(Does not know Month = 99, does not know Year = 9999)

30. Where was this consultation?
****Here, each country will be able to adapt the questions that correspond to its health system.***
1) |__| Health post or center
2) |__| Private/prepaid medical office
3) |__| Emergency center
4) |__| Public hospital
5) |__| Home visit
6) |__| Other (Specify): ____
9) |__| Does not know

31. This health center where you received care was:
****Here, each country will be able to adapt the questions that correspond to its Health system.***
1) |__| Public
2) |__| Private/Health plan
3) |__| Charity/Foundation
9) |__| Does not know

32. How much time does it take you to go from your house to the place where you had the consultation?
Interviewer: Indicate duration in hours and minutes; if <1 hour, enter 00 hours followed by appropriate minutes.
|__|__| Hours |__|__| Minutes
(Does not know Hours = 99, does not know Minutes = 9999)

33. Was the cost of this consultation covered, even in part, by any public health insurance, private health insurance, or work-related health insurance?
   1) |__| Yes
   2) |__| No
   9) |__| Does not know

34. Did you pay for none, some, or all of this consultation from your own pocket?
   1) |__| None
   2) |__| Some
   3) |__| All
   9) |__| Does not know

35. Were you able to have a mammogram the first time you contacted the health service?
   1) |__| Yes
   2) |__| No
   9) |__| Does not know
   4) |__| Not applicable because problem was detected with a screening mammogram and this test was not repeated. ***(Go to 43)***

36. How long after this first consultation did you have the mammogram done?
   |__|__| Months |__|__| Days **<THIS REFERS TO THE TIME BETWEEN THE CONSULTATION AND THE FIRST MAMMOGRAM THAT HELPED TO ESTABLISH A DIAGNOSIS>**
   (Does not know Month = 99, does not know Days = 99)

37. When was this mammogram done?
   |__|__| Month |__|__|__|__| Year **<MONTH AND YEAR OF FIRST DIAGNOSTICMAMMOGRAM>**
   (Does not know Month = 99, does not know Year = 9999)

38. In which health service did you have this first mammogram?
   ****Here, each country will be able to adapt the questions that correspond to its health system.***
   1) |__| Health post or center
   2) |__| Private clinic
   3) |__| Laboratory
   4) |__| Public hospital
   5) |__| Mobile mammography imaging center
   9) |__| Does not know

39. The place where you had this mammogram was:
   ****Here, each country will be able to adapt the questions that correspond to its health system.***
   1) |__| Public
   2) |__| Private/ Health plan
   3) |__| Charity/ Foundation
   9) |__| Does not know

40. Was the cost of this mammogram covered, even in part, by any public health insurance, private health insurance, or work-related health insurance?
    1) |__| Yes
    2) |__| No
    9) |__| Does not know

41. Did you pay for none, some, or all of this mammogram from your own pocket?
    1) |__| None
    2) |__| Some
    3) |__| All
    9) |__| Does not know

42. How much time does it take you to go from your house to the place where the mammogram was done?
    Interviewer: Indicate duration in hours and minutes; if <1 hour, enter 00 hours followed by appropriate minutes.
    |__|__| Hours |__|__| Minutes
    (Does not know Hours = 99, does not know Minutes = 9999)

Diagnostic Delay: Time from Mammogram to Biopsy or Surgery

43. When did you have the last mammogram before the biopsy or surgery that confirmed your current diagnosis?
    |__|__| Month |__|__|__|__| Year
    (Does not know Month = 99, does not know Year = 9999; Not applicable [i.e., either no mammogram or no biopsy], Month = 88 ; Year = 8888)

The questions below (marked with **) should be asked of all participants except those who did not receive a biopsy (in which case the questions do not apply).

44. **When did you have the biopsy done that confirmed your current diagnosis?
    |__|__| Month |__|__|__|__| Year
    (Does not know Month = 99, does not know Year = 9999, Never = 8888; if 'Never', go to 54)

45. **How many days did you have to wait to receive a biopsy?
    |__|__|__| Days ***(Does not know Days = 999)***

46. **Your biopsy was done at which of the following services?
    ****Here, each country will be able to adapt the questions that correspond to its health system.***
    1) |__| Health post or center
    2) |__| Private clinic
    3) |__| Laboratory
    4) |__| Public hospital
    5) |__| Other (Specify):____
    9) |__| Does not know

47. **The place where your biopsy was done was:
    Here, each country will be able to adapt the questions that correspond to its health system.
    1) |__| Public
    2) |__| Private/ Health plan
    3) |__| Charity/ Foundation
    9) |__| Does not know

48. **Was the cost of this biopsy covered, even in part, by any public health insurance, private health insurance, or work-related health insurance?
    1) |__| Yes
    2) |__| No
    9) |__| Does not know

49. **Did you pay any money directly from your pocket for this biopsy?
    1) |__| None
    2) |__| Some
    3) |__| All
    9) |__| Does not know

50. **How much time does it take you to go from your house to the place where the biopsy was done?
    Interviewer: Indicate duration in hours and minutes; if <1 hour, enter 00 hours followed by appropriate minutes.
    |__|__| Hours |__|__| Minutes
    (Does not know Hours = 99, does not know Minutes = 9999)

51. **When did you receive the result of this biopsy?
    |__|__| Month |__|__|__|__| Year
    (Does not know Month = 99, does not know Year = 9999)

52. **How long after the biopsy was done did you receive the result?
    |__|__| Months |__|__| Days
    (Does not know Months = 99, does not know Days = 99)

53. When did you receive instructions to come to this center for treatment for your current diagnosis?
    |__|__| Month |__|__|__|__| Year **<DATE OF LAST CONSULTATION>**
    (Does not know Month = 99, does not know Year = 9999)

54. Evaluation of Access to Health Module
    1) |__| Unsatisfactory
    2) |__| Questionable
    3) |__| Reliable

EP03: CANCER HISTORY MODULE OF PARTICIPANT AND FAMILY
(PARENTS' AND GRANDPARENTS' CANCER HISTORY)

The following questions are about the participant's past and present relatives and family members and if they have or have had cancer. Please include only relatives related by blood. Do not include adoptive parents, grandparents, siblings, or children.

1. Were ***you*** born from a multiple birth?
   1) |__| Yes
   2) |__| No ***(Go to 5)***
   9) |__| Does not know ***(Go to 5)***

2. In total, how many babies were born from this pregnancy? Also take into account those stillborn.
   |__|__| Babies ***(Does not know = 99)***

3. Of these siblings, how many were identical to you?
   |__|__| Identical ***(Does not know = 99)***
   Do not forget to take into account those stillborn.

4. Of these siblings, how many were not identical to you (non-identical twins)?
   |__|__| Non-identical ***(Does not know = 99)***
   Do not forget to take into account those stillborn.

5. Are you adopted?
   1) |__| Yes
   2) |__| No
   9) |__| Does not know

6. Do you know the family history of your biological parents?
   1) |__| Yes, both mother and father
   2) |__| Yes, only mother
   3) |__| Yes, only father
   9) |__| Does not know ***(Go to 24*)***

7. Are your parents consanguinous, that is, are they related by blood ?
   1) |__| Yes
   2) |__| No
   9) |__| Does not know

The following questions are about your ***biological*** mother.

8. Is your biological mother still alive?
   1) |__| Yes
   2) |__| No
   9) |__| Does not know ***(Go to 10)***

9. How old is she now or how old was she when she died?
   |__|__|__| Years ***(Does not know = 999)***

10. In what country was she born?
    Country Code: Please refer to the lookup table.
    ______________________________________ |___|___| Country code ***(Does not know = 99)***

11. Which of the following alternatives best describes your mother's 'geographic ancestry'?
    Mark all answers that apply to reflect mixed ancestry (if applicable).
    1) |__| African
    2) |__| European
    3) |__| Indigenous or Native American *(e.g., Native North and Central America, Native South American, Canadian First Peoples)*
    4) |__| Southwest Asian and Middle Eastern *(e.g., Turkey, Arabia, Iran, Syria, Israel)*
    5) |__| South Asian *(e.g., Pakistan and India)*
    6) |__| East and South East Asian *(e.g., China, Japan, Korea, Vietnam, Malaysia)*
    7) |__| Pacific Islander *(e.g., Native Hawaiian, Samoan, Maori, Aboriginal Australian)*
    9) |__| Unknown/ Don't know

12. Does/did she have any malignant tumor or cancer diagnosed by a doctor?
    1) |__| Yes
    2) |__| No ***(Go to 16)***
    9) |__| Does not know ***(Go to 16)***

13. How many diagnoses of cancer or malignant tumor has she had?
    |__|__| Diagnoses ***(Does not know = 99)***

	14. How old was she when the cancer was diagnosed? ***Interviewer: If age = 999, ask in what year the cancer was diagnosed.***	15. What type of cancer did she have?
1st Cancer	|__|__|__|Years ***(Does not know = 999)***<br>or<br>|__|__|__|__| Year diagnosed ***(Does not know = 9999)***	|__|__|__| Code
2nd Cancer	|__|__|__| Years ***(Does not know = 999)***<br>or<br>|__|__|__|__| Year diagnosed ***(Does not know = 9999)***	|__|__|__| Code
3rd Cancer	|__|__|__| Years ***(Does not know = 999)***<br>or<br>|__|__|__|__| Year diagnosed ***(Does not know = 9999)***	|__|__|__| Code

Cancer Codes: Please refer to the cancer code lookup list.

The following questions are about your ***biological*** father:

16. Is your biological father still alive?
    1) |__| Yes
    2) |__| No
    9) |__| Does not know ***(Go to 18)***
17. How old is he now or how old was he when he died?
    |__|__|__| Years ***(Does not know = 999)***

18. In what country was he born?
    Country Code: Please refer to the lookup table.
    ____|__|__| Country code ***(Does not know = 99)***

19. Which of the following alternatives best describes your father's 'geographic ancestry'?
    Mark all answers that apply to reflect mixed ancestry (if applicable).
    1) |__| African
    2) |__| European
    3) |__| Indigenous or Native American *(e.g., Native North and Central America, Native South American, Canadian First Peoples)*
    4) |__| Southwest Asian and Middle Eastern *(e.g., Turkey, Arabia, Iran, Syria, Israel)*
    5) |__| South Asian *(e.g., Pakistan and India)*
    6) |__| East and South East Asian *(e.g., China, Japan, Korea, Vietnam, Malaysia)*
    7) |__| Pacific Islander *(e.g., Native Hawaiian, Samoan, Maori, Aboriginal Australian)*
    9) |__| Unknown/ Don't know

20. Does/did he have any malignant tumor or cancer diagnosed by a doctor?
    1) |__| Yes
    2) |__| No ***(Go to 24)***
    9) |__| Does not know ***(Go to 24)***

21. How many diagnoses of cancer or malignant tumor did he have?
    |__|__| Diagnoses ***(Does not know = 99)***

	22. How old was he when the cancer was diagnosed? ***Interviewer: If age = 999, ask in what year he was diagnosed.***	23. What type of cancer did he have?
1st Cancer	|__|__|__|Years ***(Does not know = 999)***<br>or<br>|__|__|__|__| Year diagnosed ***(Does not know = 9999)***	|__|__|__| Code
2nd Cancer	|__|__|__|Years ***(Does not know = 999)***<br>or<br>|__|__|__|__| Year diagnosed ***(Does not know = 9999)***	|__|__|__| Code
3rd Cancer	|__|__|__|Years ***(Does not know = 999)***<br>or<br>|__|__|__|__| Year diagnosed ***(Does not know = 9999)***	|__|__|__| Code

Cancer Codes: Please refer to the cancer code lookup list.

Now, I would like to know about YOUR grandparents. I will start with your maternal grandparents (your mother's mother and father), and then I will ask you about your paternal grandparents (your father's mother and father).

The following questions are about your ***maternal*** grandmother:

24. Is your maternal grandmother alive?
    1) |__| Yes
    2) |__| No
    9) |__| Does not know ***(Go to 26)***

25. How old is she now or how old was she when she died?
    |__|__|__| Years ***(Does not know = 999)***

26. In what country was she born?
    Country Code: Please refer to the lookup table.
    ______________________________________|__|__| Country code ***(Does not know = 99)***

27. Which of the following alternatives best describes your maternal grandmother's 'geographic ancestry'?
    Mark all answers that apply to reflect mixed ancestry (if applicable).
    1) |__| African
    2) |__| European
    3) |__| Indigenous or Native American *(e.g., Native North and Central America, Native South American, Canadian First Peoples)*
    4) |__| Southwest Asian and Middle Eastern (e.g., Turkey, Arabia, Iran, Syria, Israel)
    5) |__| South Asian *(e.g., Pakistan and India)*
    6) |__| East and South East Asian *(e.g., China, Japan, Korea, Vietnam, Malaysia)*
    7) |__| Pacific Islander *(e.g., Native Hawaiian, Samoan, Maori, Aboriginal Australian)*
    9) |__| Unknown/ Don't know

28. Does/has she had any cancer or malignant tumor diagnosed by a doctor?
    1) |__| Yes
    2) |__| No ***(Go to 32)***
    9) |__| Does not know ***(Go to 32)***

29. How many diagnoses of cancer or malignant tumor did she have?
    |__|__| Diagnoses ***(Does not know = 99)***

	30. How old was she when the cancer was diagnosed? ***Interviewer: If age = 999, ask in what year the cancer was diagnosed.***	31. What type of cancer did she have?
1st Cancer	|__|__|__|Years ***(Does not know = 999)***<br>or<br>|__|__|__|__| Year diagnosed ***(Does not know = 9999)***	|__|__|__| Code
2nd Cancer	|__|__|__|Years ***(Does not know = 999)***<br>or<br>|__|__|__|__| Year diagnosed ***(Does not know = 9999)***	|__|__|__| Code
3rd Cancer	|__|__|__|Years ***(Does not know = 999)***<br>or<br>|__|__|__|__| Year diagnosed ***(Does not know = 9999)***	|__|__|__| Code

Cancer Codes: Please refer to the cancer code lookup list.

The following questions are about your ***maternal*** grandfather:

32. Is your maternal grandfather alive?
    1) |__| Yes
    2) |__| No
    9) |__| Does not know ***(Go to 34)***

33. How old is he now or how old was he when he died?
    |__|__|__| Years ***(Does not know = 999)***

34. In what country was he born?
    Country Code: Please refer to the lookup table.
    ____|__|__| Country code ***(Does not know = 99)***

35. Which of the following alternatives best describes your maternal grandfather's 'geographic ancestry'?
    Mark all answers that apply to reflect mixed ancestry (if applicable).
    1) |__| African
    2) |__| European
    3) |__| Indigenous or Native American *(e.g., Native North and Central America, Native South American, Canadian First Peoples)*
    4) |__| Southwest Asian and Middle Eastern *(e.g., Turkey, Arabia, Iran, Syria, Israel)*
    5) |__| South Asian *(e.g., Pakistan and India)*
    6) |__| East and South East Asian *(e.g., China, Japan, Korea, Vietnam, Malaysia)*
    7) |__| Pacific Islander *(e.g., Native Hawaiian, Samoan, Maori, Aboriginal Australian)*
    9) |__| Unknown/ Don't know

36. Did he have any cancer or malignant tumor diagnosed by a doctor?
    1) |__| Yes
    2) |__| No ***(Go to 40)***
    9) |__| Does not know ***(Go to 40)***

37. How many diagnoses of cancer or malignant tumor did he have?
|__|__| Diagnoses ***(Does not know = 99)***

	38. How old was he when the cancer was diagnosed? ***Interviewer: If age = 999, ask in what year the cancer was diagnosed.***	39. What type of cancer did he have?
1st Cancer	|__|__|__|Years ***(Does not know = 999)***<br>or<br>|__|__|__|__| Year diagnosed ***(Does not know = 9999)***	|__|__|__| Code
2nd Cancer	|__|__|__|Years ***(Does not know = 999)***<br>or<br>|__|__|__|__| Year diagnosed ***(Does not know = 9999)***	|__|__|__| Code
3rd Cancer	|__|__|__|Years ***(Does not know = 999)***<br>or<br>|__|__|__|__| Year diagnosed ***(Does not know = 9999)***	|__|__|__| Code

Cancer Codes: Please refer to the cancer code lookup list.

The following questions are about your ***paternal*** grandmother:

40. Is your paternal grandmother alive?
1) |__| Yes
2) |__| No
9) |__| Does not know ***(Go to 42)***

41. How old is she now or how old was she when she died?
|__|__|__| Years ***(Does not know = 999)***

42. In what country was she born?
Country Code: Please refer to the lookup table.
____|__|__| Country code ***(Does not know = 99)***

43. Which of the following alternatives best describes your paternal grandmother's 'geographic ancestry'?
Mark all answers that apply to reflect mixed ancestry (if applicable).
1) |__| African
2) |__| European
3) |__| Indigenous or Native American *(e.g., Native North and Central America, Native South American, Canadian First Peoples)*
4) |__| Southwest Asian and Middle Eastern *(e.g., Turkey, Arabia, Iran, Syria, Israel)*
5) |__| South Asian *(e.g., Pakistan and India)*
6) |__| East and South East Asian *(e.g., China, Japan, Korea, Vietnam, Malaysia)*
7) |__| Pacific Islander *(e.g., Native Hawaiian, Samoan, Maori, Aboriginal Australian)*
9) |__| Unknown/ Don't know

44. Does/did she have any cancer or malignant tumor diagnosed by a doctor?
    1) |__| Yes
    2) |__| No ***(Go to 48)***
    9) |__| Does not know ***(Go to 48)***

45. How may diagnoses of cancer or malignant tumor did she have?
    |__|__| Diagnoses ***(Does not know = 99)***

	46. How old was she when the cancer was diagnosed? ***Interviewer: If age = 999, ask then what year the cancer was diagnosed.***	47. What type of cancer did she have?
1st Cancer	|__|__|__|Years ***(Does not know = 999)***<br>or<br>|__|__|__|__| Year diagnosed ***(Does not know = 9999)***	|__|__|__| Code
2nd Cancer	|__|__|__|Years ***(Does not know = 999)***<br>or<br>|__|__|__|__| Year diagnosed ***(Does not know = 9999)***	|__|__|__| Code
3rd Cancer	|__|__|__|Years ***(Does not know = 999)***<br>or<br>|__|__|__|__| Year diagnosed ***(Does not know = 9999)***	|__|__|__| Code

Cancer Codes: Please refer to the cancer code lookup list.

The following questions are about your ***paternal*** grandfather:

48. Is your paternal grandfather alive?
    1) |__| Yes
    2) |__| No
    9) |__| Does not know ***(Go to 50)***

49. How old is he now or how old was he when he died?
    |__|__|__| Years ***(Does not know = 999)***

50. In what country was he born?
    Country Code: Please refer to the lookup table.
    ____|__|__| Country code ***(Does not know = 99)***

51. Which of the following alternatives best describes your paternal grandfather's 'geographic ancestry'?
Mark all answers that apply to reflect mixed ancestry (if applicable).
   1) |__| African
   2) |__| European
   3) |__| Indigenous or Native American *(e.g., Native North and Central America, Native South American, Canadian First Peoples)*
   4) |__| Southwest Asian and Middle Eastern *(e.g., Turkey, Arabia, Iran, Syria, Israel)*
   5) |__| South Asian *(e.g., Pakistan and India)*
   6) |__| East and South East Asian *(e.g., China, Japan, Korea, Vietnam, Malaysia)*
   7) |__| Pacific Islander *(e.g., Native Hawaiian, Samoan, Maori, Aboriginal Australian)*
   9) |__| Unknown/ Don't know

52. Does/did he have any cancer or malignant tumor diagnosed by a doctor?
   1) |__| Yes
   2) |__| No ***(Go to 56)***
   9) |__| Does not know ***(Go to 56)***

53. How many diagnoses of cancer or malignant tumor did he have?
   |__|__| Diagnoses ***(Does not know = 99)***

	54. How old was he when the cancer was diagnosed? ***Interviewer: If age = 999, ask in what year the cancer was diagnosed.***	55. What type of cancer did he have?
1st Cancer	|__|__|__|Years ***(Does not know = 999)***<br>or<br>|__|__|__|__| Year diagnosed ***(Does not know = 9999)***	|__|__|__| Code
2nd Cancer	|__|__|__|Years ***(Does not know = 999)***<br>or<br>|__|__|__|__| Year diagnosed ***(Does not know = 9999)***	|__|__|__| Code
3rd Cancer	|__|__|__|Years ***(Does not know = 999)***<br>or<br>|__|__|__|__| Year diagnosed ***(Does not know = 9999)***	|__|__|__| Code

Cancer Codes: Please refer to the cancer code lookup list.

EP04: CANCER HISTORY MODULE OF PARTICIPANT AND FAMILY (SIBLINGS' AND CHILDREN'S FAMILY HISTORY)

Now, I would like to know about YOUR brothers and sisters. I will start with your brothers and sisters who are related to you by both your mother and father, and then I will ask you about your half brothers and sisters (related to you by either your mother or father).

The following questions are about your ***biological*** brothers and sisters:

56. How many brothers and sisters do/did you have? Please DO NOT include half-brothers or half-sisters.
    Interviewer: If no brothers or sisters, record 00 and go to 63.
    If unknown, record 99; continue with questions for those siblings who are known to the participant.
    |__|__| Sisters
    |__|__| Brothers

Starting with your *oldest* brother/sister, please answer the following questions:

57. Gender?	58. Living?	59. How old is he/she now or how old was he/she when he/she died? ***(Do not know = 999)***	60. Does/did he/she have cancer?	61. What type(s) of cancer does/did he/she have? ***Interviewer: In case he/she has/had more than one type of cancer, please list all.***	62. At what age was the cancer diagnosed? ***(Do not know = 999)***
1) |__| Female<br>2) |__| Male	1) |__| Living<br>2) |__|Deceased<br>9) |__| Does not know	|__|__|__| Years	1) |__| Yes<br>2) |__| No ***(Go to next row)***<br>9) |__| Does not know ***(Go to next row)***	|__|__|__| Cod. Ca1<br>|__|__|__| Cod. Ca2<br>|__|__|__| Cod. Ca3	|__|__|__| Years Ca1<br>|__|__|__| Years Ca2<br>|__|__|__| Years Ca3
1) |__| Female<br>2) |__| Male	1) |__| Living<br>2) |__|Deceased<br>9) |__| Does not know	|__|__|__| Years	1) |__| Yes<br>2) |__| No***(Go to next row)***<br>9) |__| Does not know ***(Go to next row)***	|__|__|__| Cod. Ca1<br>|__|__|__| Cod. Ca2<br>|__|__|__| Cod. Ca3	|__|__|__| Years Ca1<br>|__|__|__| Years Ca2<br>|__|__|__| Years Ca3
1) |__| Female<br>2) |__| Male	1) |__| Living<br>2) |__|Deceased<br>9) |__| Does not know	|__|__|__| Years	1) |__| Yes<br>2) |__| No ***(Go to next row)***<br>9) |__| Does not know ***(Go to next row)***	|__|__|__| Cod. Ca1<br>|__|__|__| Cod. Ca2<br>|__|__|__| Cod. Ca3	|__|__|__| Years Ca1<br>|__|__|__| Years Ca2<br>|__|__|__| Years Ca3
1) |__| Female<br>2) |__| Male	1) |__| Living<br>2) |__|Deceased<br>9) |__| Does not know	|__|__|__| Years	1) |__| Yes<br>2) |__| No ***(Go to next row)***<br>9) |__| Does not know ***(Go to next row)***	|__|__|__| Cod. Ca1<br>|__|__|__| Cod. Ca2<br>|__|__|__| Cod. Ca3	|__|__|__| Years Ca1<br>|__|__|__| Years Ca2<br>|__|__|__| Years Ca3
1) |__| Female<br>2) |__| Male	1) |__| Living<br>2) |__|Deceased<br>9) |__| Does not know	|__|__|__| Years	1) |__| Yes<br>2) |__| No ***(Go to next row)***<br>9) |__| Does not know ***(Go to next row)***	|__|__|__| Cod. Ca1<br>|__|__|__| Cod. Ca2<br>|__|__|__| Cod. Ca3	|__|__|__| Years Ca1<br>|__|__|__| Years Ca2<br>|__|__|__| Years Ca3
1) |__| Female<br>2) |__| Male	1) |__| Living<br>2) |__|Deceased<br>9) |__| Does not know	|__|__|__| Years	1) |__| Yes<br>2) |__| No ***(Go to next row)***<br>9) |__| Does not know ***(Go to next row)***	|__|__|__| Cod. Ca1<br>|__|__|__| Cod. Ca2<br>|__|__|__| Cod. Ca3	|__|__|__| Years Ca1<br>|__|__|__| Years Ca2<br>|__|__|__| Years Ca3
1) |__| Female<br>2) |__| Male	1) |__| Living<br>2) |__|Deceased<br>9) |__| Does not know	|__|__|__| Years	1) |__| Yes<br>2) |__| No<br>9) |__| Does not know	|__|__|__| Cod. Ca1<br>|__|__|__| Cod. Ca2<br>|__|__|__| Cod. Ca3	|__|__|__| Years Ca1<br>|__|__|__| Years Ca2<br>|__|__|__| Years Ca3

Cancer Codes: Please refer to the cancer code lookup list.

The following questions are about your ***half-brothers*** *and* ***half-sisters***:

63. How many half-brothers and half-sisters do/did you have?
Interviewer: If no half-brothers or sisters, record 00 and go to 71.
If unknown, record 99; continue with questions for those half-siblings who are known to the participant.
|__|__| Half-sisters
|__|__| Half-brothers

Starting with your *oldest* half-brother/sister, please answer the following questions:

64. What is your relation to this half-sibling?	65. Gender?	66. Living?	67. How old is he/she now or how old was he/she when he/she died? ***(Do not know = 999)***	68. Did he/she have cancer?	69. What type(s) of cancer does/did he/she have? ***Interviewer: In case he/she has/had more than one type of cancer, please list all.***	70. At what age was the cancer diagnosed? ***(Do not know = 999)***
1) |__| Maternal<br>2) |__| Paternal	1) |__| Female<br>2) |__| Male	1) |__| Living<br>2) |__| Deceased<br>3) |__| Does not know	|__|__|__| Years	1) |__| Yes<br>2) |__| No***(Go to next row)***<br>3) |__| Does not know ***(Go to next row)***	|__|__|__| Cod. Ca1<br>|__|__|__| Cod. Ca2<br>|__|__|__| Cod. Ca3	|__|__|__| Years Ca1<br>|__|__|__| Years Ca2<br>|__|__|__| Years Ca3
1) |__| Maternal<br>2) |__| Paternal	1) |__| Female<br>2) |__| Male	1) |__| Living<br>2) |__| Deceased<br>3) |__| Does not know	|__|__|__| Years	1) |__| Yes<br>2) |__| No ***(Go to next row)***<br>3) |__| Does not know ***(Go to next row)***	|__|__|__| Cod. Ca1<br>|__|__|__| Cod. Ca2<br>|__|__|__| Cod. Ca3	|__|__|__| Years Ca1<br>|__|__|__| Years Ca2<br>|__|__|__| Years Ca3
1) |__| Maternal<br>2) |__| Paternal	1) |__| Female<br>2) |__| Male	1) |__| Living<br>2) |__| Deceased<br>3) |__| Does not know	|__|__|__| Years	1) |__| Yes<br>2) |__| No ***(Go to next row)***<br>3) |__| Does not know ***(Go to next row)***	|__|__|__| Cod. Ca1<br>|__|__|__| Cod. Ca2<br>|__|__|__| Cod. Ca3	|__|__|__| Years Ca1<br>|__|__|__| Years Ca2<br>|__|__|__| Years Ca3
1) |__| Maternal<br>2) |__| Paternal	1) |__| Female<br>2) |__| Male	1) |__| Living<br>2) |__| Deceased<br>3) |__| Does not know	|__|__|__| Years	1) |__| Yes<br>2) |__| No ***(Go to next row)***<br>3) |__| Does not know ***(Go to next row)***	|__|__|__| Cod. Ca1<br>|__|__|__| Cod. Ca2<br>|__|__|__| Cod. Ca3	|__|__|__| Years Ca1<br>|__|__|__| Years Ca2<br>|__|__|__| Years Ca3
1) |__| Maternal<br>2) |__| Paternal	1) |__| Female<br>2) |__| Male	1) |__| Living<br>2) |__| Deceased<br>3) |__| Does not know	|__|__|__| Years	1) |__| Yes<br>2) |__| No ***(Go to next row)***<br>3) |__| Does not know ***(Go to next row)***	|__|__|__| Cod. Ca1<br>|__|__|__| Cod. Ca2<br>|__|__|__| Cod. Ca3	|__|__|__| Years Ca1<br>|__|__|__| Years Ca2<br>|__|__|__| Years Ca3
1) |__| Maternal<br>2) |__| Paternal	1) |__| Female<br>2) |__| Male	1) |__| Living<br>2) |__| Deceased<br>3) |__| Does not know	|__|__|__| Years	1) |__| Yes<br>2) |__| No ***(Go to next row)***<br>3) |__| Does not know ***(Go to next row)***	|__|__|__| Cod. Ca1<br>|__|__|__| Cod. Ca2<br>|__|__|__| Cod. Ca3	|__|__|__| Years Ca1<br>|__|__|__| Years Ca2<br>|__|__|__| Years Ca3
1) |__| Maternal<br>2) |__| Paternal	1) |__| Female<br>2) |__| Male	1) |__| Living<br>2) |__| Deceased<br>3) |__| Does not know	|__|__|__| Years	1) |__| Yes<br>2) |__| No<br>3) |__| Does not know	|__|__|__| Cod. Ca1<br>|__|__|__| Cod. Ca2<br>|__|__|__| Cod. Ca3	|__|__|__| Years Ca1<br>|__|__|__| Years Ca2<br>|__|__|__| Years Ca3

Cancer Codes: Please refer to the cancer code lookup list.

The following questions are about your ***biological*** children.

71. How many children do/did you have?

    Interviewer: If no children, record 00 for daughters and sons; go to 78.
    If unknown for either/both sons or daughters, record 99; continue with questions for those children who are known to the participant.

    |__|__| Daughters
    |__|__| Sons

Starting with your *oldest* child, please answer the following questions:

72. Gender?	73. Living?	74. How old is he/she now or how old was he/she when he/she died? ***(Do not know = 999)***	75. Does/did he/she have cancer?	76. What type(s) of cancer does/did he/she have? ***Interviewer: In case he/she has/had more than one type of cancer, please list all.***	77. At what age was the cancer diagnosed? ***(Do not know = 999)***
1) |__| Female<br>2) |__| Male	1) |__| Living<br>2) |__|Deceased<br>9) |__| Does not know	|__|__|__| Years	1) |__| Yes<br>2) |__| No ***(Go to next row)***<br>9) |__| Does not know ***(Go to next row)***	|__|__|__| Cod. Ca1<br>|__|__|__| Cod. Ca2<br>|__|__|__| Cod. Ca3	|__|__|__| Years Ca1<br>|__|__|__| Years Ca2<br>|__|__|__| Years Ca3
1) |__| Female<br>2) |__| Male	1) |__| Living<br>2) |__| Deceased<br>9) |__| Does not know	|__|__|__| Years	1) |__| Yes<br>2) |__| No***(Go to next row)***<br>9) |__| Does not know ***(Go to next row)***	|__|__|__| Cod. Ca1<br>|__|__|__| Cod. Ca2<br>|__|__|__| Cod. Ca3	|__|__|__| Years Ca1<br>|__|__|__| Years Ca2<br>|__|__|__| Years Ca3
1) |__| Female<br>2) |__| Male	1) |__| Living<br>2) |__|Deceased<br>9) |__| Does not know	|__|__|__| Years	1) |__| Yes<br>2) |__| No ***(Go to next row)***<br>9) |__| Does not know ***(Go to next row)***	|__|__|__| Cod. Ca1<br>|__|__|__| Cod. Ca2<br>|__|__|__| Cod. Ca3	|__|__|__| Years Ca1<br>|__|__|__| Years Ca2<br>|__|__|__| Years Ca3
1) |__| Female<br>2) |__| Male	1) |__| Living<br>2) |__|Deceased<br>9) |__| Does not know	|__|__|__| Years	1) |__| Yes<br>2) |__| No ***(Go to next row)***<br>9) |__| Does not know ***(Go to next row)***	|__|__|__| Cod. Ca1<br>|__|__|__| Cod. Ca2<br>|__|__|__| Cod. Ca3	|__|__|__| Years Ca1<br>|__|__|__| Years Ca2<br>|__|__|__| Years Ca3
1) |__| Female<br>2) |__| Male	1) |__| Living<br>2) |__|Deceased<br>9) |__| Does not know	|__|__|__| Years	1) |__| Yes<br>2) |__| No ***(Go to next row)***<br>9) |__| Does not know ***(Go to next row)***	|__|__|__| Cod. Ca1<br>|__|__|__| Cod. Ca2<br>|__|__|__| Cod. Ca3	|__|__|__| Years Ca1<br>|__|__|__| Years Ca2<br>|__|__|__| Years Ca3
1) |__| Female<br>2) |__| Male	1) |__| Living<br>2) |__|Deceased<br>9) |__| Does not know	|__|__|__| Years	1) |__| Yes<br>2) |__| No ***(Go to next row)***<br>9) |__| Does not know ***(Go to next row)***	|__|__|__| Cod. Ca1<br>|__|__|__| Cod. Ca2<br>|__|__|__| Cod. Ca3	|__|__|__| Years Ca1<br>|__|__|__| Years Ca2<br>|__|__|__| Years Ca3
1) |__| Female<br>2) |__| Male	1) |__| Living<br>2) |__|Deceased<br>9) |__| Does not know	|__|__|__| Years	1) |__| Yes<br>2) |__| No<br>9) |__| Does not know	|__|__|__| Cod. Ca1<br>|__|__|__| Cod. Ca2<br>|__|__|__| Cod. Ca3	|__|__|__| Years Ca1<br>|__|__|__| Years Ca2<br>|__|__|__| Years Ca3

Cancer Codes: Please refer to the cancer code lookup list.

Now, I would like to know about YOUR aunts and uncles. I will start with your maternal aunts and uncles (your mother's sisters and brothers), and then I will ask you about your paternal aunts and uncles (your father's sisters and brothers).

EP05: CANCER HISTORY MODULE OF PARTICIPANT AND FAMILY
(AUNTS' AND UNCLES' FAMILY HISTORY)

The following questions are about your *maternal aunts and uncles*:

78. How many aunts and uncles *on your* ***mother's*** side do/did you have?
Interviewer: If no aunts or uncles on mother's side record 00; go to 85.
If unknown, record 99; continue with questions for those aunts and uncles who are known to the participant.
|__|__| Maternal aunts
|__|__| Maternal uncles

Starting with your *oldest* maternal aunt/uncle please answer the following questions:

79. Gender?	80. Living?	81. How old is he/she now or how old was he/she when he/she died? ***(Do not know = 999)***	82. Does/did he/she have cancer?	83. What type(s) of cancer does/did he/she have? ***Interviewer: In case he/she has/had more than one type of cancer, please list all.***	84. At what age was the cancer diagnosed? ***(Do not know = 999)***
1) |__| Female<br>2) |__| Male	1) |__| Living<br>2) |__|Deceased<br>9) |__| Does not know	|__|__|__| Years	1) |__| Yes<br>2) |__| No ***(Go to next row)***<br>9) |__| Does not know ***(Go to next row)***	|__|__|__| Cod. Ca1<br>|__|__|__| Cod. Ca2<br>|__|__|__| Cod. Ca3	|__|__|__| Years Ca1<br>|__|__|__| Years Ca2<br>|__|__|__| Years Ca3
1) |__| Female<br>2) |__| Male	1) |__| Living<br>2) |__| Deceased<br>9) |__| Does not know	|__|__|__| Years	1) |__| Yes<br>2) |__| No***(Go to next row)***<br>9) |__| Does not know ***(Go to next row)***	|__|__|__| Cod. Ca1<br>|__|__|__| Cod. Ca2<br>|__|__|__| Cod. Ca3	|__|__|__| Years Ca1<br>|__|__|__| Years Ca2<br>|__|__|__| Years Ca3
1) |__| Female<br>2) |__| Male	1) |__| Living<br>2) |__|Deceased<br>9) |__| Does not know	|__|__|__| Years	1) |__| Yes<br>2) |__| No ***(Go to next row)***<br>9) |__| Does not know ***(Go to next row)***	|__|__|__| Cod. Ca1<br>|__|__|__| Cod. Ca2<br>|__|__|__| Cod. Ca3	|__|__|__| Years Ca1<br>|__|__|__| Years Ca2<br>|__|__|__| Years Ca3
1) |__| Female<br>2) |__| Male	1) |__| Living<br>2) |__|Deceased<br>9) |__| Does not know	|__|__|__| Years	1) |__| Yes<br>2) |__| No ***(Go to next row)***<br>9) |__| Does not know ***(Go to next row)***	|__|__|__| Cod. Ca1<br>|__|__|__| Cod. Ca2<br>|__|__|__| Cod. Ca3	|__|__|__| Years Ca1<br>|__|__|__| Years Ca2<br>|__|__|__| Years Ca3
1) |__| Female<br>2) |__| Male	1) |__| Living<br>2) |__|Deceased<br>9) |__| Does not know	|__|__|__| Years	1) |__| Yes<br>2) |__| No ***(Go to next row)***<br>9) |__| Does not know ***(Go to next row)***	|__|__|__| Cod. Ca1<br>|__|__|__| Cod. Ca2<br>|__|__|__| Cod. Ca3	|__|__|__| Years Ca1<br>|__|__|__| Years Ca2<br>|__|__|__| Years Ca3
1) |__| Female<br>2) |__| Male	1) |__| Living<br>2) |__|Deceased<br>9) |__| Does not know	|__|__|__| Years	1) |__| Yes<br>2) |__| No ***(Go to next row)***<br>9) |__| Does not know ***(Go to next row)***	|__|__|__| Cod. Ca1<br>|__|__|__| Cod. Ca2<br>|__|__|__| Cod. Ca3	|__|__|__| Years Ca1<br>|__|__|__| Years Ca2<br>|__|__|__| Years Ca3
1) |__| Female<br>2) |__| Male	1) |__| Living<br>2) |__|Deceased<br>9) |__| Does not know	|__|__|__| Years	1) |__| Yes<br>2) |__| No<br>9) |__| Does not know	|__|__|__| Cod. Ca1<br>|__|__|__| Cod. Ca2<br>|__|__|__| Cod. Ca3	|__|__|__| Years Ca1<br>|__|__|__| Years Ca2<br>|__|__|__| Years Ca3

79. Gender?	80. Living?	81. How old is he/she now or how old was he/she when he/she died? ***(Do not know = 999)***	82. Does/did he/she have cancer?	83. What type(s) of cancer does/did he/she have? ***Interviewer: In case he/she has/had more than one type of cancer, please list all.***	84. At what age was the cancer diagnosed? ***(Do not know = 999)***
1) |__| Female<br>2) |__| Male	1) |__| Living<br>2) |__|Deceased<br>9) |__| Does not know	|__|__|__| Years	1) |__| Yes<br>2) |__| No ***(Go to next row)***<br>9) |__| Does not know ***(Go to next row)***	|__|__|__| Cod. Ca1<br>|__|__|__| Cod. Ca2<br>|__|__|__| Cod. Ca3	|__|__|__| Years Ca1<br>|__|__|__| Years Ca2<br>|__|__|__| Years Ca3
1) |__| Female<br>2) |__| Male	1) |__| Living<br>2) |__|Deceased<br>9) |__| Does not know	|__|__|__| Years	1) |__| Yes<br>2) |__| No***(Go to next row)***<br>9) |__| Does not know ***(Go to next row)***	|__|__|__| Cod. Ca1<br>|__|__|__| Cod. Ca2<br>|__|__|__| Cod. Ca3	|__|__|__| Years Ca1<br>|__|__|__| Years Ca2<br>|__|__|__| Years Ca3
1) |__| Female<br>2) |__| Male	1) |__| Living<br>2) |__|Deceased<br>9) |__| Does not know	|__|__|__| Years	1) |__| Yes<br>2) |__| No ***(Go to next row)***<br>9) |__| Does not know ***(Go to next row)***	|__|__|__| Cod. Ca1<br>|__|__|__| Cod. Ca2<br>|__|__|__| Cod. Ca3	|__|__|__| Years Ca1<br>|__|__|__| Years Ca2<br>|__|__|__| Years Ca3
1) |__| Female<br>2) |__| Male	1) |__| Living<br>2) |__|Deceased<br>9) |__| Does not know	|__|__|__| Years	1) |__| Yes<br>2) |__| No ***(Go to next row)***<br>9) |__| Does not know ***(Go to next row)***	|__|__|__| Cod. Ca1<br>|__|__|__| Cod. Ca2<br>|__|__|__| Cod. Ca3	|__|__|__| Years Ca1<br>|__|__|__| Years Ca2<br>|__|__|__| Years Ca3
1) |__| Female<br>2) |__| Male	1) |__| Living<br>2) |__|Deceased<br>9) |__| Does not know	|__|__|__| Years	1) |__| Yes<br>2) |__| No ***(Go to next row)***<br>9) |__| Does not know ***(Go to next row)***	|__|__|__| Cod. Ca1<br>|__|__|__| Cod. Ca2<br>|__|__|__| Cod. Ca3	|__|__|__| Years Ca1<br>|__|__|__| Years Ca2<br>|__|__|__| Years Ca3
1) |__| Female<br>2) |__| Male	1) |__| Living<br>2) |__|Deceased<br>9) |__| Does not know	|__|__|__| Years	1) |__| Yes<br>2) |__| No ***(Go to next row)***<br>9) |__| Does not know ***(Go to next row)***	|__|__|__| Cod. Ca1<br>|__|__|__| Cod. Ca2<br>|__|__|__| Cod. Ca3	|__|__|__| Years Ca1<br>|__|__|__| Years Ca2<br>|__|__|__| Years Ca3
1) |__| Female<br>2) |__| Male	1) |__| Living<br>2) |__|Deceased<br>9) |__| Does not know	|__|__|__| Years	1) |__| Yes<br>2) |__| No<br>9) |__| Does not know	|__|__|__| Cod. Ca1<br>|__|__|__| Cod. Ca2<br>|__|__|__| Cod. Ca3	|__|__|__| Years Ca1<br>|__|__|__| Years Ca2<br>|__|__|__| Years Ca3

Cancer Codes: Please refer to the cancer code lookup list.

The following questions are about your ***paternal*** aunts and uncles:

85. How many aunts and uncles on your ***father's*** side do/did you have?
Interviewer: If no aunts or uncles on father's side record 00; go to 92.
If unknown, record 99 ; continue with questions for those aunts and uncles who are known to the participant.
|__|__| Paternal aunts
|__|__| Paternal uncles

Starting with your *oldest* paternal aunt/uncle please answer the following questions:

<table>
<tr><th>86. Gender?</th><th>87. Living?</th><th>88. How old is he/she now or how old was he/she when he/she died?
(Do not know = 999)</th><th>89. Does/did he/she have cancer?</th><th>90. What type(s) of cancer does/did he/she have?
Interviewer: In case he/she has/had more than one type of cancer, please list all.</th><th>91. At what age was the cancer diagnosed?
(Do not know = 999)</th></tr>
<tr><td>1) |__| Female
2) |__| Male</td><td>1) |__| Living
2) |__|Deceased
9) |__| Does not know</td><td>|__|__|__| Years</td><td>1) |__| Yes
2) |__| No ***(Go to next row)***
9) |__| Does not know ***(Go to next row)***</td><td>|__|__|__| Cod. Ca1
|__|__|__| Cod. Ca2
|__|__|__| Cod. Ca3</td><td>|__|__|__| Years Ca1
|__|__|__| Years Ca2
|__|__|__| Years Ca3</td></tr>
<tr><td>1) |__| Female
2) |__| Male</td><td>1) |__| Living
2) |__| Deceased
9) |__| Does not know</td><td>|__|__|__| Years</td><td>1) |__| Yes
2) |__| No***(Go to next row)***
9) |__| Does not know ***(Go to next row)***</td><td>|__|__|__| Cod. Ca1
|__|__|__| Cod. Ca2
|__|__|__| Cod. Ca3</td><td>|__|__|__| Years Ca1
|__|__|__| Years Ca2
|__|__|__| Years Ca3</td></tr>
<tr><td>1) |__| Female
2) |__| Male</td><td>1) |__| Living
2) |__|Deceased
9) |__| Does not know</td><td>|__|__|__| Years</td><td>1) |__| Yes
2) |__| No ***(Go to next row)***
9) |__| Does not know ***(Go to next row)***</td><td>|__|__|__| Cod. Ca1
|__|__|__| Cod. Ca2
|__|__|__| Cod. Ca3</td><td>|__|__|__| Years Ca1
|__|__|__| Years Ca2
|__|__|__| Years Ca3</td></tr>
<tr><td>1) |__| Female
2) |__| Male</td><td>1) |__| Living
2) |__|Deceased
9) |__| Does not know</td><td>|__|__|__| Years</td><td>1) |__| Yes
2) |__| No ***(Go to next row)***
9) |__| Does not know ***(Go to next row)***</td><td>|__|__|__| Cod. Ca1
|__|__|__| Cod. Ca2
|__|__|__| Cod. Ca3</td><td>|__|__|__| Years Ca1
|__|__|__| Years Ca2
|__|__|__| Years Ca3</td></tr>
<tr><td>1) |__| Female
2) |__| Male</td><td>1) |__| Living
2) |__|Deceased
9) |__| Does not know</td><td>|__|__|__| Years</td><td>1) |__| Yes
2) |__| No ***(Go to next row)***
9) |__| Does not know ***(Go to next row)***</td><td>|__|__|__| Cod. Ca1
|__|__|__| Cod. Ca2
|__|__|__| Cod. Ca3</td><td>|__|__|__| Years Ca1
|__|__|__| Years Ca2
|__|__|__| Years Ca3</td></tr>
<tr><td>1) |__| Female
2) |__| Male</td><td>1) |__| Living
2) |__|Deceased
9) |__| Does not know</td><td>|__|__|__| Years</td><td>1) |__| Yes
2) |__| No ***(Go to next row)***
9) |__| Does not know ***(Go to next row)***</td><td>|__|__|__| Cod. Ca1
|__|__|__| Cod. Ca2
|__|__|__| Cod. Ca3</td><td>|__|__|__| Years Ca1
|__|__|__| Years Ca2
|__|__|__| Years Ca3</td></tr>
<tr><td>1) |__| Female
2) |__| Male</td><td>1) |__| Living
2) |__|Deceased
9) |__| Does not know</td><td>|__|__|__| Years</td><td>1) |__| Yes
2) |__| No
9) |__| Does not know</td><td>|__|__|__| Cod. Ca1
|__|__|__| Cod. Ca2
|__|__|__| Cod. Ca3</td><td>|__|__|__| Years Ca1
|__|__|__| Years Ca2
|__|__|__| Years Ca3</td></tr>
</table>

Cancer Codes: Please refer to the cancer code lookup list.

92. Evaluation of Cancer History of Participant and Familiy Module
    1) |__| Unsatisfactory
    2) |__| Questionable
    3) |__| Reliable

EP06: HORMONAL AND REPRODUCTIVE HISTORY MODULE

The following questions are about your menstrual periods, pregnancies, menopause, and hormones that you take or have taken.

1. How old were you when you had your first menstrual period?
   |__|__|__| Years ***(Does not know = 999)***

2. Considering all your pregnancies (live births or stillbirths, miscarriages, tubal pregnancy), how many times have you been pregnant?
   |__|__| Times ***(Does not know = 99)***

Now, I would like to learn details about each one of your pregnancies that either resulted in a live birth or lasted 6 months or more.

The number of pregnancies in the table below will depend on the number of times you have given birth or delivered a stillborn (i.e., carried a baby for 6 months or more).

Interviewer: Ask questions 3 to 7 for each live birth (or any pregnancy that lasted six months or more) in chronological order, starting with the first birth/stillbirth up to the most recent one. Ask all the questions about one birth/stillbirth before going on to the next one.

<table>
<tr><th></th><th>1st Birth</th><th>2nd Birth</th><th>3rd Birth</th><th>4th Birth</th><th>5th Birth</th><th>6th Birth</th><th>7th Birth</th></tr>
<tr><td>3. How old were you at the time of your first live birth (or age at stillbirth [pregnancy lasting 6 months or more])? How old were you at the time of each of your subsequent births?</td><td>|__|__|__|
Years
(Does not know = 999)</td><td>|__|__|__|
Years
(Does not know = 999)</td><td>|__|__|__|
Years
(Does not know = 999)</td><td>|__|__|__|
Years
(Does not know = 999)</td><td>|__|__|__|
Years
(Does not know = 999)</td><td>|__|__|__|
Years
(Does not know = 999)</td><td>|__|__|__|
Years
(Does not know = 999)</td></tr>
<tr><td>4. How many babies were born? Record information on live and dead only.</td><td>|__|__|
Live
|__|__|
Dead
|__|__|
Abortions
|__|__|
Miscarriages</td><td>|__|__|
Live
|__|__|
Dead
|__|__|
Abortions
|__|__|
Miscarriages</td><td>|__|__|
Live
|__|__|
Dead
|__|__|
Abortions
|__|__|
Miscarriages</td><td>|__|__|
Live
|__|__|
Dead
|__|__|
Abortions
|__|__|
Miscarriages</td><td>|__|__|
Live
|__|__|
Dead
|__|__|
Abortions
|__|__|
Miscarriages</td><td>|__|__|
Live
|__|__|
Dead
|__|__|
Abortions
|__|__|
Miscarriages</td><td>|__|__|
Live
|__|__|
Dead
|__|__|
Abortions
|__|__|
Miscarriages</td></tr>
<tr><td>5. How long did this pregnancy last?</td><td>|__|__|
Weeks
(Does not know = 99) If in question 4, live=0 AND dead ≥1, go to next birth or go to 8.</td><td>|__|__|
Weeks
(Does not know = 99) If in question 4, live=0 AND dead ≥1, go to next birth or go to 8.</td><td>|__|__|
Weeks
(Does not know = 99) If in question 4, live=0 AND dead ≥1, go to next birth or go to 8.</td><td>|__|__|
Weeks
(Does not know = 99) If in question 4, live=0 AND dead ≥1, go to next birth or go to 8.</td><td>|__|__|
Weeks
(Does not know = 99) If in question 4, live=0 AND dead ≥1, go to next birth or go to 8.</td><td>|__|__|
Weeks
(Does not know = 99) If in question 4, live=0 AND dead ≥1, go to next birth or go to 8.</td><td>|__|__|
Weeks
(Does not know = 99) If in question 4, live=0 AND dead ≥1, go to next birth or go to 8.</td></tr>
</table>

6. Did you breast-feed this baby (or these babies), even if for a short time?	1) |__| Yes<br>2) |__| No ***(Go to next birth or go to 8)***<br>9) |__| Does not know ***(Go to next birth or go to 8).***	1) |__| Yes<br>2) |__| No ***(Go to next birth or go to 8)***<br>9) |__| Does not know ***(Go to next birth or go to 8).***	1) |__| Yes<br>2) |__| No ***(Go to next birth or go to 8)***<br>9) |__| Does not know ***(Go to next birth or go to 8).***	1) |__| Yes<br>2) |__| No ***(Go to next birth or go to 8)***<br>9) |__| Does not know ***(Go to next birth or go to 8).***	1) |__| Yes<br>2) |__| No ***(Go to next birth or go to 8)***<br>9) |__| Does not know ***(Go to next birth or go to 8).***	1) |__| Yes<br>2) |__| No ***(Go to next birth or go to 8)***<br>9) |__| Does not know ***(Go to next birth or go to 8).***	1) |__| Yes<br>2) |__| No ***(Go to next birth or go to 8)***<br>9) |__| Does not know ***(Go to next birth or go to 8).***
7. For how long did you breast-feed this baby (or these babies)?	|__|__| Years<br>|__|__| Months<br>***<1 month = 00 Does not know = 99. (Go to next birth or go to 8).***	|__|__| Years<br>|__|__| Months<br>***<1 month = 00 Does not know = 99. (Go to next birth or go to 8).***	|__|__| Years<br>|__|__| Months<br>***<1 month = 00 Does not know = 99. (Go to next birth or go to 8).***	|__|__| Years<br>|__|__| Months<br>***<1 month = 00 Does not know = 99. (Go to next birth or go to 8).***	|__|__| Years<br>|__|__| Months<br>***<1 month = 00 Does not know = 99. (Go to next birth or go to 8).***	|__|__| Years<br>|__|__| Months<br>***<1 month = 00 Does not know = 99. (Go to next birth or go to 8).***	|__|__| Years<br>|__|__| Months<br>***<1 month = 00 Does not know = 99. (Go to next birth or go to 8).***

8. Have you ever tried to get pregnant for a year or more and not become pregnant?
   1) |__| Yes
   2) |__| No
   9) |__| Does not know

9. Have you ever undergone treatments to become pregnant or maintain a pregnancy?
   1) |__| Yes
   2) |__| No ***(Go to 11)***
   9) |__| Does not know ***(Go to 11)***

10. What treatment did you do for this?
Interviewer: Read each item and mark Yes, No, or Do not know.

	Yes	No	Does not know
In vitro fertilization?	1.|__|	2.|__|	9.|__|
Artificial insemination?	1.|__|	2.|__|	9.|__|
Hormone treatment? (Specify): ______________________	1.|__|	2.|__|	9.|__|
Any other treatment? (Specify): ___________________________	1.|__|	2.|__|	9.|__|

The following questions are about some methods to avoid pregnancy and hormones that you would have used or are using.

Interviewer: The next questions are about the hormonal birth control methods that the participant could have used. We are not interested in the methods such as condoms, diaphragm, sperm gel, etc.

11. Did you take BIRTH CONTROL PILLS to avoid pregnancy or for any other reason, such as irregular menstrual cycle, acne, colics, endometriosis or polycystic ovary?
   1) |__| Yes, I used them in the past (but no longer using). ***(Go to 12)***
   2) |__| Yes, I am currently using them. ***(Go to 13)***
   3) |__| I never used them. ***(Go to 14)***

12. How long ago did you stop using birth control pills?
   Interviewer: Read all choices.
   1) |__| Less than 1 year ago
   2) |__| From 1 to 4 years ago
   3) |__| From 5 to 9 years ago
   4) |__| 10 years ago or more
   9) |__| Does not know

13. Adding up all the periods of time you used birth control pills, for any reason, how long did you use them in total? Exclude periods when you were not using them.
    Interviewer: Help the participant add up all the periods of time when she was using birth control pills and excluding those when she did not use them.
    Interviewer: Does not know Years = 999, does not know Months = 99; < 1 Month = 00.
    |__|__|__| Years and |__|__| Months

Now, I will read a list of other methods to avoid pregnancy that you may have used. Please answer if you have used them, are using them now, or never have used them. For each method you list, please indicate the length of time you used it.

14. Have you used, are you now using or did you never use ....? ***Interviewer: Ask about each method. For methods the participant used or is now using, ask question 15. Otherwise, go to 16.***		15. For how long did you use/have you been using...? Do not include periods of time when you did not use.
a. Hormone injection	1) |__| I used them ***(go to 15)***<br>2) |__| I am now using ***(go to 15)***<br>3) |__| I never used<br>9) |__| Does not know	|__|__|__| and |__|__| Years Months<br>***<1 month = 00 and 00***<br>***Does not know = 999 and 00***
b. Hormone implant (including adhesive/patches)	1) |__| I used them ***(go to 15)***<br>2) |__| I am now using ***(go to 15)***<br>3) |__| I never used<br>9) |__| Does not know	|__|__|__| and |__|__| Years Months<br>***<1 month = 00 and 00***<br>***Does not know = 999 and 00***
c.Vaginal ring (Nuvaring)	1) |__| I used them ***(go to 15)***<br>2) |__| I am now using ***(go to 15)***<br>3) |__| I never used<br>9) |__| Does not know	|__|__|__| and |__|__| Years Months<br>***<1 month = 00 and 00***<br>***Does not know = 999 and 00***
d. Hormonal IUD (Mirena)	1) |__| I used them ***(go to 15)***<br>2) |__| I am now using ***(go to 15)***<br>3) |__| I never used<br>9) |__| Does not know	|__|__|__| and |__|__| Years Months<br>***<1 month = 00 and 00***<br>***Does not know = 999 and 00***

16. Are you still menstruating?
    1) |__| Yes ***(Go to 19)***
    2) |__| No
    9) |__| Does not know

The next questions are about Menopause. Menopause is the permanent end of menstruation and fertility, defined as occurring 12 months after your last menstrual period.

To treat menopause symptoms, sometimes doctors prescribe drugs that are hormones. These drugs, termed replacement hormones, can be used as pills, injections, adhesive patches, gel to place on body, or as vaginal creams.

17. At what age did you complete menopause; i.e., stop having your menstrual periods (age at menopause)?
    |__|__|__| Years ***(Does not know = 999)***

18. Why do you no longer menstruate?
    Interviewer: Read each item and mark only one choice.
    1) |__| Menstruation stopped naturally.
    2) |__| Menstruation stopped due to surgery for removal of uterus or ovaries.
    3) |__| Menstruation stopped due to radiation therapy or chemotherapy.
    4) |__| Other (Specific): ____
    9) |__| Does not know

19. Have you at any time used hormone treatment for menopause symptoms (hormone replacement)?
    1) |__| Yes
    2) |__| No ***(Go to 23)***
    9) |__| Does not know ***(Go to 23)***

20. What did you use?
    Interviewer: Read each item and mark Yes, No, or Does not know.

	Yes	No	Does not know
Pill	1.|__|	2.|__|	9.|__|
Patch or adhesive	1.|__|	2.|__|	9.|__|
Gel	1.|__|	2.|__|	9.|__|
Cream	1.|__|	2.|__|	9.|__|
Other? (Specify): ________________________________ _______	1.|__|	2.|__|	9.|__|

Interviewer: If answer to 20 is 'Yes' for any of the Hormone Replacement options, go to 21. Otherwise go to 23.

21. Do you know if the medication you took was only estrogen, only progesterone, or a combination of the two or did you take two types of pills?
    1) |__| Estrogen only
    2) |__| Progesterone only
    3) |__| Combined estrogen and progesterone
    4) |__| Two medications: one estrogen and the other progesterone
    9) |__| Does not know

22. Adding up all the time you used these medications, what was the total time you used them? Exclude the periods of time that you normally did not use them.
    Interviewer: Help the participant to add up all the periods of time that she used replacement hormones and exclude those during which she did not use them.
    |__|__|__|Years and |__|__| Months
    (Does not know Years = 999; does not know Months = 99; < 1 Month = 00)

23. Evaluation of Hormonal and Reproductive History Module:
    1) |__| Unsatisfactory
    2) |__| Questionable
    3) |__| Reliable

EP07: MEDICAL HISTORY MODULE

The following questions are about certain health diseases or disorders that you have or may have had in the past.
Interviewer: Ask about each disorder. If the participant answers 'Yes' to any disorder, go to 2. Otherwise, go to the next disorder.

Has any doctor told you that you have or have (ever) had any of the following health disorders: ?
Otherwise, go to the next disorder.

		1. What year were you diagnosed?	2. As a result of this disorder, were you hospitalized, did you have surgery, or was some medicine suggested?
a. Arterial Hypertension/ High Blood Pressure	1) |__| Yes<br>2) |__| No ***(Go to b)***<br>9) |__| Does not know ***(Go to b)***	|__|__|__| Age ***(Does not know = 999)***<br>**or**<br>|__|__|__|__| Year diagnosed ***(Does not know = 9999)***	1. |__| Hospitalized<br>2. |__| Surgery<br>3. |__| Medication<br>4. |__| No treatment
b. Diabetes Mellitus/ Blood Sugar	1) |__| Yes<br>2) |__| No ***(Go to c)***<br>9) |__| Does not know ***(Go to c)***	|__|__|__| Age ***(Does not know = 999)***<br>**or**<br>|__|__|__|__| Year diagnosed ***(Does not know = 9999)***	1. |__| Hospitalized<br>2. |__| Surgery<br>3. |__| Medication<br>4. |__| No treatment
c. Hypercholesterol-emia/ High Cholesterol	1) |__| Yes<br>2) |__| No ***(Go to d)***<br>9) |__| Does not know ***(Go to d)***	|__|__|__| Age ***(Does not know = 999)***<br>**or**<br>|__|__|__|__| Year diagnosed ***(Does not know = 9999)***	1. |__| Hospitalized<br>2. |__| Surgery<br>3. |__| Medication<br>4. |__| No treatment
d. Asthma	1) |__| Yes<br>2) |__| No ***(Go to e)***<br>9) |__| Does not know ***(Go to e)***	|__|__|__| Age ***(Does not know = 999)***<br>**or**<br>|__|__|__|__| Year diagnosed ***(Does not know = 9999)***	1. |__| Hospitalized<br>2. |__| Surgery<br>3. |__| Medication<br>4. |__| No treatment
e. COPD/ Emphysema	1) |__| Yes<br>2) |__| No ***(Go to f)***<br>9) |__| Does not know ***(Go to f)***	|__|__|__| Age ***(Does not know = 999)***<br>**or**<br>|__|__|__|__| Year diagnosed ***(Does not know = 9999)***	1. |__| Hospitalized<br>2. |__| Surgery<br>3. |__| Medication<br>4. |__| No treatment
f. Epilepsy	1) |__| Yes<br>2) |__| No ***(Go to g)***<br>9) |__| Does not know ***(Go to g)***	|__|__|__| Age ***(Does not know = 999)***<br>**or**<br>|__|__|__|__| Year diagnosed ***(Does not know = 9999)***	1. |__| Hospitalized<br>2. |__| Surgery<br>3. |__| Medication<br>4. |__| No treatment
g. Cardiac Failure/ Large Heart	1) |__| Yes<br>2) |__| No ***(Go to h)***<br>9) |__| Does not know ***(Go to h)***	|__|__|__| Age ***(Does not know = 999)***<br>**or**<br>|__|__|__|__| Year diagnosed ***(Does not know = 9999)***	1. |__| Hospitalized<br>2. |__| Surgery<br>3. |__| Medication<br>4. |__| No treatment

Continued		1. What year were you diagnosed?	2. As a result of this disorder, were you hospitalized, did you have surgery, or was some medicine suggested?
h. Stroke	1) |__| Yes<br>2) |__| No ***(Go to i)***<br>9) |__| Does not know ***(Go to i)***	|__|__|__|Age ***(Does not know = 999)***<br>**or**<br>|__|__|__|__| Year diagnosed ***(Does not know = 9999)***	1. |__| Hospitalized<br>2. |__| Surgery<br>3. |__| Medication<br>4. |__| No treatment
i. Liver Cirrhosis	1) |__| Yes<br>2) |__| No ***(Go to j)***<br>9) |__| Does not know ***(Go to j)***	|__|__|__| Age ***(Does not know = 999)***<br>**or**<br>|__|__|__|__| Year diagnosed ***(Does not know = 9999)***	1. |__| Hospitalized<br>2. |__| Surgery<br>3. |__| Medication<br>4. |__| No treatment
j. Chronic Hepatitis	1) |__| Yes<br>2) |__| No ***(Go to k)***<br>9) |__| Does not know ***(Go to k)***	|__|__|__| Age ***(Does not know = 999)***<br>**or**<br>|__|__|__|__| Year diagnosed ***(Does not know = 9999)***	1. |__| Hospitalized<br>2. |__| Surgery<br>3. |__| Medication<br>4. |__| No treatment
k. Kidney Failure/ other kidney problems	1) |__| Yes<br>2) |__| No ***(Go to l)***<br>9) |__| Does not know ***(Go to l)***	|__|__|__|Age ***(Does not know = 999)***<br>**or**<br>|__|__|__|__| Year diagnosed ***(Does not know = 9999)***	1. |__| Hospitalized<br>2. |__| Surgery<br>3. |__| Medication<br>4. |__| No treatment
l. Chagas Disease	1) |__| Yes<br>2) |__| No ***(Go to m)***<br>9) |__| Does not know ***(Go to m)***	|__|__|__| Age ***(Does not know = 999)***<br>**or**<br>|__|__|__|__| Year diagnosed ***(Does not know = 9999)***	1. |__| Hospitalized<br>2. |__| Surgery<br>3. |__| Medication<br>4. |__| No treatment
m. Are there any other diseases that you would like to mention? If so, please specify.	1) |__| Yes<br>If yes, enter disease(s):<br>____________________<br>2) |__| No<br>9) |__| Does not know	|__|__|__|Age ***(Does not know = 999)***<br>**or**<br>|__|__|__|__| Year diagnosed ***(Does not know = 9999)***	1. |__| Hospitalized<br>2. |__| Surgery<br>3. |__| Medication<br>4. |__| No treatment

3. Evaluation of Medical History Module:
    1) |__| Unsatisfactory
    2) |__| Questionable
    3) |__| Reliable

EP08: MODULE OF HABITS - TOBACCO SMOKING

The following questions are about the use of tobacco products. I will start by asking about the use of industrialized (store-bought) cigarettes.
Interviewer: Do not consider cigars, homemade cigarettes, 'cigarritos' (small home-made** cigarettes **made from rolled tobacco leaves), or marijuana.

1. Are you a:
   1) |__| current daily or occasional smoker
   2) |__| ex-smoker ***(Go to 6)***
   3) |__| never smoked ***(Go to 18)***

Currently Daily and Occasional Smoker

2. How old were you when you started smoking cigarettes?
   |__|__|__|Years ***(Does not know = 999)***

3. On an average, how many cigarettes do you smoke?
   Interviewer: Record the amount and mark the frequency (day, week, or month). If the response is in packages or cartons, ask how many in each, and calculate the total number of cigarettes.

   |__|__|__| Cigarettes per
   (Does not know = 999)

   1) |__| Day
   2) |__| Week
   3) |__| Month
   9) |__| Does not know

4. Did you ***(ever)*** stop smoking cigarettes for a period of 1 year or more?
   1) |__| Yes
   2) |__| No ***(Go to 12)***

5. Combining all the periods when you stopped smoking cigarettes, how long did you stop smoking?
   |__|__|__| Years ***(Does not know =999)***

Ex-Smoker

6. How old were you when you started smoking cigarettes?
   |__|__|__| Years ***(Does not know = 999)***

7. How old were you when you stopped smoking cigarettes?
   |__|__|__|Years ***Interviewer: Does not know = 999 and go to 8.***

8. How long ago did you stop smoking cigarettes?
   |__|__|__| Years ***(Does not know: Years =999; < 1 Year = 000)***

9. On the average, how many cigarettes did you smoke?
Interviewer: Record the amount and mark the frequency (day, week, or month). Should the response be in packages or cartons, ask how many in each and calculate the total number of cigarettes.

|__|__|__| Cigarettes per
(Does not know = 999)

1) |__| Day
2) |__| Week
3) |__| Month
9) |__| Does not know

10. During the years you smoked cigarettes, did you stop smoking cigarettes for a period of time of 1 year or more?
1) |__| Yes
2) |__| No ***(Go to 12)***

11. During the years you smoked cigarettes, combining all the periods of time you stopped smoking cigarettes, in total, how long did you stop smoking?
|__|__|__|Years ***(Does not know = 999)***

Now I will ask you about homemade cigarettes and 'cigarritos' (i.e., small homemade cigarettes made from rolled tobacco leaves). Please note that we are only asking about tobacco products (NOT marijuana) in these questions.

	Homemade cigarettes or 'cigarritos' (small homemade cigarettes made from rolled tobacco leaves)
12. Do you ***now or have you ever*** smoked homemade cigarettes or cigarritos at least once per week?	1) |__| Yes<br>2) |__| No ***(Go to 18)***
13. How old were you when you started smoking at least one of these per week?	|__|__|__| Years<br>***(Does not know = 999)***
14. How old were you when you last smoked these cigarettes or 'cigarritos'?	|__|__|__| Years<br>***(Does not know = 999) (Still smokes them, code current age)***
15. During the time you smoked them, on average, how many did you normally smoke per week?	|__|__| Cigarettes or 'cigarritos'/week<br>***(Less than 1/Week = 00; Varied a lot = 96; Does not know = 99)***
16. During the time you smoked these homemade cigarettes or 'cigarritos', was there a period of time when you stopped smoking them?	1) |__| Yes<br>2) |__| No ***(Go to 18)***
17. Combining all the periods of time you stopped smoking them, how long did you stop?	|__|__|__| Years ***(Go to 18)***<br>***(Does not know = 999)***

Environmental Exposure to Cigarette Smoke [Please include exposure to both industrial and homemade cigarrettes and/or cigarritos.]

18. At some time during your life, did anyone in your home, including individuals who worked there daily, smoke in your presence?
    1) |__| Yes
    2) |__| No ***(Go to 23)***
    9) |__| Does not remember ***(Go to 23)***

19. Of all the individuals who worked or lived in your home, including those who worked daily in your home, how many individuals smoked in your presence?
    |__|__| Individuals ***(Does not know = 99)***

20. What was your relationship to the person who smoked in your home?	21. How old were you when this individual smoked for the first time in your presence? ***(Does not know Years =999)***	22. How old were you when this individual smoked for the last time in your presence? ***(Does not know Years =999)***
	|__|__|__| Years	|__|__|__| Years
	|__|__|__| Years	|__|__|__| Years
	|__|__|__| Years	|__|__|__| Years
	|__|__|__| Years	|__|__|__| Years
	|__|__|__| Years	|__|__|__| Years
	|__|__|__| Years	|__|__|__| Years

23. Evaluation of Module of Habits - Tobacco Smoking:
    1) |__| Unsatisfactory
    2) |__| Questionable
    3) |__| Reliable

EP09: MODULE OF HABITS: ALCOHOL CONSUMPTION

The following questions are about alcoholic consumption. For purposes of this study, we will consider one can of beer, one glass of wine, and one drink of liquor or whiskey as one alcoholic drink.

1. During your whole life, was there any period of time when you consumed at least one drink of alcoholic beverage such as beer, wine, whiskey, liquor, etc. per month for at least 6 months?
   1) |__| Yes
   2) |__| No ***(Go to 6)***
   9) |__| Does not know ***(Go to 6)***

2. How old were you when you started drinking at least one alcoholic drink per month?
   |__|__|__| Years ***(Does not know = 999)***

3. During the last year (last 12 months), what was the average number of drinks of alcoholic beverages that you consumed?
   1) |__| No drink or less than one per month ***(Go to 4)***
   2) |__| 1 to 3 drinks per month ***(Go to 6)***
   3) |__| 1 drink per week ***(Go to 6)***
   4) |__| 2 to 6 drinks per week ***(Go to 6)***
   5) |__| 7 to 13 drinks per week (***at least*** 1, but less than 2 drinks per day) ***(Go to 6)***
   6) |__| 14 to 20 drinks per week (***at least*** 2, but less than 3 drinks per day) ***(Go to 6)***
   7) |__| 21 to 27 drinks per week (***at least*** 3, but less than 4 drinks per day) ***(Go to 6)***
   8) |__| 28 or more drinks per week (4 or more drinks per day) ***(Go to 6)***
   9) |__| Does not know ***(Go to 6)***

4. How old were you when you stopped consuming at least one alcoholic drink per month?
   Interviewer: Do not consider periods of time when participant consumed alcoholic beverages less than once per month.
   |__|__|__| Years ***(Does not know = 999)***

5. How long ago did you stop consuming at least one alcholic beverage per month?
   |__|__|__| Years ***(Does not know: Years =999; < 1 Year = 000)***

6. Evaluation of Module of Habits: Alcohol Consumption:
   1) |__| Unsatisfactory
   2) |__| Questionable
   3) |__| Reliable

EP10: MODULE OF ANTHROPOMORPHIC FACTORS AND PHYSICAL ACTIVITY

1. How tall are you now?
   |__| , |__|__| Meters ***(Does not know = 999)***

2. What is your current weight?
   |__|__|__| , |__| |__| Kg ***(Does not know = 999)***

3. Not considering the times you were pregnant or nursing, what was your usual weight when you were...
   20 years old? |__|__|__| , |__| Kg
   30 years old? |__|__|__| , |__| Kg
   40 years old? |__|__|__| , |__| Kg
   50 years old? |__|__|__| , |__| Kg
   60 years old? |__|__|__| , |__| Kg
   70 years old? |__|__|__| , |__| Kg
   (Does not know = 999)

4. What is the highest weight you have reached (not considering your weight during pregnancy)?

   |__|__|__| , |__| Kg ***(Does not know = 999)***

5. How old were you when you weighed that much?
   |__|__|__| Years ***(Does not know = 999)***

Physical Activity

6. Other than at work, have you ever in your life done regular physical activities or exercise, that is, at least once per week, ***for 3 months or more***?
   1) |__| Yes ***(Go to 7)***
   2) |__| No ***(Go to 12)***

I would like for you to tell me about these activities and/or exercises, starting with those you did when you were younger.

7. What physical activities or exercises have you performed or do you perform regularly? ***Interviewer: Enter physical activities specified by participant and corresponding codes from the table below.***	8. What year did you start this activity? ***(Does not know = 9999)***	9. What year did you stop this activity? ***(Ongoing activity = Current Year, Does not know = 9999)***	10. How many months per year did you or do you perform this activity? ***(Does not know = 99)***	11. On the average, how many hours per week did you or do you perform this activity? ***(Does not know = 99:99)***
______________________|__|__| *(Code)*	|__|__|__|__|	|__|__|__|__|	|__|__| Months per year	|__|__|:|__|__| Hours Minutes
______________________|__|__| *(Code)*	|__|__|__|__|	|__|__|__|__|	|__|__| Months per year	|__|__|:|__|__| Hours Minutes
______________________|__|__| *(Code)*	|__|__|__|__|	|__|__|__|__|	|__|__| Months per year	|__|__|:|__|__| Hours Minutes
______________________|__|__| *(Code)*	|__|__|__|__|	|__|__|__|__|	|__|__| Months per year	|__|__|:|__|__| Hours Minutes
______________________|__|__| *(Code)*	|__|__|__|__|	|__|__|__|__|	|__|__| Months per year	|__|__|:|__|__| Hours Minutes

Physical Activity Codes:

**Walk: 01**	**Bike: 04**	**Tennis: 07**	**Dance: 10**	**Hike: 13**	**Football: 16**
**Run: 02**	**Swim: 05**	**Bowl: 08**	**Volleyball: 11**	**Yoga: 14**	**Martial Arts: 17**
**Aerobics: 03**	**Golf: 06**	**Baseball: 09**	**Soccer: 12**	**Skate: 15**	**Other: 18 (Specify)**

12. During your normal workday (even if work ***[or stay]*** at home), which of the following best describes how you spend your time?
    1) |__| Mostly sitting
    2) |__| Mostly walking
    3) |__| Mostly heavy labor or physically demanding work
    4) |__| Not applicable

13. Evaluation of Anthropomorphic Factors and Physical Activity Module:
    1) |__| Unsatisfactory
    2) |__| Questionable
    3) |__| Reliable

REVISION HISTORY

Version	Effective Date	Description of Changes
3.2	12/06/2010	Initial release with MOP v1.0
3.3	02/24/2011	• Ovary and uterus were added to the list of options for cancer sites in questions 15, 23, 31, 39 47, 55, 61, 69, 76, 83 and 90<br>• Released with MOP v2.0
3.4	07/13/2012	EP01: Socioeconomic and Demographic Module<br>• In question 8, combined 'married' and 'consensual union' into a single option: 'married or living as married'<br>• Modified question 7 to include 'Southwest Asian *and Middle Eastern* (e.g., Turkey, Arabia, Iran, Syria, *Israel*)'<br>• Added question 7a on 'Jewish ancestry'<br>• Added 'well,' and 'stream' as options and specified '*manual* pump' and '*electric* motor' in question 14a and added 'latrine' to options in 14b<br>• Specified the use of ISCO-08 occupational codes instead of ISCO-88 codes<br>• Added 'retired,' 'keeping house or caring for children,' 'unemployed/laid off/looking for work,' 'disabled or on sick leave,' 'student,' and 'volunteer work' to ISCO-08 occupational codes<br>• Modified options in questions 17a and 17b to 'very well,' 'fairly well,' 'not very well,' and 'not at all (unable to read)'<br><br>EP02: Module for Access to Health Services<br>• Reworded questions 2-4 and changed options for each to 'health insurance pays all of the costs,' 'health insurance pays some of the costs,' and 'health insurance pays none of the costs'<br>• Modified 'a type of test to detect cervical cancer' in question 5 to 'a test done to detect cervical cancer, that is, have you ever had a PAP test?'<br>• Preceding questions 9-25, added the following statement: Questions 9 through 25 below refer to mammograms, biopsies, and surgeries and procedures PRIOR TO OR BEFORE current diagnosis<br>• Changed fourth option in question 26 to 'mammogram or another imaging study of the breast'<br>• Added 'or after this mammogram (in the case of a positive screening mammogram, but no symptoms)' to question 29<br>• Deleted 'In this first consultation, did the doctor tell you to have a mammography done?,' formerly question 35<br>• Modified questions 34, 41, and 50 to 'Did you pay for any (none), some, or all of this mammogram from your own pocket?' and their responses to 'None,' 'Some,' and 'All'

Version	Effective Date	Description of Changes
		• For questions 43-53, modified instructions to '…those questions should be asked to all patients except those who did not receive a biopsy (in which case the questions do not apply)'<br>• Modified question 46 to 'How many days did you have to wait to receive a biopsy?'<br><br>EP03: Cancer History Module of Interviewee and Family<br><br>• Modified 'family history' to 'family *health* history' in question 6<br>• Question 7 was modified in Spanish and Portuguese questionnaires only as follows: 'Are your biological parents related?'<br>• In the sentences preceding questions 8 and 16 and the questions themselves, 'biological' was added to modify mother and father, respectively; *e.g.*, 'Is your biological mother still alive?'<br>• Replaced 'tumor codes' with an expanded cancer list based on International Classification of Diseases for Oncology, 3rd Edition (ICD-O-3) codes and NCI Surveillance, Epidemiology, and End Results (SEER) 'Estimated New Cancer Cases and Deaths for 2011' table<br>• Modified questions 11, 19, 27, 35, 43, and 51 to include 'Southwest Asian *and Middle Eastern* (*e.g.,* Turkey, Arabia, Iran, Syria, *Israel*)'<br>• Questions related to a relative's age at present or when they died (questions 9, 17, 25, 33, 41 and 49) were made consistent<br><br>EP06: Hormonal and Reproductive History Module<br><br>• Changed question 3 to 'How old were you at the time of your first birth? How old were you at the time of each of your subsequent births?'<br>• Added 'abortions' and 'miscarriages' as options to question 4.<br>• Added question 8: 'Have you ever tried to get pregnant for a year or more and not become pregnant?'<br>• Modified question 17 to 'At what age did you stop having your menstruation periods (age at menopause)?'<br>• Added definition for 'menopause' before question 19.<br>• In question 21, replaced 'pill' with 'medication'<br><br>EP07: Medical History Module<br><br>• Added 'Chagas disease' and 'Are there any other diseases that you would like to mention? If so, please specify' as options for questions 1 and 2

Version	Effective Date	Description of Changes
		EP08: Module of Habits: Tobacco Smoking<br>• Added 'or lived' to question 19<br>• Changed question 20 to 'What relationship did the person that smoked have with you?'<br><br>EP09: Module of Habits: Alcohol Consumption<br>• For questions 1-5, replaced 'one dose' with 'one drink'<br><br>EP10: Module of Anthropomorphic Factors and Physical Activity<br>• Changed question 6 to '*Other than at work*, have you ever in your life done regular physical activities or exercise, that is, at least once per week, for 3 months or more?'<br>• Added question 10 to Physical Activity section: 'How many months per year did you or do you perform this activity?' Added question 12 to Physical Activity section: 'During your normal workday (even if work at home), which of the following best describes how you spend your time? 1) Mostly sitting 2) Mostly walking 3) Mostly heavy labor or physically demanding work 4) Not applicable'<br><br>General Changes:<br>• Added 'Does not know' option to questions where deemed necessary<br>• Added 'never' option to questions where deemed necessary<br>• For questions related to age, changed 'Does not know' code to '999'<br>• Consistent made the code for 'never' to be 8888<br>• Revised existing skip patterns<br>• Formatted questionnaire and included a Revision History table<br>• Released with MOP v3.0
3.4.1	05/24/2013	Administrative changes:<br>• For clarification purposes, the terminology used in questions that are read to the study participant were changed or modified.<br>• The word 'Interviewee' was replaced with 'Participant' throughout the questionnaire to maintain consistency.<br>• If a question was updated, its corresponding interviewer notes, coding notes, and skip patterns were also modified (if applicable).<br>• Added 'Does not know' option to certain questions.<br>• All 'Does not know' options were assigned the number 9*; i.e.,* 9) Does not know.<br>• The sequence of certain questions was changed in order to improve the flow of the interview within a specific section. Other questions were renumbered accordingly.

Version	Effective Date	Description of Changes
		• Incorrect skip patterns and grammatical errors were corrected.<br>• The skip pattern references were changed from 'Skip to' to 'Go to' in a consistent manner.<br>• The questionnaire was formatted and the Revision History was updated.

NOTE: Versions prior to v3.2 were circulated as drafts.
